# What has Happened to Job Quality in Britain? The Effect of Different Weighting Methods on Labour Market Inequalities and Changes Using a UK Quality of Work (QoW) Index, 2012–2021

**DOI:** 10.1007/s11205-025-03542-9

**Published:** 2025-02-11

**Authors:** Thomas C. Stephens

**Affiliations:** 1 Centre for Analysis of Social Exclusion, Sir Arthur Lewis Building, 32 Lincoln’s Inn Fields, London, WC2A 3PH UK; 2 Department of Social Policy, London School of Economics and Political Science, Houghton Street, London, WC2A 2AE UK; 3 LSE School of Public Policy, London School of Economics and Political Science, Houghton Street, London, WC2A 2AE UK

**Keywords:** Job quality, Labour market inequalities by region, Age, Ethnicity and gender, Self-employment, Gig economy, Platform labour and insecure jobs, Synthetic multidimensional indices, Weights, Worker wellbeing

## Abstract

There has been a growth in the use of multidimensional job quality indices, yet the job quality agenda has had a limited impact on public policymaking. This has partly been attributed to disagreements over how to measure job quality and, in particular, weight different indicators of indices. A further reason is a tendency to use international indices, which lack the sample size to explore important country-level inequalities in job quality. To address these issues, this paper presents findings from four different weighting methods for a new synthetic index of the Quality of Work (QoW) for the United Kingdom, using data from a large national survey (Understanding Society). The UK QoW Index contains 7 dimensions and 15 indicators. Several novel indicators argued to be particularly important to the UK context are developed, including health & safety and long-term job prospects. The paper defaults to a widely-used equal weighting approach informed by the Alkire-Foster method, but simultaneously presents findings using alternative hedonic, frequency-based and data-driven weighting methods. The paper then analyses inequalities and changes in job quality from 2012 to 2021; and differences in job quality by type of employment (self-employed, platform labour or gig economy), previous employment status (prior unemployment spell), sex, age, ethnicity and region, according to these four weighting methods. Save for hedonic weighting, these show a broad consistency in many of the key findings: namely, inequalities in job quality between most of the same sub-groups; and a growing polarisation in job quality between employees and self-employed workers.

**Supplementary Information:**

The online version contains supplementary material available at 10.1007/s11205-025-03542-9.

## Introduction

Over the past two decades, there has been a growing interest in what can be done to measure and improve the quality of paid work in modern societies (hereinafter referred to as “job quality”). This most recent spate of interest was instigated by the International Labour Organisation (ILO) in the late-1990s (ILO, 1999), but the European Union followed shortly after with the adoption of the “more and better jobs” agenda (Eurofound, 2012; European Commission, 2003, 2001). Continued interest has perhaps been spurred on by transformative changes in modern labour markets—including the growth of new and more precarious forms of work in the global north (e.g. see Kalleberg, 2009); and its continuing prevalence in many countries in the global south, posing a problem for the sustainability of welfare systems (e.g. see OECD, 2023).

Job quality needs to be measured to be improved. This requires national and international statistics to be developed to monitor changes in job quality over time; investigate inequalities in job quality within and between countries; and identify which workers, and with which characteristics, are in the lowest-quality jobs. Without such measures, there will continue to be a predominance of indicators which, whilst important, do not capture the full range of ways work impacts peoples’ wellbeing on their own—such as hourly wages (Muñoz de Bustillo et al., 2011) or the quantity of jobs (Sehnbruch, 2004). There is widespread agreement that job quality is “inherently multidimensional” (e.g. see Gerstenberger, 2023; also OECD, 2017a, p. 98; Shahidi et al., 2023, p. 786), and thus needs to be measured using multiple indicators. These are usually aggregated into dimensions, which in turn are aggregated into an index. Hence, in tandem with this growing interest in job quality, we have seen the proliferation of multidimensional job quality indices from a range of national and international contexts—particularly in Europe (Cascales Mira, 2021; European Foundation, 2002; Leschke et al., 2008; Leschke & Watt, 2014; Muñoz de Bustillo et al., 2011; Smith et al., 2008), but increasingly also in other contexts such as South and Central America (González et al., 2021; Huneeus et al., 2015; Inter-American Development Bank, 2017; Ortega Díaz, 2011; Sehnbruch et al., 2020; Soffia, 2018; Villatoro S. et al., 2024), the UK (Dobbins, 2022; Irvine et al., 2018; ONS, 2019, 2022a), and at a more global level (Green, 2025; Hovhannishan et al., 2022).

Yet despite this progress, the job quality agenda is generally held to have had a limited impact on at least European public policymaking (Piasna et al., 2019). This is in part because of a lack of a clear consensus about how job quality indices should be constructed and weighted (ibid, p. 179). This mirrors similar issues with weighting in the wider literature on multidimensional wellbeing: research has suggested the weights used do often affect the conclusions that can be drawn from the data (Greco, 2018), yet the tendency across wellbeing indices is to apply equal weighting of all dimensions, which has been criticised (Decancq & Lugo, 2013). In addition, existing job quality literature has tended to be international in focus, with a relative scarcity of national job quality indices. Within European research, the most common approach has been to use the European Working Conditions Survey (EWCS) (Leschke & Watt, 2014, p. 4), which lacks the sample size to explore many within-country inequalities in job quality (and consequently the implications different weighting methods have for these inequalities).

This paper takes steps towards addressing these limitations by investigating job quality over the past decade within a single country context: the United Kingdom (UK). I introduce a new synthetic index of the Quality of Work (QoW), built using a large-scale survey (Understanding Society), representative of all those in paid work in the UK. I investigate changes in job quality over time, and horizontal inequalities (see Stewart, 2005) in job quality between a wider range of sub-groups than is often possible using international surveys.

The main contribution of this paper, however, is in *the use* this data is put to: rather than simply presenting findings using a single weighting method, the paper presents inequalities and changes in UK job quality simultaneously using four different weighting methods, which are designed to reflect different normative theories of wellbeing and methodological approaches in the literature. The first weighting method is based on the Alkire-Foster Method, and weights each indicator and dimension equally—this is identified as the default weighting method because it is increasingly widely used in job quality indices (see especially González et al., 2021; Hovhannishan et al., 2022; Ortega Díaz, 2023; Sehnbruch et al., 2020). However, findings are then also presented using three additional weighting methods discussed in the literature: a “hedonic” weight based on the effect of changes in scores on each indicator on workers’ job- and life- satisfaction; a “frequency-based” weight based on an inverse of average scores on each indicator, which effectively assigns a higher weight to workers who score distinctly worse on those indicators where other workers tend to score better; and a “data-driven” weight based on the factor loadings of the principal components of the index.

The choice of the UK as a country of study was motivated by several considerations. Most importantly, the UK has seen unprecedented interest in job quality in recent years. In response to growing concerns about the impact of technology on future employment and of new and more insecure forms of labour, the then-Government commissioned the Taylor Review of Modern Working Practices in 2017 (Taylor, 2017)—although progress in publishing regular statistics on multidimensional job quality has been slow despite the Taylor Review recommending this over six years ago (Taylor, 2017, p. 11). The recently-elected Labour Government has also shown a greater interest in improving job quality. Its General Election manifesto committed to delivering the highest sustained economic growth in the G7, together with “good jobs and productivity growth in every part of the country” (The Labour Party, 2024, p. 13). Over the past five years, the UK has seen considerable change in its labour market. In the quarter before the first lockdown (Dec 2019–Feb 2020), the UK achieved the highest employment rate since records began in the 1970s (ONS, 2024a), and the unemployment rate was down to levels not seen since 1974 (~ 3.9%) (ONS, 2024b). Due to a focus of successive Governments on improving statutory minimum wages, the position of workers at the bottom of the wage distribution has improved over the past decade (Cominetti et al., 2023). Yet the post-pandemic experience has reversed some of these trends, exposing key underlying weaknesses in the country’s labour market. Part-time employment and consequent low pay continue to be a problem: the proportion of self-employed workers working part-time has risen from 17.3% of workers when records began (Mar-May 1992) to 31.8% now (Mar-May 2024)—reaching a record-high 34.5% in Dec 2022–Feb 2023 (ONS, 2024c), although there has been an unexplained fall in self-reported self-employment in the years since the lockdown (Brown et al., 2022). Linked with this, the proportion of recipients of Universal Credit who are in paid employment in most recent figures (Mar 2024) is still higher than its pre-pandemic level, standing at 2.5 million people or 38% of all Universal Credit recipients (DWP, 2024)—suggesting a continued preponderance of low take-home pay in the labour market. There is a pressing need to study the implications of these changes together in a single index, and study the implications they have for labour market trends and inequalities.

The rest of this paper is split into four sections. First, I briefly set out some requirements for synthetic indices of job quality, and how the QoW index addresses them. Second, I describe indicators and dimensions of the QoW index, the dataset used, and the four different weighting methods. Third, I outline the findings of the paper. Fourth, I conclude with an overview of key findings and future implications.

## Building Job Quality Indices

I begin by outlining four requirements for multidimensional indices of job quality, and setting out how this paper addresses each of these requirements using the UK QoW Index.

### Normative Framework

Every index of job quality involves an initial normative statement about how work relates to peoples’ wellbeing, quality of life, or some other ‘good’ outcome of interest. This requires a definition this ‘good’ outcome, and a discussion of the role that job characteristics play in the creating or impeding the fulfilment of it. Broadly speaking, two opposing approaches exist in the literature. Liberal approaches define job quality in terms of its impact on subjective wellbeing measures such as job- or life- satisfaction (e.g. see Schokkaert, 2007), or alternatively what workers themselves value as important (e.g. Clark, 2015). An alternative set of approaches emphasise more objective measures of wellbeing. Perhaps the most popular version of this latter philosophy is the Capability Approach, which defines wellbeing in terms of (a) what people are able to do and be (Functionings); (b) their freedom to achieve other combinations of beings and doings (Capabilities); after accounting for (c) the different rates at which individuals convert resources into beings and doings due to their personal, social and environmental circumstances (Conversion Factors) (Nussbaum, 2011; Sen, 1992, 1999; for an overview, see Robeyns, 2017). Other objective philosophies exist, however, such as the earlier Scandinavian level of living approach (Erikson, 1974, 1993). They tend to share a scepticism of the role of subjective measures of wellbeing, highlighting peoples’ ability to adapt to disadvantageous circumstances (Sen, 1987, pp. 45–47); and argue that resources, particularly income or wages, whilst important, are insufficient measures of picture of poverty or wellbeing (Sen, 1999, p. 87).

This paper draws from a normative framework for measuring job quality using the Capability Approach (Stephens, 2023a), which defines job quality in more objective terms based on the impact of work on the achievement of important Functionings. The Capability Approach is widely used in job quality literature (Green, 2009; Sehnbruch, 2004; Soffia, 2018), and applications of the approach tend to emphasise the use of objective over subjective job quality indicators (e.g. see Felstead et al., 2019). Despite these fundamental differences between objective and subjective approaches, it has been argued there is a “remarkable consensus” in terms of the importance of the key indicators, with both approaches emphasising the measurement of “variety in the task, the level of personal initiative that can be exercised, the degree of participation at work, and the extent to which the job permits personal self-development” (Gallie, 2003, p. 65). Both approaches have also placed increased emphasis on the importance of job security and career development opportunities (Gallie, 2003, pp. 62–63) following the end of full employment in Western societies since the 1980s (Gallie et al., 1995). However, there is a risk of under-stating the continued differences. Direct measures of subjective job satisfaction do not always align with what objective indicators tell us about job quality (e.g. see Clark, 1997; Léné, 2019), and subjective indicators widely criticised by proponents of more objective indicators (Brown et al., 2007; Green & Tsitsianis, 2005; Hamermesh, 2001; Muñoz de Bustillo & Fernández Macías, 2005). Both approaches also have reason to disagree about the *relative weights* to be assigned to different indicators, as I will demonstrate later.

### Indicator Selection and Construction

Indicators are the building blocks of any multidimensional index: scores on indicators ultimately determine index scores, and thus our assessment of an individual’s job quality. This involves two considerations: indicator *selection* and indicator *construction*. The challenge of indicator *selection* should not be under-stated. Issues of survey construction and variable availability limit the choice of indicators. Effective indicator selection also requires careful consideration of the legal and societal environment in which people are working, since some indicators will be more important in some contexts than others. For example, Kalleberg (2018, p. 30) observes that the importance of different indicators of job precarity will depend on the policy and statutory environment in a country, since “policies that impose austerity by removing or decreasing economic or social benefits … will also lead to precarious work, whether the employment contract is temporary or not.” Workplace pensions indicators will be more important in societies with inadequate state-provided pensions (Barr & Diamond, 2010). The sufficiency of earnings to meet some societally-agreed standard will depend on the cost of goods and services in a country, and whether services such as healthcare are free at the point of use or paid for through other means. This paper therefore constructs indicators based on consideration of the specific UK context (see Sect. 3.2 and Online Appendix F).

Indicator *construction* is generally framed in terms of the transformation function used to standardise the values of the selected indicators and turn them into indicator scores, ready to be aggregated into a multidimensional index (Decancq & Lugo, 2013, pp. 11–14). This, again, involves some normative considerations such as whether there is declining marginal utility (i.e. diminishing returns to higher values in a variable); what role the distribution of values within each variable should play in determining indicator scores; and, more fundamentally, what the index is designed to measure. For example, the use of a cut-off approach using binary indicators—where each indicator has only two possible scores, deprived and non-deprived—tends to be favoured for more poverty or deprivation-based measures of job quality (e.g. see González et al., 2021). Alternative approaches using categorical or continuous indicators are also used, especially for indicators where a binary cut-off is misleading or impossible (Cerioli & Zani, 1990; Cheli & Lemmi, 1995; Deutsch & Silber, 2005).

To inform indicator construction, this paper suggests that the concept of job *quality* is best captured using a broader wellbeing-based approach. When we talk about work-related *wellbeing*, we are viewing jobs along a spectrum of wellbeing achievement, as distinct from concepts such as poverty or deprivation which focus on the identification and study of a smaller subset of the working population. To operationalise this, I draw some lessons from the Totally Fuzzy Approach to indicator construction (Cheli & Lemmi, 1995). The method originated in poverty research out of a need to capture individuals’ *proximity* to a deprivation cut-off, rather than simply writing-off all individuals who are above this cut-off. The same principles can be used for the QoW index.

Let ${X}^{ij}$ denote the score of individual i on indicator j of the QoW index, which can range from 0 (lowest work-related wellbeing on indicator j) to 1 (highest work-related wellbeing on indicator j). Let ${\psi }_{ij}$ denote the ‘raw’ (i.e. non-standardised) value of indicator j for individual i. ${\psi }_{j min}$ denotes the value needed to achieve the minimum possible score for an individual on indicator j (i.e. ${X}^{ij}=$ 0), and can be seen as a poverty or deprivation cut-off.${\psi }_{j max}$ denotes the value needed to achieve the maximum possible score for an individual on indicator j ${(X}^{ij}=$ 1). For most indicators in the QoW index, a number of possible scores in-between these minimum and maximum values exist, as follows.^1^

**Binary indicators** have just a minimum cut off ${\psi }_{j min}$, which is in line with deprivation-based indices of job quality. Only a small number of indicators in the QoW index are binary. Their scores are therefore determined simply by the following notation:

$${X}^{ij}=0 (Worst) if {{\psi }_{ij}\le \psi }_{j min}$$

$${X}^{ij}=1 (Best) if {{\psi }_{ij}>\psi }_{j min}$$

**Categorical indicators** also have a maximum cut-off,${\psi }_{j max}$, and thus have three possible scores. These constitute the majority of indicators in the QoW index, and allow for the identification of a middle-scoring part of the population who achieve more than the minimum cut-off but below the maximum cut-off. They thus are above the minimum deprivation threshold, but are still unable to achieve the work-related wellbeing enjoyed by a large proportion of the population and so should still be of some concern for policymakers interested in improving job quality. Scores for categorical indicators are determined by the following notation:

$${X}^{ij}=0 \left(Worst\right) if {{\psi }_{ij}\le \psi }_{j min}$$

$${X}^{ij}=0.5 (Middle) if {\psi }_{j min}< {{\psi }_{ij}< \psi }_{j max}$$

$${X}^{ij}=1 \left(Best\right) if {{\psi }_{ij}\ge \psi }_{j max}$$

**Continuous indicators** have more than three possible scores. These are used in a minority of indicators when work-related wellbeing changes in line with where ${\psi }_{ij}$ is in the distribution of all ${\psi }_{j},$ with considerations such as declining marginal utility not coming into the picture. For these indicators, ${\psi }_{j}$ are first converted into standard units by deducting them from the mean and dividing by the standard deviation for the population. ${\psi }_{j min}$ denotes the worst value and ${\psi }_{j max}$ the best value of ${\psi }_{ij}$ for all ${\psi }_{ij}$ in standard units.^2^ The scores in-between these thresholds are simply determined by where the scores are in the distribution^3^:

$${X}^{ij}=0 \left(Worst\right) if {{\psi }_{ij}\le \psi }_{j \text{min}}$$

$${X}^{ij}=\frac{{{\psi }_{ij}- \psi }_{j min}}{{{\psi }_{j max}- \psi }_{j min}}$$

if

$${\psi }_{j min}< {{\psi }_{ij}< \psi }_{j max}$$

$${X}^{ij}=1 \left(Best\right) if {{\psi }_{ij}= \psi }_{j max}$$

### Aggregation and Weighting: The Default Method (Alkire-Foster Informed)

Scores then need to be aggregated into an index. This is usually (though not necessarily) preceded by a stage where similar indicators are first aggregated into dimensions. In both stages, a judgment needs to be made about the weights of the indicators within each dimension, and then the dimensions within the index. Weights should reflect the *substitutability* of different indicators and dimensions and not merely the relative importance of them, since a low score in a higher-weighted is harder to be compensated for by a higher score in a lower-weighted indicator (Decancq & Lugo, 2013, p. 13).

As noted in the introduction, this paper defaults to a weighting method informed by the Alkire-Foster method (Alkire & Foster, 2011a, 2011b; Alkire et al., 2015), because this is increasingly widely used in job quality literature and is therefore a reasonable starting point for setting weights for the index. Whilst originally used for the measurement of poverty, a version of the method has been developed for job quality indices in Central and Latin America (González et al., 2021; Sehnbruch et al., 2020), Spain (García-Pérez et al., 2017), Mexico (Ortega Díaz, 2023), Egypt (Sehnbruch et al., 2021) and at a global level (Hovhannishan et al., 2022).

Under this application of Alkire-Foster, indicators are given equal weighting within each dimension. This means that, consistent with González et al. (2021), the score of a given individual on a given dimension, *S*^*id*^, is simply the sum of indicator scores (${X}^{ij}$) divided by the number of indicators in that dimension (*N*^*jd*^). Note that all dimension scores therefore range from 0 to 1, with 0 meaning the individual scored the lowest in all indicators of a dimension and 1 signifying the highest score in all indicators:

$${S}^{id}=\frac{ {\sum }_{d}^{1}{X}^{ij}}{ {N}^{jd}}$$

The dimensional scores are then added together into an index score for each individual, ${C}^{i}$, which can be represented as the weighted sum of all ${S}^{id}$:

$${C}^{i}= \sum {S}^{id}\times {W}^{d}$$

However this paper departs from these approaches in terms of the *purpose* for which the index is constructed: these approaches tend to define job quality in terms of “employment deprivation” and are thus focussed more on counting both (a) the *number* of workers employment deprived and (b) the *intensity* of their deprivation based on their scores on exclusively binary indicators, with a single cut-off. Since this paper defines job quality using a more wellbeing-based normative framework, the focus of this paper is instead on measuring workers’ job quality along a spectrum of wellbeing achievement, using a mix of binary, categorical and continuous indicators. A second point of departure relates to the weighting of the employment dimension. Although the tendency in Alkire-Foster is to weight all dimensions equally within the index, many applications of Alkire-Foster to job quality assign a higher importance to earnings (González et al., 2021; Muñoz de Bustillo et al., 2011, p. 152; Sehnbruch et al., 2020). Most often this is done by adopting a criterion where anyone deprived in the earnings dimension is classed as deprived overall. To reflect this in this paper, the earnings dimension is double-weighted, at 25% of the QoW index. Beyond this, and again consistent with the existing applications of Alkire-Foster to job quality, all other dimensions are weighted equally. The minimum index score ${C}^{i}$ will be 0, reflecting the lowest possible score on all indicators of every dimension, whilst the maximum will be equal to the weighted sum of the number of dimensions.

### Methods of Analysis: The Implications of Different Weighting Methods

A fourth and final requirement is to specify the methods and purpose of analysis. The above conclusions naturally give rise to several ways of presenting and analysing the data from the index. Because very few indicators in the QoW index are binary, this paper departs from more deprivation-based measures used in literature such as Alkire-Foster (Alkire et al., 2015), and thus focuses on the following three methods of analysis:

- **Uncensored indicator headcount ratios.** For binary and categorical indicators these are the proportion scoring Worst and (if applicable) Middle, whereas for continuous indicators they are represented as the proportion scoring ≤ 0.5 (Worst) and > 0.5 (Best). The term is drawn from the Alkire-Foster literature (Alkire et al., 2015, pp. 156, 167). This method of analysis does not involve any comparison of different weighting methods.
- **Mean QoW Index, dimension and indicator scores.** These allow us to explore changes in QoW over time and differences between sub-groups. Higher mean scores mean higher QoW. Where QoW index scores are compared, the four different weighting methods are presented alongside each other.
- **Net percentage difference in mean QoW index scores.** This gives a picture of inequality in QoW calculated by dividing the mean QoW score of a range of sub-groups versus the mean of a consistent comparator sub-group. The higher the percentage, the greater the inequality in mean QoW index scores between the sub-group and the comparator. All these are presented using all four weighting methods simultaneously.

The focus of this paper is the effect of three alternative weighting methods which depart from the default method (see Sect. 3.4). As such greater emphasis is placed on those methods of analysis where the four different weighting approaches can be presented alongside each other.

## The UK QoW Index: Data and Indicators

### The Dataset

The QoW index uses data from Understanding Society, also known as the UK Household Longitudinal Study. Understanding Society is one of the largest panel surveys in the world, and interviews adults aged 16 and over in a representative sample of UK households, with most interviewed annually over overlapping 24-month waves (UK Data Service, 2015). Weighting methods have been introduced to allow it to be used for representative cross-sectional analysis, and to correct for survey design and non-response biases (Kaminska & Lynn, 2019; Lynn, 2011). Understanding Society asks questions on job quality in every other wave. The QoW index therefore consists of everyone in paid work, or away from paid work in the previous week, in Waves 4 (2012–13),^4^ 6 (2014–15), 8 (2016–17), 10 (2018–19) and 12 (2020–2021) of the survey. This consists of an unweighted number of 108,973 non-independent respondents, ranging from 23,759 independent respondents in Wave 4 to 15,636 independent respondents in Wave 12.

Understanding Society has several advantages over alternative UK surveys. Its income data has been found to compare well with other national surveys (Fisher et al., 2019), and unlike the UK’s official labour market survey (the Labour Force Survey) it measures self-employed as well as employee earnings. All but one of the indicators for the QoW index has been constructed to include on workers who are self-employed in their main job. In addition, three indicators in the index use data on the earnings and hours worked in *all* paid jobs, and not just main jobs. As will be seen, the sample size is also sufficient to investigate differences in job quality by ethnicity, region, sex and age.

Missing responses are generally low amongst those who respond to the survey (< 5% of weighted respondents in each wave), but exceeds 5% in a number of instances. Missing data for most indicators is therefore imputed using multiple imputation using chained equations, in line with best practice (Azur et al., 2011; Collins et al., 2001; Van Buuren & Groothuis-Oudshoorn, 2011). Online Appendix B contains a full missing values analysis and Online Appendix C outlines the imputation methodology.

Taken together, all the above offers significant advantages over some other job quality indices, which can struggle to include informal, self-employed or insecure workers; exclusively use data on main jobs; lack the sample size to analyse many within-country inequalities in job quality; and deal with missingness through listwise deletion, which can bias results.

### Dimensions and Indicators

**Fig. 1:**
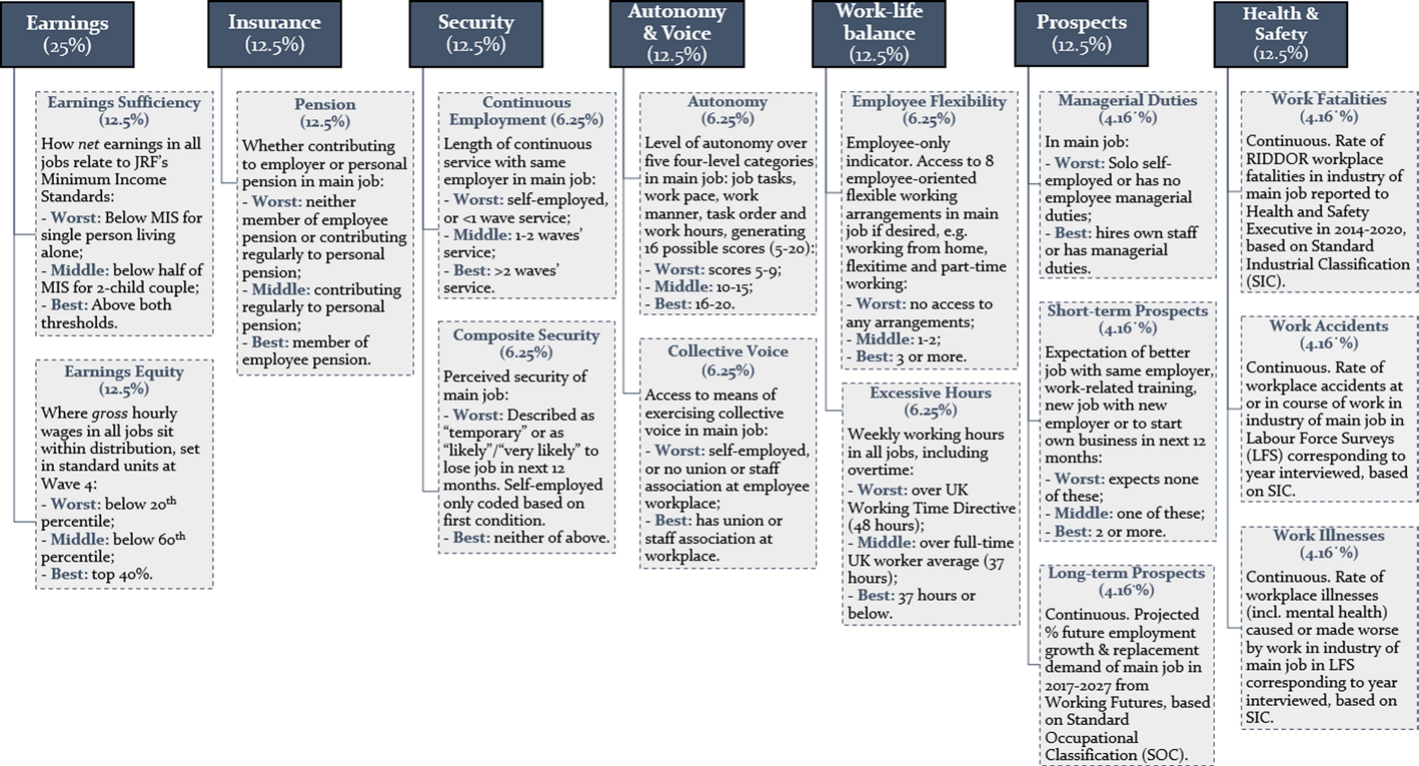
Dimensions, indicators and percentage weights of the UK Quality of Work index. *Note the Flexibility indicator shown as a proportion of employees only, since self-employed are not scored on this indicator

**Fig. 2:**
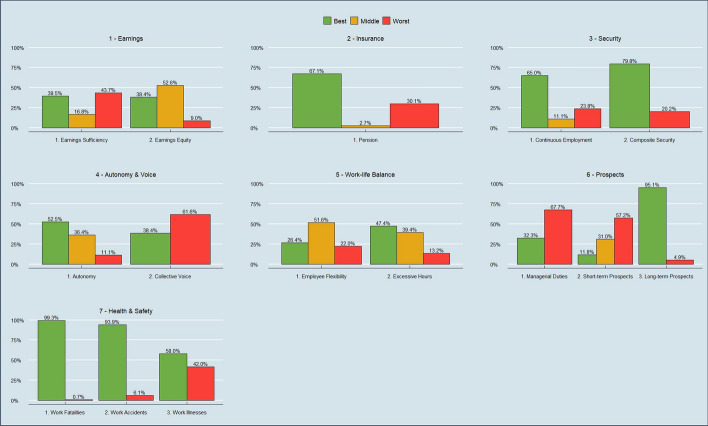
Uncensored indicator headcount ratios as at Wave 12 (2020–21) for all workers in the QoW index

Figure 1 sets out the indicators and dimensions of the UK QoW index, and their percentage weights under the default (Alkire-Foster informed) weighting method (as discussed in Sect. 2.3). There are 3 binary, 8 categorical, and 4 continuous indicators, grouped into 7 dimensions. Figure 2 provides a snapshot of descriptive data on indicator scores at the latest wave available (Wave 12). The index captures many aspects of job quality which are discussed in the literature, but also builds on these in many ways to create indicators which are particularly important to the UK context.

In the Earnings dimension, I make a crucial distinction between two indicators: (a) the sufficiency of *net* earnings to meet some minimum societally-agreed standard, in this case the Joseph Rowntree Foundation’s Minimum Income Standards (Earnings Sufficiency) (Bradshaw et al., 2008; Hirsch, 2015); and (b) where one’s *gross* hourly wages sit within the wage distribution, with a particular focus on those at the bottom 20% of the wage distribution in line with the focus of research (Machin, 2011) (Earnings Equity). This is in line with OECD (Cazes et al., 2016; OECD, 2017b, p. 17) and European (Leschke et al., 2008, p. 10) research, but the quality of the earnings and working hours data in the index allows me to make this distinction more clearly than some other studies. The use of a *net* income-based indicator, with the Minimum Income Standards, enables the index to capture changes in the cost of living and/or societal norms about the minimum acceptable standard of living, in ways not possible by simply by comparing gross wages.^5^ Alongside this, a separate Pensions indicator captures whether workers are contributing to a workplace pension, or if not, a personal pension. Its relatively high weighting in the default (QoW Index) weighting method, due to its placement in its own distinct dimension, reflects its particular importance in the UK, with the state pension inadequate on its own to provide citizens with a decent standard of living (see Barr & Diamond, 2010).

The Autonomy indicator captures measures of task autonomy which receive emphasis in both strands of job quality literature (Gallie, 2003; Gallie et al., 2004), whilst Collective Voice measures on aspect of employee voice exercised through trade unions and staff associations which has been the subject of long-standing interest in literature on worker voice (Bennett & Kaufman, 2007; Boroff & Lewin, 1997; Freeman & Medoff, 1992). Finally, the Work-Life Balance dimension reflects the literature on work-family and family-work conflict (Annor & Burchell, 2018; Esping-Andersen, 1996; Gallie, 2007; Parasuraman & Simmers, 2001) using two indicators: an indicator comparing hours worked in all jobs compare with the UK Working Time Directive of 48 h (Worst) or the average for full-time workers of 37 h (Middle); and an employee-only indicator on the number of worker-oriented flexible working opportunities reports being available in their main job (whether they choose to use them or not).

Two dimensions on Security and Prospects capture the growing role of these two issues in job quality since the 1980s. This is particularly reflected in the Combined Prospects, Managerial Duties and Short-Term Prospects indicators, which respectively capture workers’ perceived job security; whether they have supervisory duties or (if self-employed) hire their own staff; and their perceived likelihood of accessing training, getting a better job, finding a promotion or starting a business. These are supplemented by two more novel and objective indicators. Continuous Employment uses longitudinal data to measure employees length of continuous service with the same employer. This is aligned to the UK’s framework, where many protections for workers are based on length of continuous service (with self-employed workers denied these protections) (Brione, 2022).^6^ Long-Term Prospects uses Department for Education data from Working Futures (DfE, 2020; Wilson et al., 2020) on the projected replacement demand and employment growth of each occupational group over the coming decade (2017–2027) by 2-digit Standard Occupational Classification (SOC). This provides an estimate of the replacement rate (retirement and exit of current workers) and projected employment growth of their occupation – and thus their vulnerability to lay-offs due to technological change and low demand for workers in their profession. The methodology for creating this indicator is set out in Online Appendix E.

Finally, a dimension on workplace health and safety is also introduced. Understanding Society contains no questions on health and safety, but indicators on workplace fatalities, accidents and illnesses are introduced by matching incidence rates from the Health and Safety Executive and LFS by workers’ Standard Industrial Classifications (SIC). Online Appendix D sets out the methodology for doing this.

### Alternative Weighting Methods

Three variations to the weights set out in Fig. 1 are explored. These weights are designed to reflect the sensitivity of this paper’s findings to some reasonable alternative views about how the index should be constructed:

- **Hedonic weighting.** This is designed to reflect the weights of a more liberal normative framework. Taking advantage of the longitudinal nature of Understanding Society data, the hedonic weights are informed by first-difference fixed effects regressions of the effect of changes in scores on each indicator in the QoW index on changes on both life- and job- satisfaction. This allows me to control for time-invariant unmeasurable individual idiosyncrasies and characteristics, and replicates a proposal set out in Schokkaert (2007) and Schokkaert et al. (2009). The standardised coefficients, where significant and consistent for both measures, are used to determine the weights of each indicator, with the life satisfaction coefficients weighted 2/3rds to reflect its higher importance to wellbeing.
- **Frequency-based weighting.** This assigns a *higher* weight to those indicators with the *best* mean scores in Wave 4, i.e. the lowest proportion of people scoring poorly in them. This replicates a weighting proposal in poverty research (Cerioli & Zani, 1990; Cheli & Lemmi, 1995; Deutsch & Silber, 2005).
- **Data-driven weighting.** This weights indicators according to the amount of variance they explain in the data, using Principal Component Analysis (PCA). I take a weighted average of the factor loadings of those principal components which explain 90% of the variance. Only positive factor loadings are used. PCA is widely used in the literature (e.g. see Cascales Mira, 2021; McGillivray, 2005; Noorbakhsh, 1998), and its use in this paper is similar to Greco (2018, p. 464).

Table 1 sets out the percentage weights of these three alternative weighting approaches. Online Appendix A provides fuller detail on how these weights were constructed, and discusses the advantages and disadvantages of the normative assumptions underlying them.

**Table 1:** Percentage weights for hedonic, frequency-based and data-driven weighting methods for the QoW index. Full methodology explained in Online Appendix A

Indicator	QoW Index weight (Alkire-Foster based)	Hedonic weight	Frequency-based weight	Data-driven weight (PCA)
Earnings Sufficiency	12.50%	6.49%	5.62%	14.4%
Earnings Equity	12.50%	7.61%	6.32%	12.5%
Pension	12.50%	0%	5.67%	7.7%
Continuous Employment	7.50%	0%	7.02%	8.0%
Composite Security	7.50%	32.06%	8.29%	4.8%
Autonomy	7.50%	31.75%	7.06%	6.0%
Collective Voice	7.50%	0%	5.42%	5.5%
Employee Flexibility	7.50%	9.36%	5.88%	5.4%
Excessive Hours	7.50%	9.86%	6.61%	2.3%
Managerial Duties	4.16%	0%	5.10%	9.6%
Short Term Prospects	4.16%	0%	5.11%	0.8%
Long Term Prospects	4.16%	0%	8.15%	4.7%
Work Fatalities	4.16%	0%	11.36%	5.0%
Work Accidents	4.16%	2.87%	6.27%	6.2%
Work Illnesses	4.16%	0%	6.14%	7.0%

## Findings

### Headline Time Series Changes: Employees vs. Self-employed

**Fig. 3:**
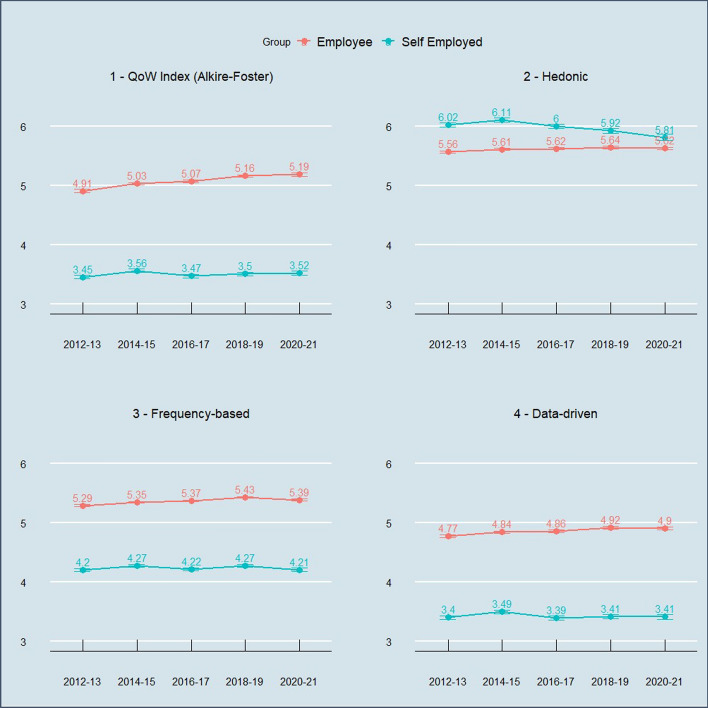
Time series of mean QoW index scores by weighting method, broken down by employees vs. self-employed, 2012–13 to 2020–21. Error bars show standard errors of the weighted means

**Fig. 4:**
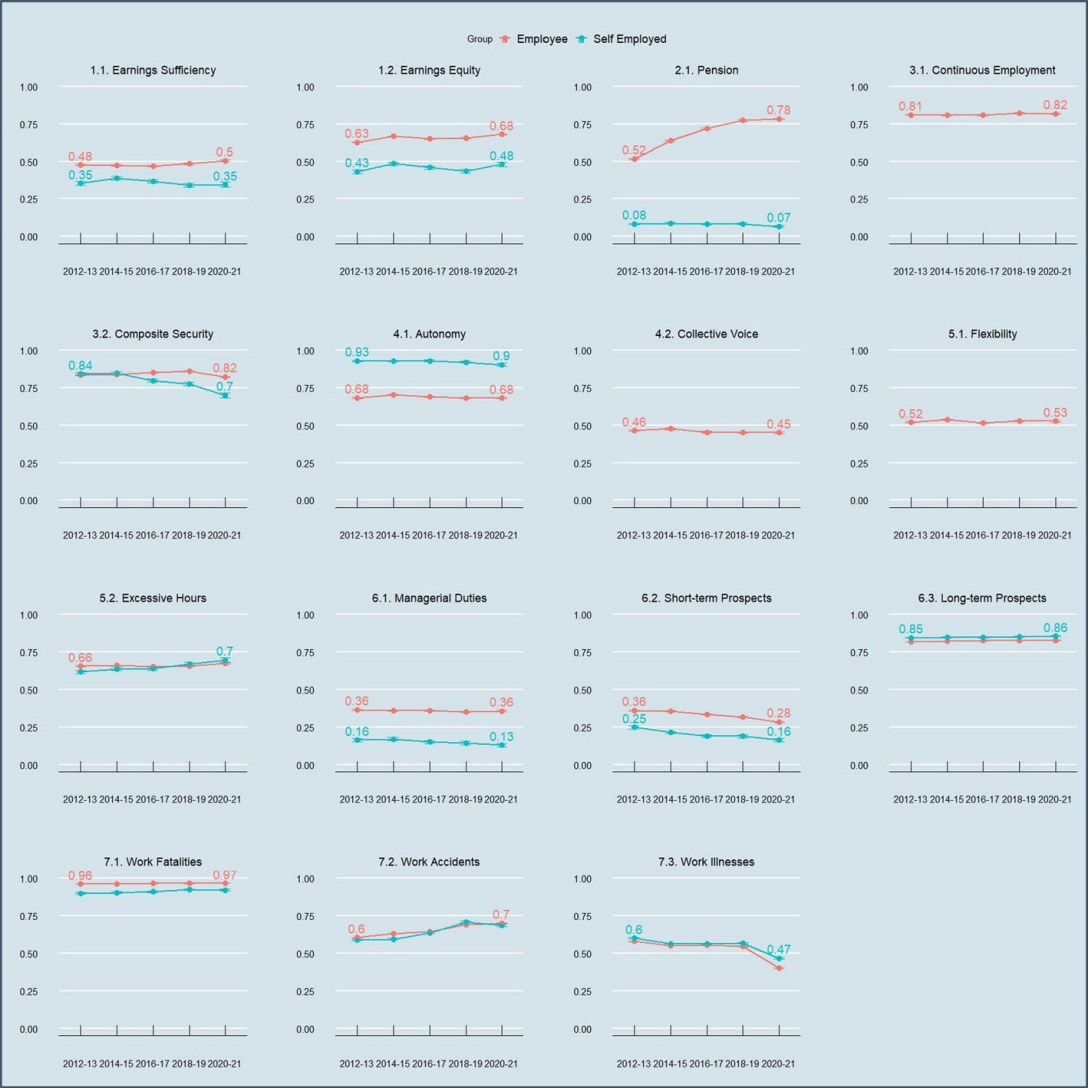
Time series of mean QoW indicator scores broken down by employees vs. self-employed, 2012–13 to 2020–21. Error bars show standard errors of the weighted means.*All scores converted to a 0-1 scale to aid comparison; this does not reflect their weighting in the QoW index. Self-employed scores not included in Continuous Employment, Collective Voice and Flexibility indicators due to automatic scoring (=0) for the first two indicators and no self-employed data on the latter indicator

**Fig. 5:**
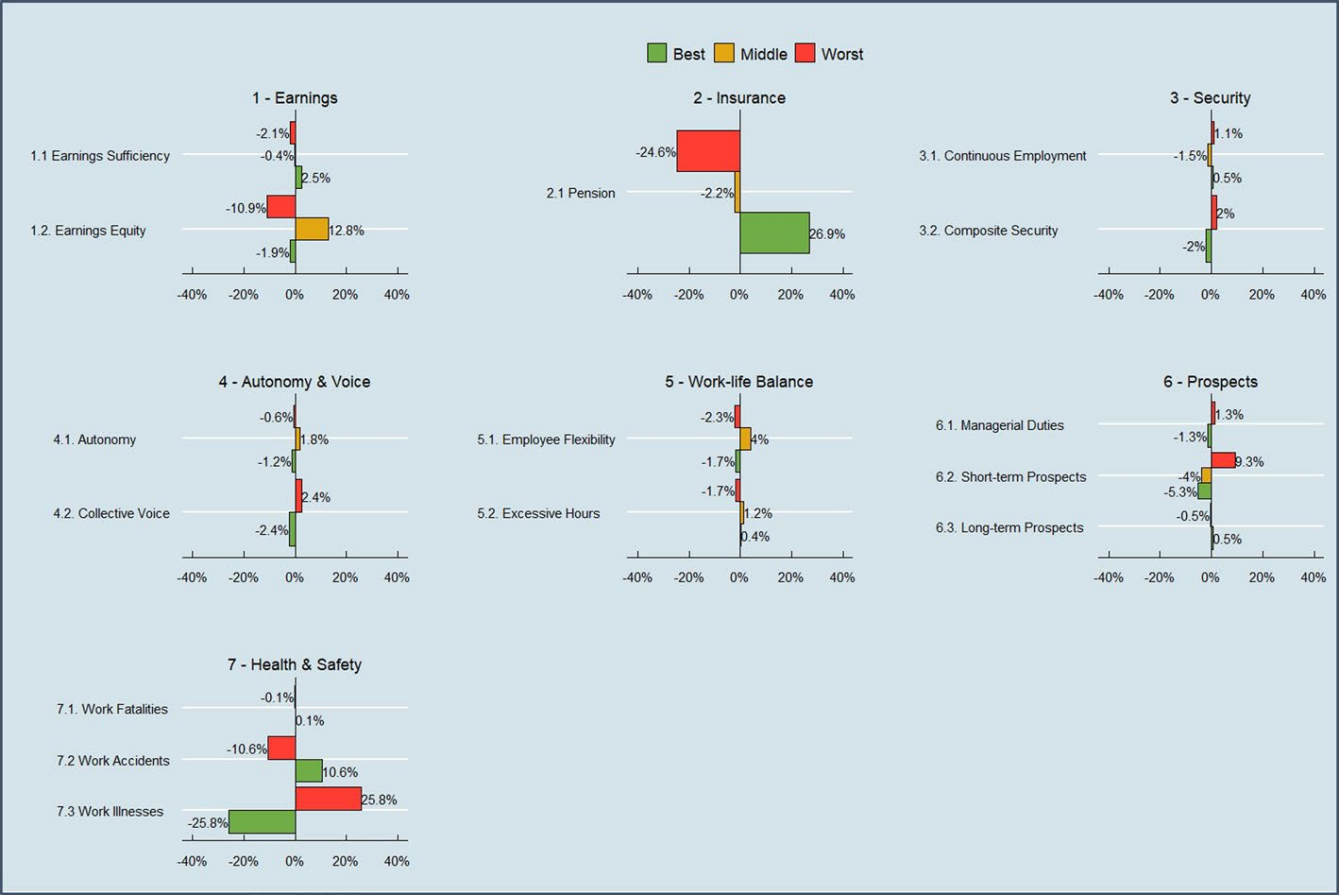
Employee net change in uncensored headcount ratios, 2020–21 minus Wave 4 2012–13

**Fig. 6:**
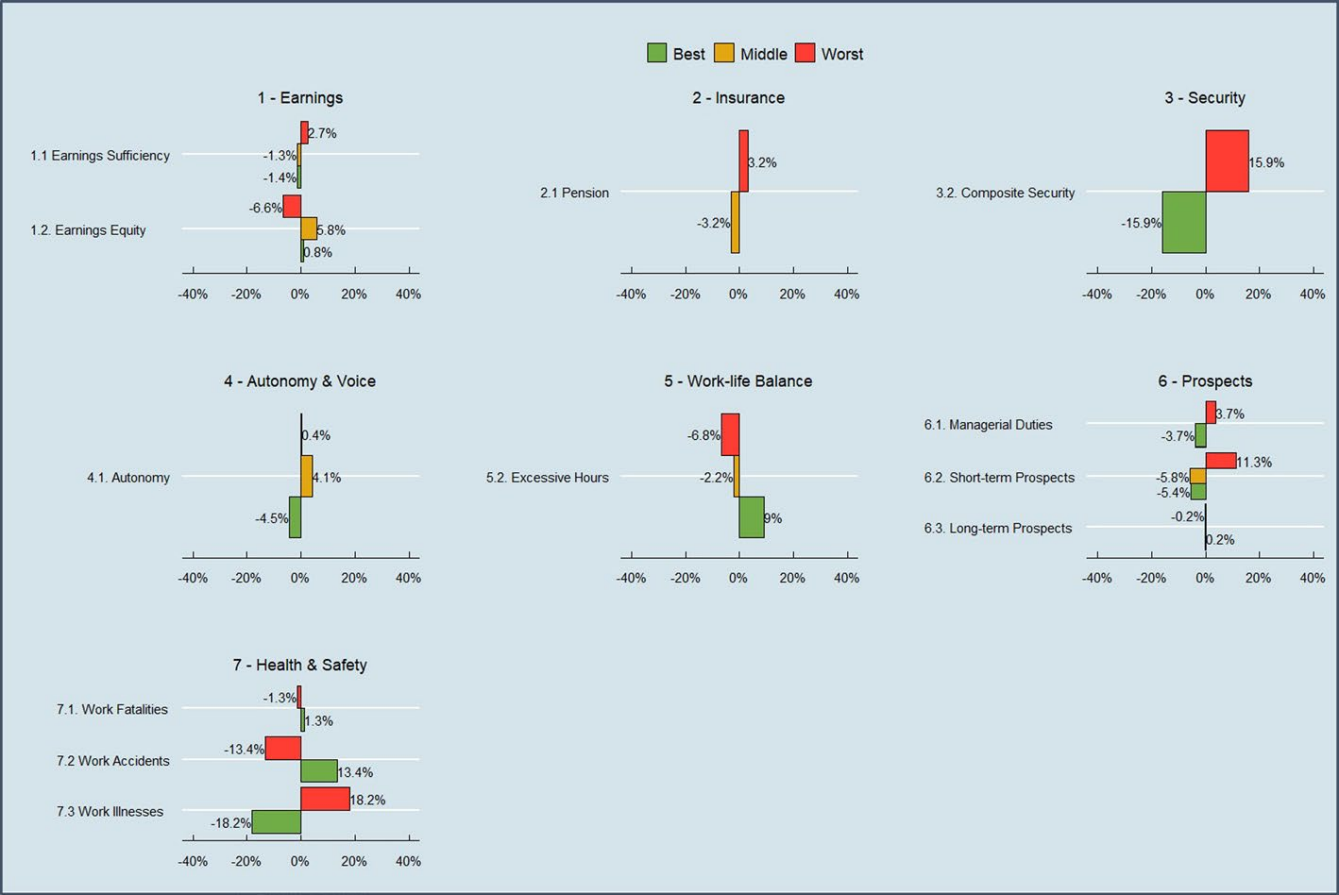
Self employed net change in uncensored headcount ratios, 2020–21 minus 2012–13.*Note net change in the continuous indicators is not reflected in indicator scores in the same way as the binary and categorical indicators, since many workers have scores in-between 0 and 0.5 and 0.5–1. This should be borne in mind when interpreting net change for these particular indicators

I begin with an overview of key changes in QoW in the UK. Because of the stark differences in the nature and trends in job quality between employees and self-employed workers, these are presented separately. Figure 3 presents a time series of mean QoW index scores by weighting method for these two groups. Figure 4 provides a more detailed picture of changes in mean QoW by each indicator of the QoW index, weighted according to the first weighting method only. Figures 5 and 6 provide an even more detailed picture, showing net changes in uncensored headcount ratios in the QoW indicators between 2012 and 13 and 2020–21, again broken down by employees and self-employed.

There are some positive findings for the UK labour market. All four weighting approaches agree that employee job quality has risen to at least some extent over the past decade. This has been particularly driven by a marked rise in employee pension enrolment following the introduction of the Pensions Act 2008. This is consistent with what other national statistics show (ONS, 2022b), and explains the greater improvement in employees’ positions in the Alkire-Foster based weighting method (which assigns a higher weight to Pensions). It is also driven by a marked improvement in the position of workers in the bottom 20% of the wage distribution (Earnings Equity): a key success story of the UK economy as a result of the long-standing focus on improving hourly wages, and again consistent with what is found in other datasets (Resolution Foundation, 2023). There has also been a fall in workplace accidents, which accelerated by, but not caused by, the Covid-19 pandemic. This appears to reflect a genuine trend in the labour market, and whilst there have been issues with how this data is captured due to the pandemic alternative calculations have shown similar improvements (see HSE, 2021 and Online Appendix D).

Other trends are less positive. Three of the four weighting approaches agree that the self-employed have lower job quality than employees—only hedonic weighting places them more highly due to its greater weighting of measures of autonomy in which self-employed workers score more highly. In addition, there is agreement across all weighting approaches that the position of self-employed workers has declined relative to employees: it has stagnated for two of the weighting approaches, and declined considerably according to hedonic weighting. Whilst the self-employed have seen similar positive trends as employees in Earnings Equity and Workplace Fatalities, and an improvement in Excessive Hours, they have seen a decline in Earnings Equity, Composite Security, Managerial Duties and Pensions. Both employees and self-employed have seen a decline in Short-Term Prospects, and a stark rise in Workplace Illnesses since the Covid-19 pandemic. It is noteworthy that Earnings Sufficiency and Earnings Equity tell a rather different story of the earnings of UK workers over the past decade: self-employed workers have seen a decline in Earnings Sufficiency, and employees have seen only a small improvement. This serves to illustrate that an improvement in gross wages may not always lead to an improvement in the sufficiency of net earnings. Earnings Sufficiency, as opposed to Earnings Equity, depends on the interaction of wages, hours worked, pay deductions, inflation and societally-agreed minimum income standards; and may also be affected by the entry of workers in jobs with low take-home pay into the labour force – such as newly self-employed workers with sporadic access to paid work.

Overall, the net effect of these trends has been to increase labour market polarisation between employees and self-employed workers—or, in the case of hedonic weighting, to move the situation of self-employed workers to near-parity with that of employees. This provides the backdrop to the discussions in the succeeding sections.

### How Does Weighting Affect Inequalities in Job Quality?

**Fig. 7:**
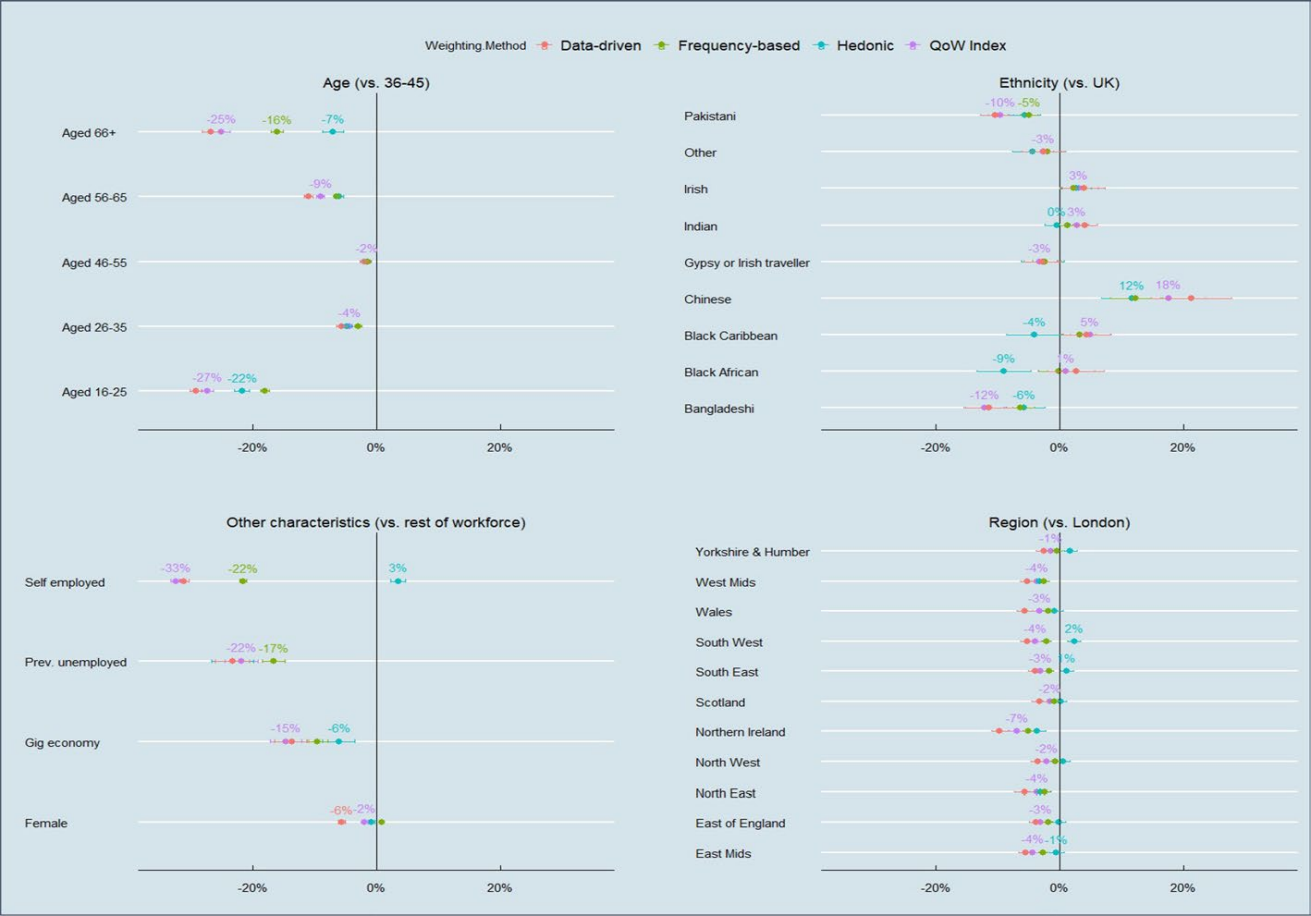
Net percentage difference in mean QoW scores (2020–21) between pairs of sub-groups by weighting method. Standard errors of the weighted mean of the smallest-sized (lowest n) sub-group in error bars

This section analyses the implications different weighting approaches have on inequalities. I focus on studying *differences in mean QoW between sub-groups* in society rather than the *overall distribution of QoW between individuals*—an approach which aligns more with literature on horizontal inequalities (e.g. see Stewart, 2005). Figure 7 presents the inequalities net difference in mean QoW scores in the latest wave of the index (2020–21) for 29 subgroups of paid workers, by age, ethnicity, region and other characteristics. To aid comparison, these percentage differences are represented with reference to one of four common reference groups.

Again, the data shows that there is broad agreement across the weighting approaches in the inequality in job quality in many sub-groups: the weighting approaches disagree over the *size* of the inequality, but not usually over *which* group is worse off. Again, the key exception is hedonic weighting: Black African and Black Caribbean groups have a considerably *worse* QoW according to hedonic weighting, and people aged 66 + and, as discussed, self-employed workers have considerably *better* QoW. Overall, the most pronounced inequalities in QoW are seen with respect to 16–25 year-olds, gig economy workers,^7^ the self-employed, those who had at one unemployment spell since the last wave, some ethnic groups (esp. the Bangladeshi and Pakistani community), and residents of one region (Northern Ireland).

**Fig. 8:**
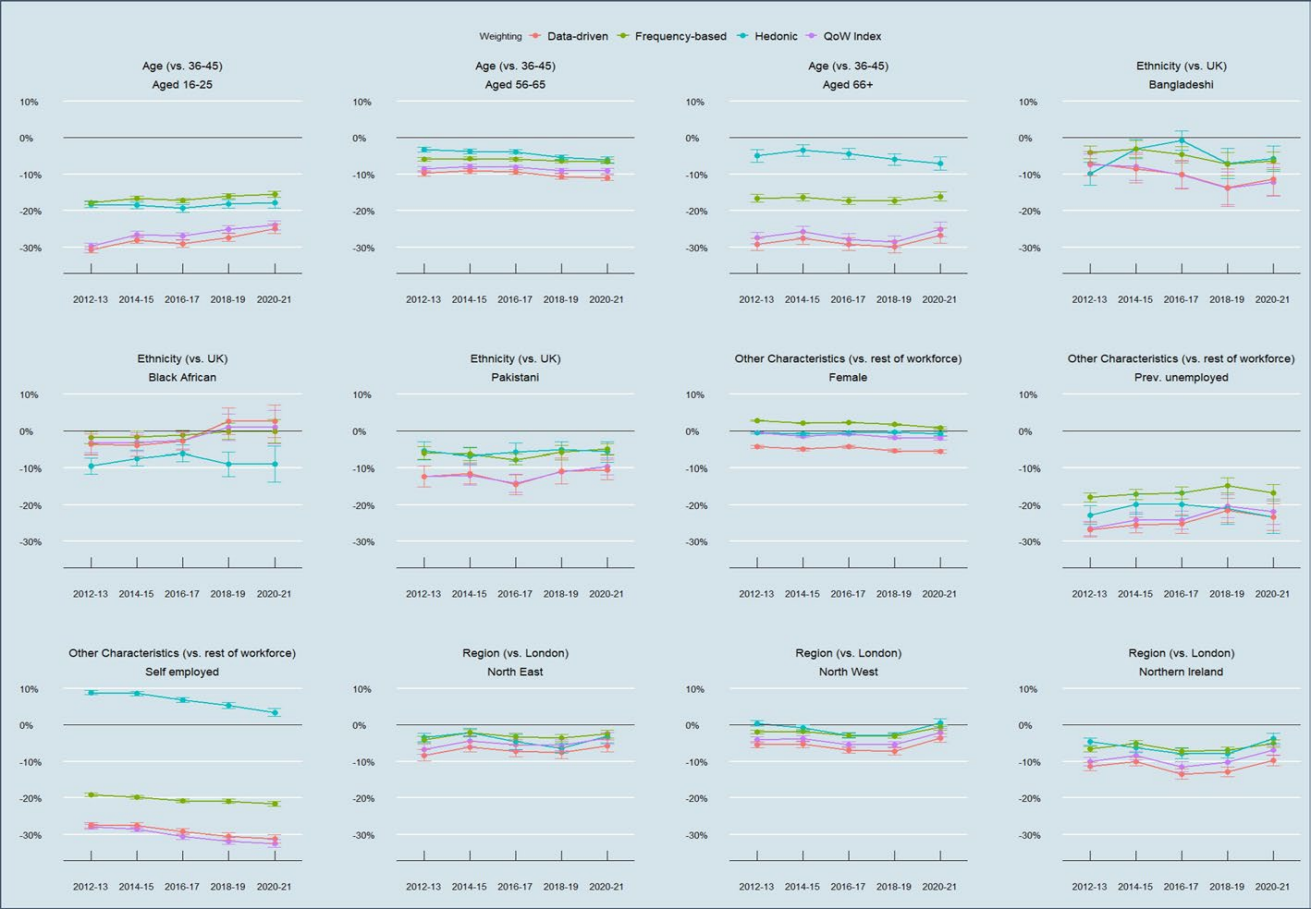
Time series of net percentage differences in mean QoW scores between pairs of sub-groups by weighting method. Standard errors of the weighted mean of the smallest (lowest n) sub-group in error bars

Figure 8 shows how these inequalities have changed over time for 12 sub-groups with the greatest differences in QoW, broken down by weighting method. These show a mixed picture over the past decade. Again, all approaches save for hedonic weighting broadly agree on the trends. The data suggests there has been an improvement for 16–25 year-olds, some regions, and potentially also people of Black African ethnicity (although hedonic weighting suggests the opposite for the latter group). However, all approaches agree that the position of the self-employed and to a lesser extent women and 56–65 year-olds has declined relative to their respective comparator groups.

**Fig. 9:**
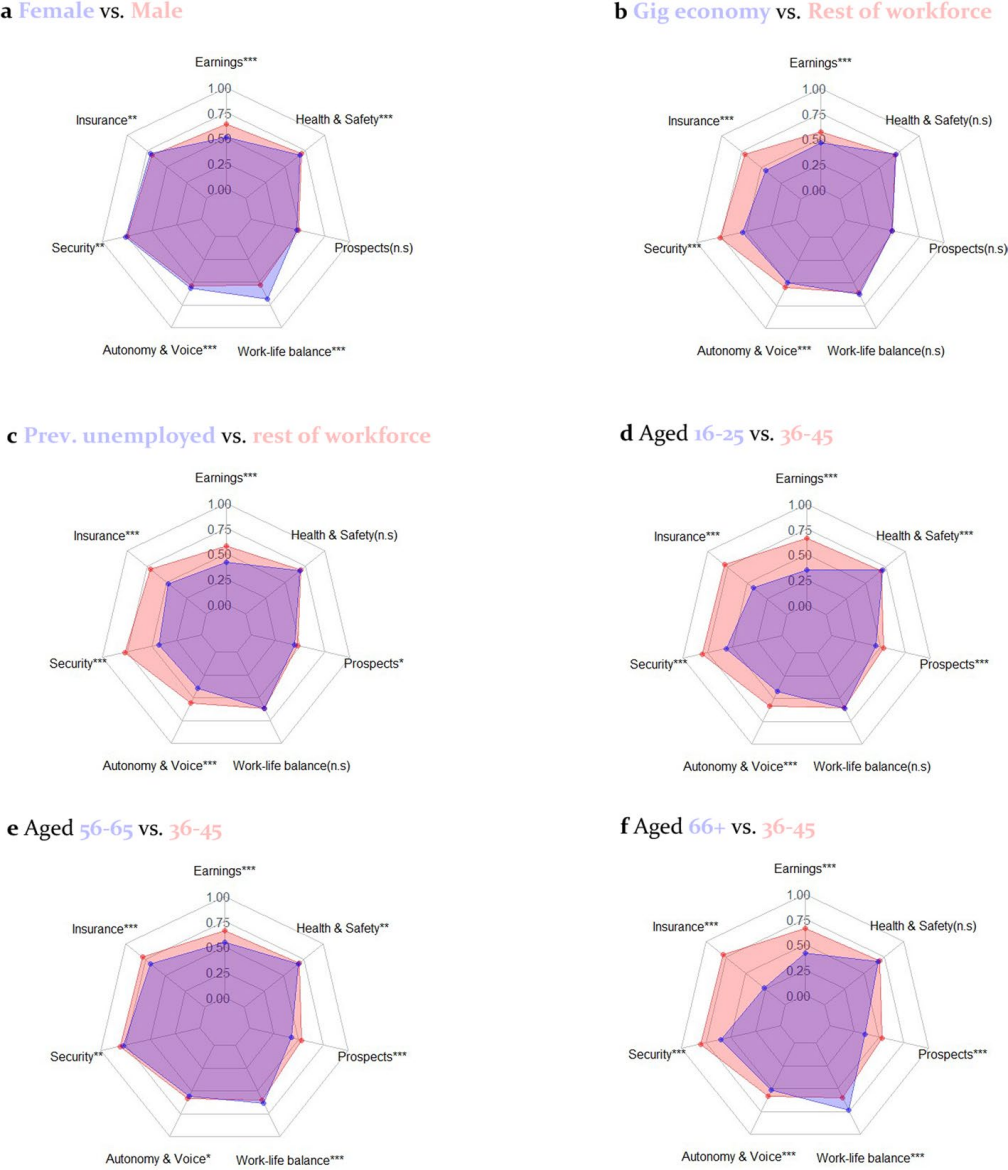

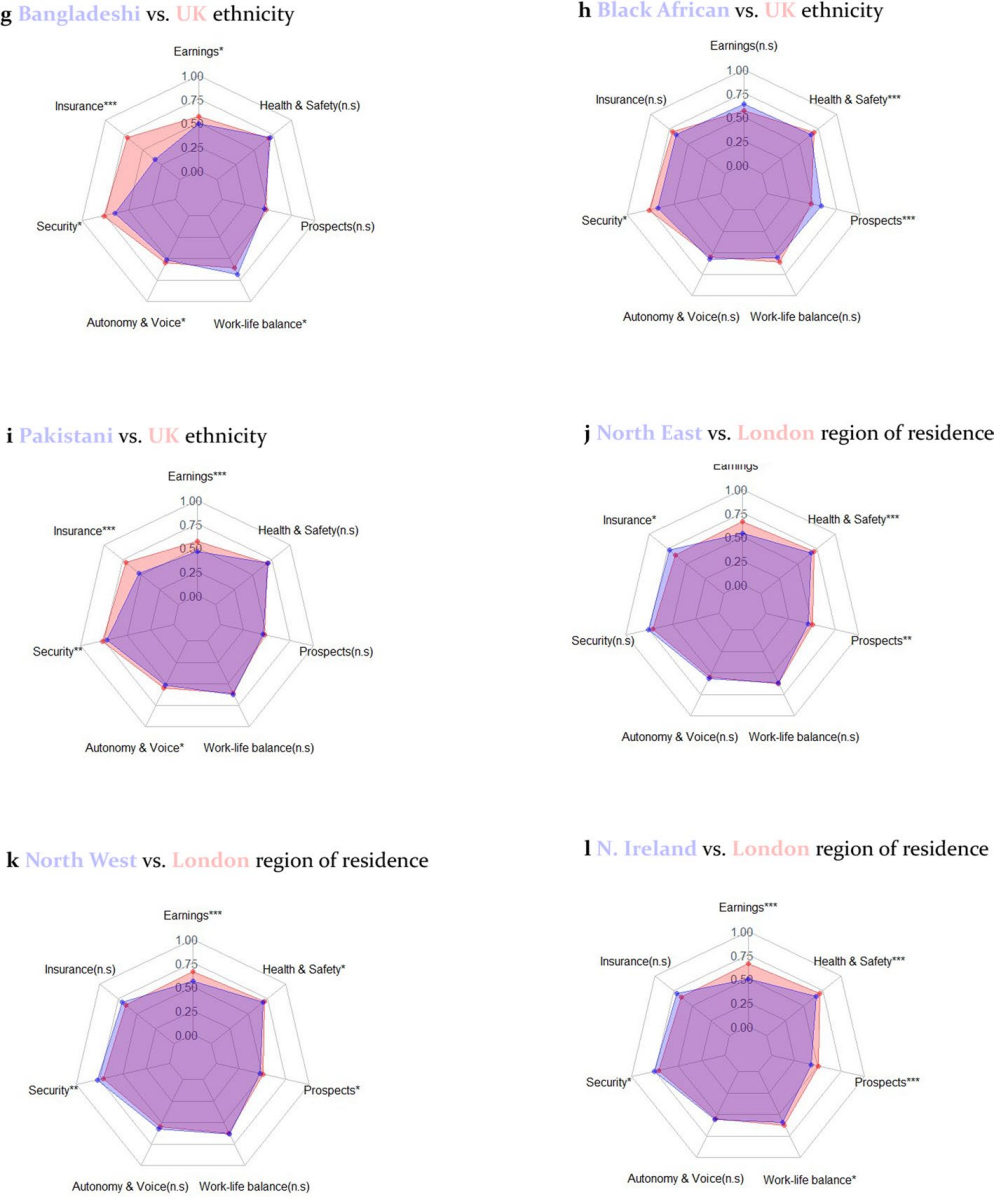
Radar plots of dimensional differences in QoW by pairs of sub-groups as at Wave 12 (2020–21). Asterisks represent whether the mean difference in QoW is statistically significant at the 0.05 (*), 0.01 (**) and 0.001(***) confidence level using a non-parametric independent samples test (Kruskhal-Wallis)

Finally, Fig. 9 illustrates where these inequalities continue to present themselves as at 2020–2021, showing radar plots of differences in each dimension of the QoW index for 12 sub-groups. Since the analysis in Fig. 9 is by dimension, these are not presented using different weighting approaches. Lower-scoring groups tend to consistently have lower earnings. This is particularly important in determining the poorer labour market position of women vs. men. There is also a tendency for lower-scoring groups to perform similarly or better on Work-Life Balance: they tend to work lower hours, which improves their score on Excessive Hours but often drops their scores in Earnings Sufficiency below the minimum income thresholds.

Beyond this, the inequalities in QoW manifest themselves rather differently for different sub-groups in society. The North East, Northern Ireland and North West score better than London on Insurance and Security, yet people of Bangladeshi and Pakistani ethnicity and the youngest and oldest age groups score considerably worse than comparator groups on both these measures.^8^ Notably, all age groups have considerably worse Prospects scores than the reference group of 36–45 year-olds—a potentially concerning finding for public policymaking, especially for the youngest workers. These findings illustrate the distinct roles which each of the dimensions of the index play in UK job quality. They also suggest that public policymakers need to tackle a broad range of issues to improve the labour market position of the most marginalised workers. This includes improving their earnings, but it should also involve tackling other vital non-pecuniary aspects of work where these inequalities manifest themselves.

### Relationships Between Indicators

**Fig. 10:**
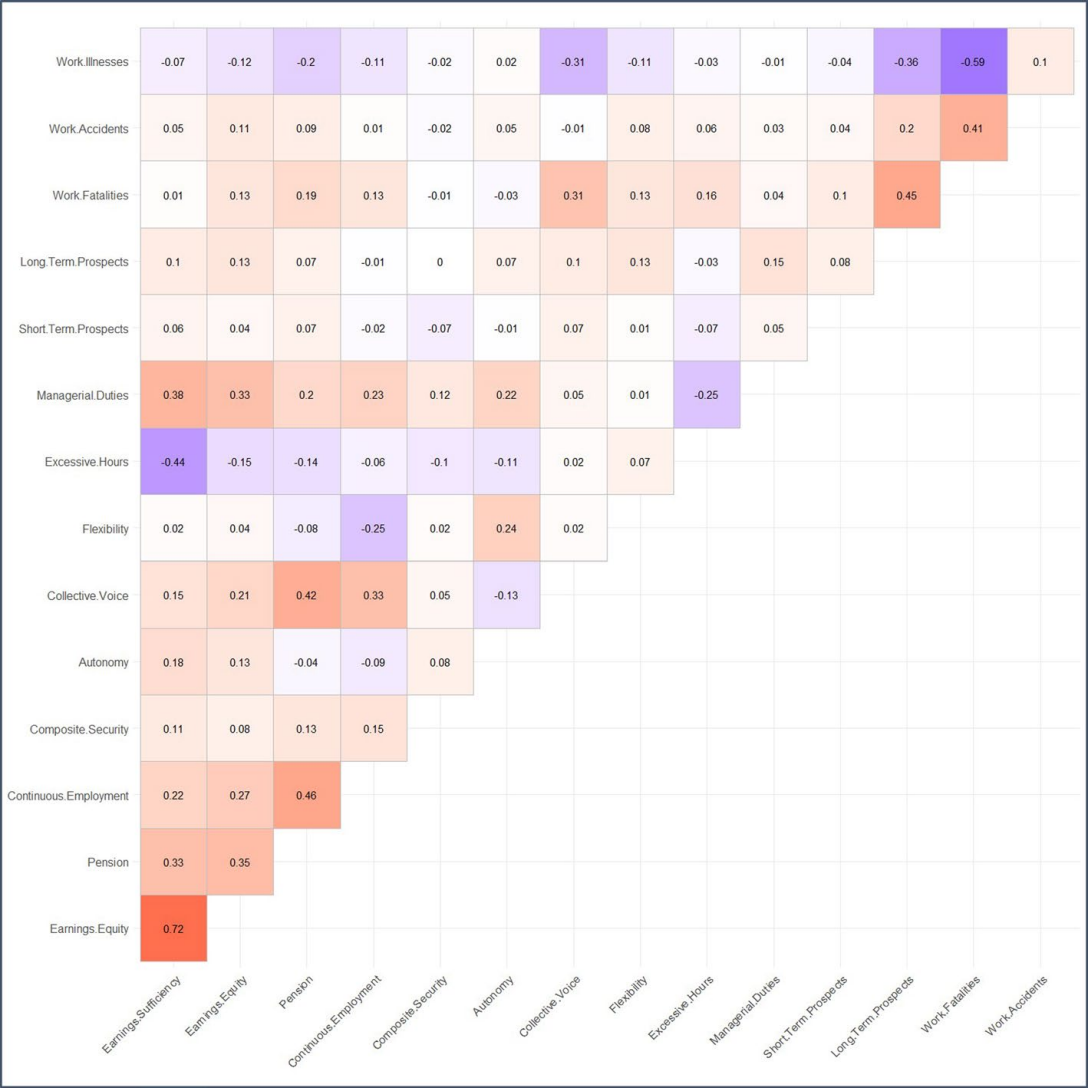
Correlation matrix of the standardised QoW indictor scores, using Spearman correlation coefficients. Pooled data from all waves of the QoW index

The previous subsection has given an insight into how inequalities in QoW manifest themselves differently across various groups in the UK, and are not uniform across these sub-groups. Figure 10 supplements this by presenting a correlation matrix of the standardised QoW indicator scores. Table 2 presents the results from a Principal Component Analysis (PCA) of this correlation matrix, showing the factor loadings of the first 8 principal components which together explain 90.2% of the variance in the data.

**Table 2:** Factor loadings of the first 8 principal components of the QoW index (explaining 90.2% of variance). Based on a Principal Component Analysis of the correlation matrix of standardised QoW index indicators, using Spearman correlation coefficients. Factor loadings > 0.3 marked italics and < − 0.3 marked bold italics. Pooled data from all waves of the QoW index

Indicator	Comp.1	Comp.2	Comp.3	Comp.4	Comp.5	Comp.6	Comp.7	Comp.8
Earnings Sufficiency	*0.419*	0.272	0.231	0.105	0.006	0.217	0.158	0.241
Earnings Equity	*0.382*	0.144	0.149	0.085	− 0.058	*0.396*	0.051	*0.393*
Pension	*0.395*	− 0.033	− 0.232	− 0.007	0.012	0.061	0.146	− ***0.346***
Continuous Employment	*0.338*	0.023	− ***0.398***	− 0.046	− 0.153	− 0.063	− 0.253	− 0.187
Composite Security	0.076	0.129	− 0.071	− ***0.503***	− 0.218	− ***0.553***	*0.433*	0.213
Autonomy	− 0.046	0.185	*0.435*	− 0.243	− 0.008	0.02	− 0.259	− ***0.518***
Collective Voice	0.297	− 0.241	− 0.26	− 0.156	0.245	0.199	0.143	− 0.197
Flexibility	− 0.147	− 0.109	*0.403*	− ***0.334***	0.214	*0.312*	*0.321*	− 0.145
Excessive Hours	− ***0.303***	− ***0.308***	− 0.242	− 0.141	− 0.074	*0.312*	− ***0.309***	0.276
Managerial Duties	0.252	0.221	0.201	0.009	− 0.075	− 0.194	− ***0.491***	− 0.079
Short Term Prospects	0.006	− 0.049	0.028	*0.455*	*0.669*	− ***0.341***	0.106	− 0.015
Long Term Prospects	0.124	− ***0.326***	*0.349*	0.087	− 0.101	− 0.289	− 0.201	0.3
Work Fatalities	0.165	− ***0.541***	0.145	0.079	− 0.167	− 0.073	0.025	− 0.09
Work Accidents	− 0.056	− 0.167	0.132	*0.469*	− ***0.549***	0.036	*0.332*	− 0.277
Work Illnesses	− 0.3	*0.458*	− 0.167	0.266	− 0.145	0.068	0.097	− 0.041
Proportion of variance	**30.8%**	**23.0%**	**13.9%**	**6.8%**	**6.0%**	**4.9%**	**3.7%**	**2.9%**

The correlation between many indicators is weaker than in many other multidimensional indices of wellbeing, and the first principal component explains quite a small proportion—30.8%—of the variance. This contrasts with many other wellbeing indices, such as the Human Development Index (cf. Noorbakhsh, 1998, p. 594). Factor loadings exceed ± 0.3—a common standard for PCA—for all but one indicator (Collective Voice), although it is very close to 0.3 in the first component. There is also a lack of large positive factor loadings for Work Fatalities and to a lesser extent Managerial Duties. The large number of negative factor loadings is again distinct from some other applications of PCA (Vyas & Kumaranayake, 2006, pp. 463–464). PCA in itself should not be seen as a validation of an index, and there are strong arguments against using it to inform weighting decisions (see Online Appendix A), yet it is noteworthy that the index nonetheless performs well according to this commonly used dimensionality reduction technique.

This negative relationship between some indicators is consistent with what some other job quality indices show. The European Job Quality Index for example shows a negative (although weak) association between their work-life balance and pay dimensions (Muñoz de Bustillo et al., 2011, p. 194) and even a negative relationship between some indicators within the same dimension, such as flexibility and hours worked (Muñoz de Bustillo et al., 2011, p. 188). This makes logical sense, since a fall in hours worked should all else held equal lead to a fall in earnings, and workers may access flexible work arrangements instead of reducing their hours to deal with work-family and family-work conflict. However, it should be emphasised that there is no inherent reason why the scores in these indicators would be negatively correlated, since the cut-offs used should not prohibit the achievement of good scores on all indicators simultaneously. To score best on Excessive Hours, a worker simply needs to work in line with the average number of working hours of 37 h a week (ONS, 2024d)—a commonly-stipulated contractual obligation in standard employment contracts. Yet it is striking that many workers working below this struggle to earn a decent wage and achieve a level of take-home pay above the Minimum Income Standards.

There is also a weak or negative correlation between Work Illnesses and most other indicators of the QoW index. The negative associations likely reflect its distribution across industries and occupations: for example, it tends to be higher in more heavily unionised industries such as human health and public administration (see Online Appendix D, Table D.3).

Finally, it should also be noted that although Earnings Sufficiency and Earnings Equity are strongly positively correlated and have consistently positive factor loadings in all but one component of the PCA, the strength of the correlation between these indicators declines over the course of the time series.^9^ This reflects the trends outlined in Sect. 4.1, and highlights the importance of measuring the twin aspects of earnings in precisely the way discussed in international literature.

## Conclusions

This paper has presented the first data from a comprehensive synthetic index of job quality in the UK, and analysed the effect of four different weighting approaches have on inequalities and changes in job quality in Britain.

The indicators and dimensions of the index have been identified based on a normative framework for measuring job quality using the Capability Approach, and there is therefore an emphasis on objective rather than subjective aspects of work in a way which is consistent with this strand of literature. Nevertheless, many of the indicators and dimensions of the index capture important aspects of job quality discussed in a broad spectrum of academic research (Gallie, 2003)—including job security, autonomy, workers’ voice, work-life balance and job prospects. This in itself provides a useful contribution to the debate of using job quality indices, shedding new light on important trends and inequalities in job quality within a single country context in an area of study where analysis of within-country inequalities in job quality is sometimes not possible.

The paper supplements this by making some innovations in the development of social indicators for job quality. Within the Earnings dimension, a crucial distinction is drawn between the position of workers in the *gross* wage distribution (Earnings Equity) and the sufficiency of their *net* earnings to meet societally-agreed minimum standards (Earnings Sufficiency). Indicators of pension enrolment and continuous employment are developed due to the specific role these play in the UK context. Finally, four important indicators on long-term job prospects and health and safety are introduced into the index using data from external sources, bringing these indicators into Understanding Society for the first time to allow us to analyse their relationships with other job quality indicators.

The main contribution of the paper, however, lies in its presentation of findings using four different weighting approaches. The default weighting approach used in the UK QoW Index is informed by the Alkire-Foster method, but alternative index scores using three additional weighting approaches—hedonic, frequency-based and data-driven—are introduced in order to test the sensitivity of findings to different views about weighting. The paper has found that the UK has seen an improvement in job quality in precisely the two areas which have been the focus of public policymakers: improving hourly wages for those at the bottom of the distribution; and improving pension coverage for employees. This has led to a reduction in inequality in job quality for some sub-groups, notably the youngest workers, and between regions. However, despite these improvements, there has also been a polarisation in job quality between self-employed workers vs. employees, and to a lesser extent women vs. men. Inequalities in job quality between many sub-groups of workers also show no signs of falling. Crucially, these broad findings are consistent across the four weighting approaches used in this study. Further, with the exception of hedonic weighting, all weighting approaches broadly agree on the *relative* position of different sub-groups, if not always the *size* of the difference between these sub-groups.

Even within the Earnings and Pensions dimensions, the improvement for UK workers has not been uniform. Self-employed workers have not benefitted from the drive to increase workers’ pension enrolment, and the sufficiency of their earnings has declined despite the rise in gross hourly wages. This suggests a more broad-based set of interventions is needed to improve UK job quality: addressing the factors keeping *net* earnings low, particularly hours worked and pay deductions; including the informal economy in labour market interventions such as pensions; and taking steps to improve vital non-pecuniary aspects of work which have deteriorated in the past decade, such as Short-Term Prospects and Composite Security.

There are nonetheless some limitations to this study. The changes outlined above have occurred in the context of significant changes in the population in the QoW index due to the rise of the employment rate, yet in common with other job quality indices I do not capture the experience of individuals who are not in paid employment. The dataset used also does not yet fully capture the post-lockdown years, but the index developed will be able to supply consistent data to enable such analysis in future. Nor is any claim being made to give a comprehensive picture of all possible alternative weighting methods—with a particular neglect of any weights which vary at an individual or group-level, such as peoples’ self-assessments of the weight each indicator should be given.

Going forward, I suggest that future research could build on this work by further investigating important within-country inequalities in job quality, and operationalising a broad spectrum of alternative weighting approaches when presenting results. This, in turn, may help secure greater public policymaker interest in “good work”—by building consensus about how to measure the concept; and strengthening the forcefulness of findings, by showing they are robust to different weighting methods. In time, this could pave the way towards more regular, more detailed, and more widely-discussed published job quality statistics.

## Supplementary Information

Below is the link to the electronic supplementary material.

Supplementary file1 (PDF 899 KB)

## Data Availability

This analysis was conducted using safeguarded data from Understanding Society and, for one dimension, the Labour Force Survey. These datasets are held by the UK Data Service (UKDS) so users looking to replicate the analysis will need to access the data via them and agree to the UKDS and the respective data owners' conditions for data access. To facilitate access and support other research applications, the syntax used to generate the QoW index (using 'R') is available open access (CC BY SA 4.0), and has been deposited with the UKDS on ReShare (SN 857836). It can be accessed at: 10.5255/UKDA-SN-857836.

## Footnotes

1 The fuzzy set theory literature tends to define these three types of indicator differently using the labels “dichotomous”, “polytomous” and “continuous”, respectively (see Deutsch and Silber, 2005). I retain their underlying definitions but rename the first two as the more intuitive “binary” and “categorical.”.

2 Note that ‘worst’ and ‘best’ do not necessarily reflect the lowest or highest raw values, respectively, since this depends on the nature of the indicator: e.g. for an indicator of occupational health and safety, a *higher* incidence reflects *worse* job quality.

3 This is similar to the formula presented in Deutsch and Silber (2005, p. 148). Only the middle line is completely necessary, but the minimum and maximum scores are specified separately here to aid in transparency and understanding.

4 Wave 2 is excluded due to the lack of data on the Continuous Employment indicator for this wave.

5 For a fuller discussion, refer to Appendix F.1 and Stephens (2023b, 2023c).

6 This feature of UK labour law looks set to change in future, as the newly-elected Labour Government has set out plans to give UK workers full protection against unfair dismissal from day one of employment in a new Employment Rights Bill (Prime Minister’s Office, 2024). This will likely require revisions to the Continuous Employment indicator in future, but it is used in this study to reflect the real-life job security of those workers when surveyed, given the legal context at that point.

7 This consists of a small number (unweighted n < 500) of respondents who either use zero hours’ contracts (jbfxuse9 = 1) or who report working in a gig economy job (any of gelist1-5 = 1). The two categories are almost completely mutually exclusive. The required variables were only introduced from Wave 12, so there is no time series data for this comparison group.

8 As a caveat, the considerably lower scores of the oldest (aged 66 +) workers may of course reflect their *better* position in the labour market: many of these workers may have already achieved a good workplace pension, and have freely chosen to work during retirement – either to supplement their retirement income, or to engage in some meaningful work activity. Despite this, I would caution against excluding older age groups from job quality indices, because these scores may also reflect the precise opposite: they could just as likely be older workers stuck in poor-quality jobs, with no prospect of a decent retirement. It is beyond the scope of this paper to investigate this more closely; further research is needed to understand the wellbeing of this sub-group of workers in greater detail.

9 Supplementary analysis, not presented here.
