# Supplementary material for: What has Happened to Job Quality in Britain? The Effect of Different Weighting Methods on Labour Market Inequalities and Changes Using a UK Quality of Work (QoW) Index, 2012–2021
Source: Soc Indic Res. 2025 Feb 11;177(2):833–61. doi: 10.1007/s11205-025-03542-9 (PMC11993496; doi:10.1007/s11205-025-03542-9)
Supplement: Supplementary file 1 — Supplementary file1 (PDF 899 KB) [file 11205_2025_3542_MOESM1_ESM.pdf]

Online methodological appendix for the UK Quality of Work (QoW) Index

Appendix A – Alternative weighting methods for the QoW index 2
Appendix B – Missing values analysis of Understanding Society data 13
Appendix C – Multiple imputation method for missing data 19
Appendix D – Health and Safety dimension 24
Appendix E – Long-Term Prospects indicator 37
Appendix F – Further discussion of QoW indicators and dimensions 44
References 53

Appendix A – Alternative weighting methods for the QoW index

This study operationalises three different normative processes for deriving an alternative set of weights for each indicator in the QoW index: hedonic weighting, frequency-based weighting, and data-driven weighting. The process for creating weights using these three approaches is set out in the next three subsections of this appendix. In each of these three cases, a set of alternative weights for each indicator of the QoW index is arrived at. After these alternative weights are set out, the effect of these different weights on both (a) time series trends and (b) sub-group differences in QoW index scores are then compared, vis-à-vis equal weighting and the alternative weighting approaches.

Before proceeding, some technical aspects and common misconceptions of weighting should be addressed. As is highlighted in some studies (e.g. see Leschke and Watt, 2014, p. 4), some debates about weighting can be mitigated by the way the data is presented. The emphasis of most analysis in this paper is on how dimensional scores vary by sub-group, and the different correlations between indicators and dimensions of the QoW index. Such analysis is not sensitive to the weight chosen: so long as there is agreement on the appropriateness and calculation process for each indicator and dimension (a big assumption, but one not related to the weighting), then there should be broad agreement on the conclusions. The weighting instead affects how these are then *aggregated*. This impacts three key conclusions in multidimensional analysis:

- Time series analysis of trends in index scores, both for the population as a whole and for sub-groups;
- Any analysis, using regression or other techniques, comparing the effect of various independent variables on index scores;
- In studies of poverty or deprivation, the proportion of deprived people in an index, where the relative weight of an indicator or dimension has a role in deciding this deprivation.

The third issue is less relevant to this paper, since I have avoided making a statement about deprivation or generating censored headcount ratios. The first two are very pertinent, particularly with reference to the findings of any differences in, and polarisation of, job quality, both over time and between sub-groups.

Some limitations to the analysis in the succeeding sections should be noted. None of the weights proposed in this analysis vary by individual in the way advanced in some other weighting proposals: decisions are made to inform the weighting of indicators for the whole population. Nor are the individual indicators or cut-offs in the index changed (although some indicators are weighted zero). Finally, for simplicity, alternative weights are created at the indicator-level without the indicators being aggregated into dimensions.

A.1. Hedonic weighting

One strand of hedonic or utilitarian research argues that subjective life satisfaction and/or job satisfaction should play a central role in our assessment of wellbeing (Kahneman, 1999; Layard, 2011). A further strand of research has emerged arguing that, even if we disagree on the role subjective factors should play in wellbeing, peoples' subjective self-assessments could nonetheless play a role in the relative weight we assign different dimensions of wellbeing. This approach is strongly advocated by Schokkaert (Schokkaert, 2007; Schokkaert et al., 2009), who argues that the relative weights of different dimensions could be derived simply by regressing the effect of these dimensions on life satisfaction (see also Decancq and Lugo, 2013, pp. 26–27). He

also argues that this approach could give us an indication of the value people would assign these dimensions *if* they were asked in a participatory exercise.

In order to operationalise this proposal, it is essential that a range of observable and unobservable characteristics are controlled for – such as individual idiosyncrasies like peoples' own perspectives on life, time, and other factors which affect the relationship between indicators/dimensions and subjective life satisfaction. Schokkaert (2007, pp. 423–428) highlights religion as one such control, but he stresses that with panel data even unobservable idiosyncrasies could be controlled for in a fixed effects model. He proposes that broadly-framed questions on general satisfaction – "On the whole, are you very satisfied, fairly satisfied, not very satisfied or not at all satisfied with the life you lead?" – as best suited to this (Schokkaert, 2007, p. 417), in contrast to the more fleeting hedonic measures proposed in other happiness literature (e.g. see Kahneman et al., 2004, p. 430).

There are normative reasons to reject this line of argument. Philosophies such as the Capability Approach (Nussbaum, 2011; Sen, 1999) emphasise the role of more objective "beings and doings" (Functionings) in determining peoples' wellbeing, rejecting by definition subjective factors as a sole determinant of quality of life. A great deal of job quality literature has attempted to operationalise this (see for example Green, 2009; Sehnbruch, 2004), of which this present paper is a part. Schokkaert's (2007) proposal also cannot be equated with a genuine participatory exercise on wellbeing measurement (cf. (Burchardt and Vizard, 2011)). Nevertheless, this method can provide a useful test of alternative weighting decisions for any job quality index, allowing us to explore the effect that a more hedonic perspective would have on the weighting decisions made in the index. If the conclusions drawn are broadly similar even with a hedonic weighting approach, then this would provide the basis for at least some agreement between competing philosophies of wellbeing as to the worth of the QoW index.

To explore the weights which could be used in a hedonic measure of job quality, this paper regresses the effect of each indicator in the QoW index on two measures of life satisfaction, both of which closely resemble Schokkaert's (2007, p. 417) preferred measures of wellbeing. Both wellbeing questions are asked on the same (1-7) scale, and all the variables are all standardised. This means that the coefficients of both models can be directly compared:[1]

- Job satisfaction: "On a scale of 1 to 7, where 1 means 'completely dissatisfied and 7 means 'completely satisfied', how satisfied or dissatisfied are you with your present job overall?'
- Life satisfaction: Asked as part of five questions on more general life satisfaction, with people asked to "choose the number which you feel best describes how dissatisfied or satisfied you are with the following aspects of your current situation" about "how you feel about your life." They are also asked to place this on the same 1-7 scale as for job satisfaction from 1 (completely satisfied) to 4 (neither satisfied nor dissatisfied) through to 7 (completely satisfied).

Simply regressing these against the indicators using cross-sectional data, for any given time period, would not be informative about the effect these characteristics have on subjective satisfaction. Without introducing controls, any differences observed could reflect a difference in an uncontrolled variable: simple cross-sectional differences in job or life satisfaction between jobs with different characteristics clearly reflects differences in the people doing those jobs, rather

[1] Standardisation is necessary because otherwise, the different effect sizes (coefficients) could be determined by the nature of the indicators and thus it wouldn't be possible to use them to inform a weighting decision. Standardisation is done by dividing the values by their standard deviations, using the 'standardize' (sic) function in R's jtools package. For a discussion of standardisation using this package, see Gelman (2008).

than the jobs themselves. It is also likely that these differences cannot be controlled using simply observable characteristics: as Schokkaert (2007) highlights, individuals will have unobservable idiosyncrasies and personality characteristics which affect the way job characteristics impact their subjective satisfaction.

I take advantage of the panel construction of Understanding Society to account for such unobserved factors. I create a balanced panel of people who are represented in all five waves used in the survey.[2] I then create a linear first-difference individual fixed effects model to explore the effect of changes (Δ) in indicator scores on job and life satisfaction over time, of the following form:

$$\Delta Satisfaction^{it} = \beta_0 + \Delta\beta_1\ Indicator^{it} + \Delta\beta_2\ Age^{it} + \Delta\beta_3\ Region^{it} + + \Delta\beta_4\ Children^{it} + \\ + \Delta\beta_5\ WorklessAdults^{it} + + \Delta\beta_6\ CaringResponsibilities^{it} + \Delta u^{it}$$

$\Delta Satisfaction^{it}$ is the change in job satisfaction or life satisfaction for individual i at time t minus their satisfaction at time t-1, in any given wave, i.e.: $\Delta Satisfaction^{it} = \Delta Satisfaction^{it} - Satisfaction^{i,t-1}$. After accounting for individual and time fixed effects, this change is held to be explained by six observable characteristics – the change in QoW indicator scores for individual i at time t ($\Delta\beta_1\ Indicator^{it}$); and five controls for things which could change over time and be correlated with a change in job characteristics: age of respondent ($\Delta\beta_2\ Age^{it}$), region of residence (($\Delta\beta_3\ Region^{it}$), number of dependent children in the individual's household ($\Delta\beta_4\ Children^{it}$), number of workless adults in the household ($\Delta\beta_5\ WorklessAdults^{it}$) and whether the respondent has within-household caring responsibilities ($\Delta\beta_6\ CaringResponsibilities^{it}$) – together with an error term of other unobserved characteristics which change over time (Δu^{it}). The effect that these indicators have on changes in satisfaction gives us a sense of the *causal* impact of a change in indicator scores on a change in job or life satisfaction, and thus the relative weight that should be assigned to these indicators in a hedonic index of job quality. It should be noted, however, that there is no reason to assume that these models account for the role of adaptation in satisfaction. For example, where a decline in one's labour market position leads to adaptation in the form of higher job or life satisfaction, this would be captured in the model.

Table A.1 presents the regression outputs for four sets of regressions: two sets with life satisfaction as the outcome variable, the first (1) a set of 15 separate regressions with each of the QoW indicators separately and the second (2) a single regression with all QOW indicators included; and two sets of regressions with job satisfaction as the outcome variable, again done on all QoW indicators separately (3) and then together (4). The use of separate regressions has been done to test the risk of multicollinearity and double measurement where the QoW indicators are highly correlated, which would make some of the estimates insignificant. The indicator scores have been standardised to allow for consistent comparison of the coefficients.

[2] In common with analysis of this type, the restriction to only people who responded in each of these five waves makes the panel unrepresentative of the UK population. This justifies the decision of a fixed effects analysis. A Hausman test, not presented here, reinforces this, and found significant differences between a fixed effects and a random effects model using the data.

Table A.1. Linear first-difference one-way (individual) fixed effects regressions of effect of changes in standardised QoW indicator scores on life satisfaction and job satisfaction. Asterisks denote significance: (.) = 0.1, * = 0.05, ** = 0.01, *** = 0.001.

	Life Satisfaction		Job Satisfaction	
	Excl. other indicators (1)	Incl. other indicators (2)	Excl. other indicators (3)	Incl. other indicators (4)
**Earnings Sufficiency**	**0.029**** (0.011)	**0.023(.)** (0.012)	0.00 (0.011)	0.01 (0.018)
**Earnings Equity**	**0.035***** (0.009)	**0.026**** (0.009)	0.007 (0.009)	0.011 (0.021)
**Pension**	0.003 (0.008)	-0.001 (0.008)	0.007 (0.008)	0.015 (0.013)
**Continuous Employment**	-0.006 (0.005)	-0.009 (0.005)	**-0.094***** (0.006)	**-0.249***** (0.013)
**Composite Security**	**0.049***** (0.009)	**0.049***** (0.009)	**0.137***** (0.009)	**0.181***** (0.012)
**Autonomy**	**0.04***** (0.007)	**0.039***** (0.007)	**0.119***** (0.007)	**0.232***** (0.014)
**Collective Voice**	0.009 (0.009)	0.006 (0.009)	0.014 (0.01)	0.019 (0.012)
**Employee Flexibility**	**0.016*** (0.007)	**0.011(.)** (0.007)	**0.039***** (0.007)	**0.057***** (0.012)
**Excessive Hours**	**0.026***** (0.008)	**0.027***** (0.008)	**0.019*** (0.008)	**0.033*** (0.015)
**Managerial Duties**	-0.01 (0.01)	-0.014 (0.01)	**-0.039***** (0.011)	**-0.047***** (0.014)
**Short-term Prospects**	-0.002 (0.006)	0.00 (0.006)	**-0.06***** (0.006)	**-0.105***** (0.011)
**Long-term Prospects**	-0.009 (0.013)	-0.009 (0.013)	**-0.039**** (0.014)	**-0.104*** (0.05)
**Work Fatalities**	0.018 (0.016)	0.01 (0.017)	**-0.041**** (0.016)	**-0.308*** (0.132)
**Work Accidents**	**0.023(.)** (0.013)	0.021 (0.014)	-0.008 (0.013)	0.012 (0.061)
**Work Illnesses**	-0.012 (0.012)	-0.011 (0.012)	**-0.023(.)** (0.012)	-0.054 (0.046)
Individuals	6,982		6,985	
Observations	24,504		25,118	

Table A.2. Summary of hedonic weights for each indicator of the QoW index.

Indicator	Significant life satisfaction estimates (*2)		Significant job satisfaction estimates		Sum of significant & consistent estimates	Weight
	(1)	(2)	(3)	(4)		
Earnings Sufficiency	0.058	0.046	-	-	0.104	**6.49%**
Earnings Equity	0.07	0.052	-	-	0.122	**7.61%**
Pension	-	-	-	-	-	**0%**
Continuous Employment	-	-	-0.094	-0.249	-	**0%**
Composite Security	0.098	0.098	0.137	0.181	0.514	**32.06%**
Autonomy	0.08	0.078	0.119	0.232	0.509	**31.75%**
Collective Voice	-	-	-	-	-	**0%**
Employee Flexibility	0.032	0.022	0.039	0.057	0.15	**9.36%**
Excessive Hours	0.052	0.054	0.019	0.033	0.158	**9.86%**
Managerial Duties	-	-	-0.039	-0.047	-	**0%**
Short-term Prospects	-	-	-0.06	-0.105	-	**0%**
Long-term Prospects	-	-	-0.039	-0.104	-	**0%**
Work Fatalities	-	-	-0.041	-0.308	-	**0%**
Work Accidents	0.046	-	-	-	0.046	**2.87%**
Work Illnesses	-	-	-0.023	-	-	**0%**

Positive estimates mean that an improvement in an indicator between waves is associated with an increase in subjective life or job satisfaction. The data suggests significant positive impacts of Composite Security, Autonomy, Flexibility and Excessive Hours, with particularly large coefficients for the job satisfaction regressions. The Earnings indicators have a significant positive effect on life satisfaction but not on job satisfaction, whist Work Accidents has a small positive effect in one of the life satisfaction models. All of the controls (not presented here) except for the presence of non-working adults in the household have a significant effect for job and/or life satisfaction. There are also some significant negative associations, where an *improvement* in an indicator is associated with *lower* satisfaction; these are discussed below.

A few complexities with applying these to an index of job quality need to be navigated. The decisions made on these are set out below:

- The effects need to be aggregated together to create a weight. I apply a two-thirds weight to life satisfaction regressions, reflecting its greater importance in determining subjective wellbeing and its closer approximation to the kind of measure proposed in Schokkaert (2007). This effectively doubles the estimates of the two sets of life satisfaction measures.
- Coefficients should only be incorporated if they are significantly related to life- and/or job satisfaction. I include estimates from either model with a higher than 10% significance (p = <0.1; denoted by a '(.)' in the tables). If the effect is only significant in one model (as is the case with Earnings), then only the effect from this model will be carried over into the weighting. This is a slightly more relaxed significance threshold than conventionally used, but is I feel justified by the subject matter since it is still markedly *more likely than not* that the indicator impacts the outcome variables.

- Some of the coefficients for job satisfaction (though not life satisfaction) are both significant and negative, meaning that an *improvement* in the indicator is associated with a *decline* in job satisfaction. This is especially the case for Continuous Employment and for the indicators in the Prospects and Health and Safety dimensions. This finding is consistent with other literature which shows a converse relationship between some indicators of job quality and subjective job satisfaction (see for example Léné, 2019; Muñoz de Bustillo and Fernández Macías, 2005). This poses a challenge: should these negative effects be included in the index by effectively inverting the indicator scores, or should they simply be excluded? Since they are not significant for the more important measure (life satisfaction), a decision is made not to include them in the index rather than inverting the indicator scores.

Table A.2 contains the resulting weights for a hedonic weighting approach of the QoW index. As discussed above, these are simply the sum of the effect sizes where these are significant and consistent. This leaves 7 indicators in the QoW index, with the remaining 8 indicators removed. Note that because there is no Employee Flexibility indicator for self-employed workers, in line with what is effectively the method for the equal weighting approach, the additional weight for this is allocated to the Excessive Hours indicator.[3] Finally, the hedonic indicator scores are adjusted along a 0-8 scale, so the scores can be directly compared with the QoW index scores.

A.2. Frequency-based weighting

Another body of literature, drawn from research of multidimensional poverty, argues that the weights of an indicator should be determined by the inverted proportion of people deprived in that indicator, relative to the other indicators in an index. There is an intuitive logic to this approach: people will feel more deprived in an indicator where this deprivation is shared by fewer people in a society, and less deprived where it is a relatively common deprivation. The method for weighting indicators in this way is described in detail by Deutsch and Silber (2005), which particularly draws from Cheli and Lemmi (1995) and Cerioli and Zani (1990) (see also Decancq and Lugo, 2013, pp. 19–20).

This paper draws from these approaches to develop an alternative frequency-based weighting method for the QoW index. Again, as with hedonic weighting, there are normative reasons to reject this weighting method. Whilst many poverty lines are drawn based on the extent of deprivation in a society, one could argue that there is nothing in the frequency of deprivation that inherently impacts the effect of an indicator on wellbeing: a low frequency of aggregate achievement in a society doesn't necessarily translate into low Functioning achievement for the individual worker. One could also challenge whether a deprivation-based threshold is suitable for a *job quality* index: as briefly discussed in the paper, job quality and deprivation are different things, with the former more amenable to being viewed along a spectrum of achievement levels. The cut-offs for the QoW index are used to determine achievement along this spectrum, and should not necessarily be equated with deprivation thresholds. Much as with the hedonic weighting, however, these objections confuse the purpose of this weighting exercise, which is chiefly to explore the sensitivity of this paper's findings to different reasonable weighting decisions.

[3] This is of course a contentious compromise, but one that is brought about by the lack of available data. By grouping these two indicators into a conceptually similar dimension, the additional weight of Employee Flexibility for self-employed workers is entirely allocated to Excessive Hours. This method is therefore retained for the all the weighting approaches presented here: where a method suggests a particular weight should be applied for Employee Flexibility, the weight for Excessive Hours for self-employed workers is the Employee Flexibility weight plus the Excessive Hours weight.

Notwithstanding these caveats, to implement frequency-based weighting, I start by calculating an approximation of the fuzzy proportion of deprived individuals for each indicator in the QoW index. This is done by taking 1 minus the mean score (m) in any given indicator (j) as at Wave 4 (2012-13): 1 - m_{j4}. The choice of Wave 4 is deliberate: given that the scores, and thus deprivation levels, do change over time on some indicators, it would be misleading to use a pooled mean from all waves or indeed a mean from any later wave. Selecting the earliest wave of analysis allows us to assess whether any improvement has been in the indicators which an advocate of frequency-based weighting would most want public policymakers to focus: namely, those indicators which the lowest proportion of people score low on at the start of the time series.

Weights then need to be applied to every indicator in a way which assigns a greater weight to indicators with lower fuzzy proportions of deprived individuals. In line with the method used in the Totally Fuzzy Approach (see in particular Deutsch and Silber, 2005, p. 150), this is calculated as an inverse of the aggregate level of deprivation across all indicators. As they propose, the normalised log (ln) is used so as to smooth the variation between scores. For example, the weight for indicator j, W_j, is calculated as:

$$W_j = \frac{\ln \frac{1}{1 - m_{j4}}}{\sum_{j=1\ to\ k} \ln \frac{1}{1 - m_{j4}}}$$

Table A.3 sets out the weights, in percentage terms, for each indicator using this frequency-based weighting approach (column 4). For transparency, these are set alongside the weights were the normalised logs not used (column 3). It can be observed that the highest weight is allocated to Work Fatalities since only a very small proportion of workers operate in industries with a high rate of fatalities, although log normalisation has the effect of markedly reducing the weight from what it would have been. There is a closer alignment of the weights of the other indicators, ranging from Managerial Duties (5.1%) to Composite Security (8.29%).

Table A.3. Summary of frequency-based weights for each indicator of the QoW index.

Indicator	Mean indicator score as at Wave 4	Fuzzy % of "deprived"	% weight (non-ln)	% weight (ln)
Earnings Sufficiency	0.473	52.7%	3.06%	**5.62%**
Earnings Equity	0.608	39.2%	4.12%	**6.32%**
Pension	0.483	51.7%	3.12%	**5.67%**
Continuous Employment	0.710	29.0%	5.57%	**7.02%**
Composite Security	0.832	16.8%	9.62%	**8.29%**
Autonomy	0.715	28.5%	5.67%	**7.06%**
Collective Voice	0.424	57.6%	2.80%	**5.42%**
Employee Flexibility	0.527	47.3%	3.41%	**5.88%**
Excessive Hours	0.655	34.5%	4.68%	**6.61%**
Managerial Duties	0.338	66.2%	2.44%	**5.10%**
Short-term Prospects	0.342	65.8%	2.45%	**5.11%**
Long-term Prospects	0.822	17.8%	9.06%	**8.15%**
Work Fatalities	0.955	4.5%	36.14%	**11.36%**
Work Accidents	0.600	40.0%	4.04%	**6.27%**
Work Illnesses	0.578	42.2%	3.82%	**6.14%**

A.3. Data-driven weighting

Finally, data-driven weighting is an increasingly common method used in multidimensional indices of wellbeing. This is most often done using Principal Component Analysis (PCA) (e.g. see (Cascales Mira, 2021; McGillivray, 2005; Noorbakhsh, 1998). PCA is a data reduction technique which transforms the indicators of an index into an equivalent number of principal components: linear composites of the indicators, uncorrelated with each other. Each of these components explains an amount of variance in the data in descending order, with the first principal component explaining the most variance. Each contains a linear weighted combination of the indicators, called factor loadings: a higher factor loading signifies a greater presence of the indicator in that principal component. The commonly-stated purpose for PCA has been summarised by Decancq and Lugo (2013, pp. 20–21):

> *"The use of principal component analysis is often motivated by a concern for the so-called problem of double-counting. In many empirical applications, the indicators of wellbeing are found to be strongly correlated and capturing the same latent dimension."*

This can be useful, since sometimes indices are not transparent about the extent to which the different indicators or dimensions are correlated. Weights can be assigned using PCA by either taking each indicator's factor loadings either the first principal component (see for example Greco, 2018, p. 464); or an average of the factor loadings weighted by the contribution of each principal component to the variance. There are also more sophisticated versions of PCA (e.g. see Boelhouwer, 2002) or factor analysis / latent variable analysis (for a discussion, see Krishnakumar and Nagar, 2008), but I consign myself to a PCA-based weighting method for this study because PCA is an efficient, easy-to-understand and widely used method adopted widely in existing research.

As with the other weighting approaches, strong normative reasons against weighting using PCA have received wide discussion in the literature. If anything, the case against PCA is stronger, since the normative grounds for adopting PCA-based weighting are often not clear. PCA will penalise indicators which do not vary across cases, and will not work as well when indicators are uncorrelated with each other (Vyas and Kumaranayake, 2006, p. 461), yet there is no logical reason why either of these factors should have any bearing on the effect an indicator has on wellbeing. An indicator which varies little across the population might still be justified in an index, since – as proponents of frequency-based weighting might argue – a small but important proportion of the population may still be deprived in that indicator. A related issue arises for the indicators in the QoW index which assign automatic scores for self-employed workers or for whom the self-employed are excluded: Continuous Employment, Collective Voice and Employee Flexibility. Despite the justification of the approaches for these indicators, this will reduce the variance in these indicators in any PCA. Nor is "double-counting" necessarily an issue in multidimensional indices: there can be strong reasons for including two indicators where they each have a marked effect on peoples' wellbeing. Again, however, the use of PCA in this study allows us to test the difference in weights if a popular data-driven weighting method were used for the QoW index. This is similar to the way it is used by Greco (2018). It also helps provide transparency about where any double counting occurs.

Pursuant to this, I carry out a PCA on the standardised[4] indicators of the QoW index. The PCA is conducted on the correlation matrix of the data, and not the covariance matrix. For ease of

[4] In line with the method used in other applications of PCA, these have been standardised so that each indicator has a mean of zero and a standard deviation of 1.

reference, Table A.4 copies the factor loadings of the first 8 principal components, which together explain just over 90% of the variance, from the paper. There are more negative factor loadings than is common in PCA, and the implications of this are discussed below.

The rightmost column of table A.5 shows the weights of each indicator in the QoW index using a data-driven weighting approach. I create these by taking a weighted average of these 8 principal components. This is because the use of only the first principal component has been criticised for cases, as here, where a relatively low proportion of the variance is explained by the first component (see Somarriba and Pena, 2009, p. 117). The relatively large number of negative factor loadings poses a challenge for the weighting. Consistent with the approach used in other literature (Greco, 2018, p. 466; Vyas and Kumaranayake, 2006, pp. 463–464), negative factor loadings are excluded and thus not used to determine weightings. This yields weights which are different from equal weighting, although not dramatically so for most indicators save for the Earnings indicators, Short Term Prospects and Excessive Hours. Indeed, the combined weight for the Earnings indicators – 26.9% - is very close to its 25% weight in the QoW index. There is an argument for instead inverting the negative factor loadings rather than excluding them, since PCA here is not being used for dimensionality reduction and it is an intended feature of the index for some indicators to be negatively loaded. For transparency, the weights were this latter approach taken are also included in table A.5; these would give even closer weights for most indicators.

Table A.4. Weights and factor loadings of the top 8 (>90% variance explained) principal components of the UK Quality of Work index. PCA conducted on the correlation matrix of standardised QoW index indicators, using Spearman correlation coefficients. Factor loadings > 0.3 marked green and < -0.3 marked red.

	Comp.1	Comp.2	Comp.3	Comp.4	Comp.5	Comp.6	Comp.7	Comp.8	% Weight (incl. negatives)	% Weight (excl. negatives)
Earnings Sufficiency	0.419	0.272	0.231	0.105	0.006	0.217	0.158	0.241	8.6%	**14.7%**
Earnings Equity	0.382	0.144	0.149	0.085	-0.058	0.396	0.051	0.393	7.2%	**12.1%**
Pension	0.395	-0.033	-0.232	-0.007	0.012	0.061	0.146	-0.346	6.1%	**7.5%**
Continuous Employment	0.338	0.023	-0.398	-0.046	-0.153	-0.063	-0.253	-0.187	6.5%	**6.3%**
Composite Security	0.076	0.129	-0.071	-0.503	-0.218	-0.553	0.433	0.213	5.4%	**4.3%**
Autonomy	-0.046	0.185	0.435	-0.243	-0.008	0.02	-0.259	-0.518	5.4%	**6.0%**
Collective Voice	0.297	-0.241	-0.26	-0.156	0.245	0.199	0.143	-0.197	7.7%	**7.0%**
Flexibility	-0.147	-0.109	0.403	-0.334	0.214	0.312	0.321	-0.145	6.5%	**5.5%**
Excessive Hours	-0.303	-0.308	-0.242	-0.141	-0.074	0.312	-0.309	0.276	8.3%	**1.4%**
Managerial Duties	0.252	0.221	0.201	0.009	-0.075	-0.194	-0.491	-0.079	6.4%	**9.0%**
Short Term Prospects	0.006	-0.049	0.028	0.455	0.669	-0.341	0.106	-0.015	3.7%	**4.7%**
Long Term Prospects	0.124	-0.326	0.349	0.087	-0.101	-0.289	-0.201	0.3	6.9%	**5.8%**
Work Fatalities	0.165	-0.541	0.145	0.079	-0.167	-0.073	0.025	-0.09	7.3%	**4.5%**
Work Accidents	-0.056	-0.167	0.132	0.469	-0.549	0.036	0.332	-0.277	5.4%	**3.7%**
Work Illnesses	-0.3	0.458	-0.167	0.266	-0.145	0.068	0.097	-0.041	8.6%	**7.5%**
Proportion of variance	**30.8%**	**23.0%**	**13.9%**	**6.8%**	**6.0%**	**4.9%**	**3.7%**	**2.9%**	**92.1%**	**92.1%**

Appendix B – Missing values analysis of Understanding Society data

At the outset, it is useful to distinguish between two types of "missingness" when dealing with panel data such as Understanding Society:

- **Missing *cases*** occur when a respondent does not participate in a given wave of the survey. This is related to wave attrition, where respondents from previous waves attrit from the survey over time, thus becoming missing cases. These respondents will not be assigned a weight by Understanding Society in these waves, since they did not respond (this is sometimes referred to as not being "enumerated" in that wave).
- **Missing *values*** occur when a respondent who has missing values for a particular question which they should have been asked in that wave, but they otherwise participated in that wave. These respondents are not lost to attrition, but for whatever reason there is missing data for one or several variables of interest. These respondents will have been assigned a weight in a given wave.

Missing cases are a common issue in panel designs, and Understanding Society is no different. Analysis has found that as of Wave 11, its General Population Sample (GPS) lost 60.1%, and its Immigration and Ethnic Minority Boost (IEMB) sample – which was introduced in Wave 6 to increase the representativeness of new migrant groups and ethnic minorities – had lost 67.3%, of their respective initial wave respondents (Alvarez et al., 2023). These are addressed by Understanding Society through weighting methods to correct for non-response biases. The weights also ensure that groups who are deliberately over-represented in the survey, such as in the IEMB, are assigned a lower weight to reflect their actual distribution in the population.

On its own, weighting can address some issues with both missing values and missing cases. It can address many of the issues caused by non-response by making the survey more representative of the population of interest, and separate studies have indeed shown that Understanding Society's weighting methodology corrects for most sample biases (Alvarez et al., 2023; Lynn, 2011). However, Understanding Society advise that it is good practice to also impute missing values.[5] Because missing data is unlikely to be Missing Completely at Random, imputation rather than listwise deletion would be a more appropriate way to adjust for this issue.[6] I do not attempt to deal with missing cases, since these respondents are not assigned weights in the surveys used (presenting a considerable challenge about how to assign those weights) and they will only have data from other waves' responses to use as predictors for the imputation.

Pursuant to this, tables B.1 and B.2 show the proportion of weighted and unweighted missing cases for each indicator in the QoW index. I highlight cases where missingness exceeds 5% of respondents – a commonly-cited percentage threshold for imputation. I present both weighted and unweighted missing cases because the former gives an indication as to the effect that any missing cases could have on the analysis: the larger the *weighted* missing cases, the higher the potential difference between the observed data and the real-world labour market experience of UK workers.

---

[5] Understanding Society User Support Forum, 16 May 2023, 'Using weights when variables have some or many 'missing value' codes or NAs due to missing household level data', Support Ticket #1904, https://iserredex.essex.ac.uk/support/issues/1904.

[6] Understanding Society User Support Forum, 30 May 2022, 'Usual and last pay – question on imputation and missing values', Support Ticket #1708, https://iserredex.essex.ac.uk/support/issues/1708.

The tables show that, once weights are applied, missing cases are generally below 5%, but with some notable exceptions. Missing cases are a consistent problem for the Continuous Employment indicator. This is due to the effect of attrition: because this indicator is produced using some longitudinal data, people who respond to the given wave but who did not respond to the wave immediately prior to the given wave will cause missing values for this indicator. It is also an issue for many variables in Wave 6 of Understanding Society. Other studies have shown that a change in fieldwork agency in Wave 6 caused unusually high attrition in that wave (Benzeval et al., 2020, p. 20); my analysis shows this may causes issues for missing cases. It is also likely that the introduction of the Immigration and Ethnic Minority Booster (IEMB) sample in Wave 6 played a role here, with a number of new entrants in that wave were not asked some particular survey questions, with these respondents recording answers of "not available for IEMB" for some such questions.

Other than this, missing values increase over the time series for Long-term Prospects and the Health and Safety indicators – the only indicators where this occurs. They exceed 5% for Long-term Prospects in the final wave. Note that the proportion of missing values is the same for the Health and Safety indicators because they are all derived from the same variable, Standard Industrial Classifications (see Appendix D). In Wave 12, the question on personal pensions used to create the Pension indicator is exceptionally not included. The relevant employee pension questions continued to be asked in that wave, and there is good coverage of that data, but this poses an issue for the treatment of employees who do not have employer pensions and self-employed workers, who will be coded "Middle" if they have a personal pension.

Figure B.1 provides a breakdown of the cumulative (unweighted) proportion of missing values in each wave. The figure shows that missingness is disproportionately concentrated in a minority of respondents, with most respondents in all waves having no missing data:

- In all but one wave (Wave 6), around 75% of respondents have no missing values for any of the indicators in the QoW index in that wave.
- Around 15% of respondents are missing in one indicator. This is almost entirely accounted for by the Continuous Employment indicator.
- Three waves show an uptick in the proportion of respondents missing in 7 indicators. In every wave, 5 of these 7 indicators are the same: Continuous Employment, Pension, Short-Term Prospects, Autonomy and Collective Voice. This is particularly pronounced in Wave 6 (10%) and Wave 4 (6.4%), but is not an issue for other waves. The other 2 missing indicators of the 7 vary from respondent-to-respondent and wave-to-wave, with no strong relationship.

In the (rare) cases where data is Missing Completely at Random (MCAR), missingness would not pose a major issue: analysis would lose statistical power due to fewer respondents, but the missing data would be the same as the non-missing data. However, missing data in Understanding Society is recognised as not being MCAR. For example, analysis has shown that rates of attrition are higher for certain subgroups than others – with "youngsters, ethnic minorities, participants with poor health" and others more likely to attrit (Alvarez et al., 2023, p. 1). Whilst the initial wave of Understanding Society has been found to have been closely representative of the Census population at that time, subsequent waves have become increasingly less so due to different rates of attrition within sub-groups and the introduction of booster samples to reflect later changes in the UK population (Lynn and Borkowska, 2018). My own analysis (not presented here but discussed in Appendix C, further reinforces this, showing the probability of missingness in the QoW indicators related to the characteristics of workers.

In light of the above considerations, I impute missing values for most the indicators in the QoW index. Although the focus of this imputation is indicators with >5% missing values (especially Continuous Employment) and imputing Wave 12 personal pensions data, the process is used to impute missing values for all but five of the indicators: Earnings Sufficiency, Long-term Prospects and the Health and Safety indicators. This is because Earnings Sufficiency has virtually zero missingness due to it already being imputed by Understanding Society (Fisher et al., 2019). For the other three missingness is below 5% in most waves, and there are inherent challenges in imputing this data since it is determined by the occupation they are working in and the industry they are part of. Missing values for the remaining 10 indicators are imputed using multiple imputation using chained equations (MICE), through a process described in Appendix C.

Table B.1. Weighted proportion of QoW index members with missing values in each indicator, Wave 4 (2012-13) to Wave 12 (2020-21). Cases where missingness exceeds 5% coloured red.*

Dimension	Indicator	2012-13	2014-15	2016-17	2018-19	2020-21
Earnings	Earnings Sufficiency	0.0%	0.0%	0.0%	0.0%	0.0%
	Earnings Equity	0.7%	4.5%	1.8%	2.6%	3.2%
Insurance	Pension	1.7%	7.5%	2.9%	2.8%	No pers. pension
Security	Continuous Employment	13.4%	12.7%	11.8%	12.4%	12.9%
	Composite Security	0.1%	5.1%	0.5%	0.4%	0.6%
Autonomy & Voice	Autonomy	0.2%	3.4%	0.6%	0.6%	0.6%
	Collective Voice	2.2%	10.2%	4.5%	3.6%	3.8%
Work-life balance	Employee Flexibility	0.8%	8.3%	2.3%	2.1%	3.1%
	Excessive Hours	0.9%	2.5%	2.7%	3.1%	3.6%
Prospects	Managerial Duties	0.6%	3.5%	0.2%	0.3%	0.4%
	Short-term Prospects	0.0%	2.9%	0.1%	0.0%	0.1%
	Long-term Prospects	0.7%	1.0%	1.6%	5.2%	6.3%
Health & Safety	Work Fatalities	0.5%	1.3%	2.7%	3.7%	4.7%
	Work Accidents	0.5%	1.3%	2.7%	3.7%	4.7%
	Work Illnesses	0.5%	1.3%	2.7%	3.7%	4.7%

Table B.2. Unweighted proportion of QoW index members with missing values in each indicator, Wave 4 (2012-13) to Wave 12 (2020-21).*

Dimension	Indicator	2012-13	2014-15	2016-17	2018-19	2020-21
Earnings	Earnings Sufficiency	0.0%	0.0%	0.0%	0.0%	0.0%
	Earnings Equity	0.9%	4.7%	2.3%	3.0%	3.3%
Insurance	Pension	10.5%	20.3%	7.2%	5.1%	No pers. pension
Security	Continuous Employment	19.8%	22.4%	15.6%	14.5%	13.8%
	Composite Security	7.0%	14.8%	3.6%	2.1%	1.0%
Autonomy & Voice	Autonomy	9.3%	17.1%	5.0%	3.1%	1.2%
	Collective Voice	9.2%	19.6%	7.5%	5.2%	3.8%
Work-life balance	Employee Flexibility	9.4%	20.5%	6.5%	4.4%	3.4%
	Excessive Hours	1.1%	3.3%	3.1%	3.5%	3.8%
Prospects	Managerial Duties	0.7%	3.0%	0.3%	0.3%	0.4%
	Short-term Prospects	9.1%	16.7%	4.4%	2.4%	0.8%
	Long-term Prospects	0.9%	1.8%	2.2%	5.2%	6.1%
Health & Safety	Work Fatalities	0.6%	2.4%	3.1%	4.0%	4.5%
	Work Accidents	0.6%	2.4%	3.1%	4.0%	4.5%
	Work Illnesses	0.6%	2.4%	3.1%	4.0%	4.5%

* Note missingness for the Flexibility indicator is a proportion of employees only. Missingness for all other indicators a proportion of all workers in the QoW index, i.e. everyone in paid employment or away from a paid job they usually do when interviewed.

Figure B.1. Cumulative missingness. Unweighted proportion of respondents by wave with 0 (none) to all (15) QoW indicators missing (excl. Wave 12 personal pensions).

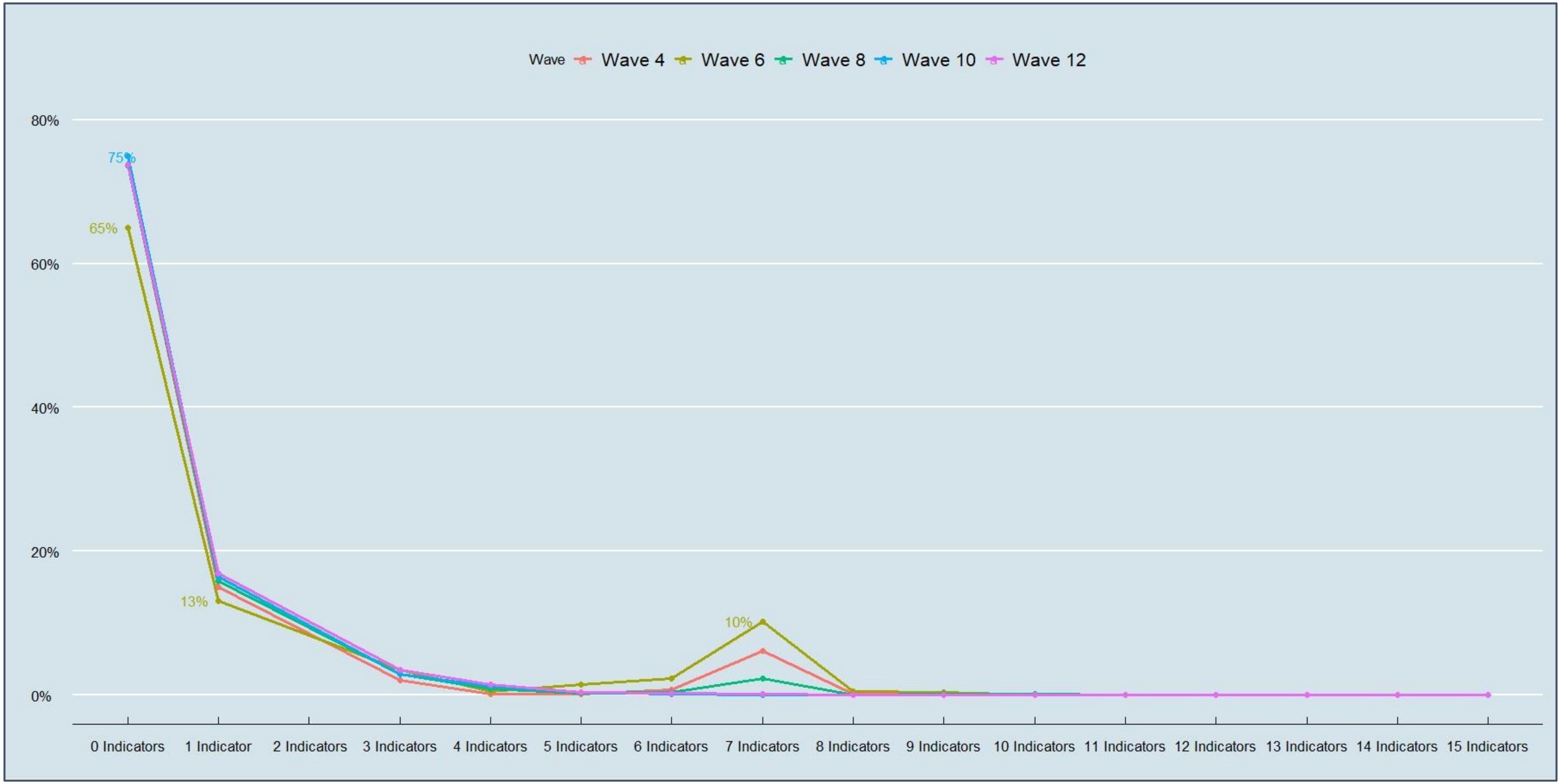

Appendix C – Multiple imputation method for missing data

Multivariate Imputation using Chained Equations (MICE) is a well-recognised imputation method which has seen growing use in the social sciences. There is an extensive and well-supported package in R, called MICE, which can be used to impute the data (Van Buuren and Groothuis-Oudshoorn, 2011). It is also the same method used for most of the imputation of Understanding Society's income data (Fisher et al., 2019, p. 31).

MICE requires missing data to be Missing At Random (MAR); it need not be MCAR for the imputation to be unbiased. This means that, where the cause of missingness can be controlled for using observable data, MICE is an appropriate method (Azur et al., 2011). It should be noted that imputed data does not necessarily have to be similar to non-imputed data in order for the process to work: where missingness is accounted for by observed characteristics, then it is consistent for the imputed data to be different from the (non-imputed) non-missing data. However, MICE may introduce bias in cases where the data is Missing Not at Random (MNAR).

In practice this can be very difficult to establish, and almost impossible to achieve in virtually all cases. (Graham, 2009, p. 567) advocates viewing data along a spectrum of pure MAR and pure MNAR, because "MCAR, pure MAR and pure MNAR really never exist because the pure form of any of these requires almost universally untenable assumptions." (Stuart et al., 2009, p. 1134) further argue that "MAR, while empirically unverifiable, is often a reasonable assumption to make unless substantive knowledge about the data or data collection process indicates that the missingness may depend on unobserved values."

In order to minimise the risk of MNAR, consistent with other approaches to MICE, I take an inclusive rather than a restrictive approach to including variables in the imputation model (Collins et al., 2001, p. 331) – including various auxiliary variables not cogent to my analysis, but associated with missingness, in addition to my analysis variables. In order to be identified for selection, such variables should be (Collins et al., 2001) (a) "Potential causes or correlates of the missingness itself"; and/or (b) "correlated with the variables that have missing values, whether or not they are related to the mechanism of missingness."

The following variables were identified as potentially useful predictors for the imputation. These draw from the same indicators used for the imputation of income data in Understanding Society (Fisher et al., 2019, pp. 33–34):

- Income variables:
  - Personal income
  - Household income
- Various individual characteristics, including analysis variables used in this paper:
  - Sex
  - Ethnicity
  - Age (+ age-squared)
  - Interaction terms for sex & ethnicity and sex & age
  - Region of residence
  - Number of dependent children
  - Number of non-working adults in the household
  - Individual physical and mental health
- Proxies for deprivation/disadvantage:

- National Statistics Socio-Economic Classification (NS-SEC) of individual's first or second job[7] after leaving full-time education
  - Highest NS-SEC of either of their parents when individual was aged 14
  - Highest educational qualification of the individual
  - Housing tenure
- Characteristics of the job or workplace:
  - Hours worked in all paid jobs
  - Whether self-employed (for indicators where employee and self-employed coding arrangements the same)
  - The number of employees in the individual's workplace[8]
- Fixed effects for year (i.e. wave of interview)

In order to identify which of these variables to include in the multiple imputation models, various regressions (not presented here) of the effect of these variables on both missingness and indicator scores were run. These consisted of:

- Logistic regressions on the probability of missingness for individual variables (with Wave 12 pensions examined separately) and the scores for binary variables;
- Ordered logit regressions of the effect of these variables on both (a) the number of indicators QoW the individual is missing in and (b) scores for continuous indicators.

These found that, as predicted, the missing population has different characteristics to the non-missing population. For example, missingness is significantly related to being younger, being male, living in private rented or social housing and working for a smaller employer. The interaction terms and individual NS-SEC were insignificant in most models, giving rise to concerns about multicollinearity, and so were excluded from most models. Region of residence was also insignificant in most cases but was retained due to its importance as an analysis variable.

Three distinct types of multiple imputations were carried out for each individual indicator depending on the nature of the indicator being imputed. All were imputed using a Predictive Mean Matching (PMM) method, using 25 iterations. Note that imputation has not been carried out on the four continuous indicators in the QoW index:

- I use the same, joint process for imputing five variables where there is missing data, and of the same structure (ie all categorical or all binary), for both employees and the self-employed: Earnings Equity, Composite Security, Autonomy, Excessive Hours, Managerial Duties and Short-Term Prospects.
- An employee-only imputation is carried out for three indicators where only employees have missing data: Continuous Employment, Collective Voice, and Employee Flexibility.
- The Pensions indicator treated differently. It is the only indicator where both (a) there is missing data for both employees and the self-employed and (b) the maximum scores both can get differ (0 or 1 for self-employed; 0, 1 or 2 for employees). It is also unique in having missing data due to the lack of the required survey question in Wave 12, meaning the missing

[7] This is a composite of two variables in Understanding Society: one asking respondents the NS-SEC of their first job after leaving full-time education; and the other asking them the NS-SEC of their second job. To minimise missingness in this variable, I create a combined indicator which takes the value of the higher of the two estimates where both are present, or goes with either where the other is not present. Note that parental NS-SEC is also used in the imputation as an analysis variable, and is created using a similar process.

[8] Similar to the above, this is a composite variable combining two separate questions for employees and the self-employed, together with a separate question on whether self-employed hire their own staff. Where self-employed say they do not hire their own staff, I code them the lowest score in this variable (workplace with 1-2 employees).

data in that wave has very different characteristics to that in others. Separate imputations are carried out for employees and the self-employed, and then again for the Wave 12 data.

Table C.1 presents the (unweighted) differences between imputed and non-imputed data, broken down by employees and self-employed. There is a strong consistency in the imputed and non-imputed data for most of the indicators. Where this is not the case, there are logical reasons for this justified by the preceding observations. For example there is a general tendency for most (though not all) imputed indicator scores to be slightly worse than non-imputed scores, although with self-employed workers tending to be assigned values around the middle.

To conclude, Table C.2 brings the imputed and non-imputed data together, presenting the (weighted) uncensored headcount ratios for the full data (imputed and non-imputed) alongside the difference in brackets were the headcount ratio for the non-imputed data only used instead. The data shows that, in practice, imputation has a very minor effect on the headcount ratios. The most pronounced differences are seen with the Continuous Employment indicator, but even in this case they do not affect overall trends in the data. There are several reasons for this:

- For most variables, the level of difference is a reflection of the low level of weighted missingness for the indicators anyway. Because there are relatively few missing values in most indicators, the effect that imputation can have on the overall conclusions about trends is minor.
- As set out in Appendix B, missingness is in any event higher amongst respondents who are weighted less in the survey.

Table C.1. Cross-tabulation of difference between unweighted uncensored headcount ratio between imputed and non-imputed data, broken down by employees and self-employed. Pooled data from all waves.

Dimension	Indicator	Score	Employees		Self Employed	
			Non-Imputed	Imputed	Non-imputed	Imputed
**Earnings**	Earnings Equity	Worst	9.9%	11.7%	41.9%	39.4%
		Middle	49.3%	46.4%	26.5%	38.6%
		Best	40.8%	42.0%	31.6%	22.0%
**Insurance**	Pension	Worst	30.9%	43.1%	84.7%	83.2%
		Middle	2.6%	3.0%	15.3%	16.8%
		Best	66.6%	53.9%	--	--
**Security**	Continuous Employment	Worst	11.4%	14.7%	--	--
		Middle	13.2%	16.0%	--	--
		Best	75.4%	69.2%	--	--
	Composite Security	Worst	16.6%	19.1%	19.1%	22.0%
		Best	83.4%	80.9%	80.9%	78.0%
**Autonomy and Voice**	Autonomy	Worst	12.0%	12.2%	1.8%	2.0%
		Middle	38.7%	39.1%	11.8%	14.4%
		Best	49.4%	48.7%	86.4%	83.7%
	Collective Voice	Worst	52.7%	57.6%	--	--
		Best	47.3%	42.4%	--	--
**Work-life balance**	Employee Flexibility	Worst	22.2%	26.1%	--	--
		Middle	50.6%	51.0%	--	--
		Best	27.2%	22.9%	--	--
	Excessive Hours	Worst	13.6%	14.3%	22.5%	18.4%
		Middle	39.9%	35.1%	27.7%	44.6%
		Best	46.6%	50.6%	49.8%	37.0%
**Prospects**	Managerial Duties	Worst	64.2%	72.8%	83.4%	90.5%
		Best	35.8%	27.2%	16.6%	9.5%
	Short-Term Prospects	Worst	48.8%	42.3%	68.4%	61.3%
		Middle	35.4%	36.0%	23.0%	30.6%
		Best	15.8%	21.7%	8.6%	8.0%

Table C.2. Time series of weighted uncensored headcount ratio scores using imputed and non-imputed data combined, with differences versus using non-imputed data only in brackets, 2012-13 to 2020-21. Note that due to rounding differences may not always net to zero.

Dimension	Indicator	Score	2012-13	2014-15	2016-17	2018-19	2020-21
**Earnings**	Earnings Equity	Worst	19.9%	12.9% (-0.2%)	14.9% (-0.1%)	13% (0.2%)	8.5% (-0.3%)
		Middle	40.0%	48.1% (0.3%)	45.6%	48.8% (0.1%)	50.7% (0.1%)
		Best	40.1% (0.1%)	39.1% (-0.2%)	39.4% (0.2%)	38.1% (0.2%)	40.8% (0.2%)
**Insurance**	Pension	Worst	51.8% (-0.7%)	41.9% (-1.4%)	36% (-0.6%)	30.8% (-0.6%)	28.9%(--)
		Middle	5% (0.1%)	5.6% (0.1%)	3.9% (-0.1%)	3.7% (0.1%)	2.9%(--)
		Best	43.2% (0.7%)	52.5% (1.3%)	60.2% (0.6%)	65.5% (0.7%)	68.2%(--)
**Security**	Continuous Employment	Worst	23.9% (2.1%)	25.1% (3.4%)	25.1% (1.8%)	24.1% (1.8%)	23% (1.5%)
		Middle	12.6% (-1%)	12.5% (-1.2%)	12.2% (-0.7%)	10.8% (-0.6%)	10.6% (-0.8%)
		Best	63.5% (-1.1%)	62.3% (-2.1%)	62.8% (-1.1%)	65.1% (-1.2%)	66.4% (-0.7%)
	Composite Security	Worst	17% (-0.1%)	17.5% (-0.4%)	16.4% (-0.1%)	16% (-0.1%)	19.4% (-0.1%)
		Best	83% (0.1%)	82.5% (0.4%)	83.6% (0.1%)	84% (0.1%)	80.6% (0.1%)
**Autonomy and Voice**	Autonomy	Worst	11.5% (0.1%)	9.9%	10.3%	10.6%	10.5%
		Middle	34.3% (-0.1%)	34.0%	35.0%	36.4%	36.2%
		Best	54.2% (0.1%)	56.2% (-0.1%)	54.8%	53.0%	53.3%
	Collective Voice	Worst	59.2%	59.2% (0.8%)	60.5%	60.2%	59.8% (-0.1%)
		Best	40.8%	40.8% (-0.8%)	39.5%	39.8%	40.2% (0.1%)
**Work-life balance**	Employee Flexibility	Worst	24.1% (-0.3%)	22.4% (-0.4%)	22.9% (-0.5%)	21.5% (-0.3%)	21.2% (-0.3%)
		Middle	48.1% (-0.3%)	49.7% (-0.6%)	52.7% (0.3%)	52.5% (0.1%)	51.6% (0.1%)
		Best	27.9% (0.5%)	27.8% (1.1%)	24.4% (0.2%)	26% (0.2%)	27.3% (0.1%)
	Excessive Hours	Worst	14.9%	15.3%	15.0%	14.8%	13.5%
		Middle	38.5%	36.8%(0.1%)	38.2%(0.1%)	38.1% (0.1%)	39.2%
		Best	46.6%	47.9%(-0.1%)	46.8%	47.1%	47.2%
**Prospects**	Managerial Duties	Worst	66.5% (0.1%)	67.2% (-0.4%)	67.2%	67.4%	66.7% (-0.1%)
		Best	33.5% (-0.1%)	32.8% (0.4%)	32.8%	32.6%	33.3% (0.1%)
	Short-Term Prospects	Worst	48.7% (0.1%)	47.9% (1%)	51.2% (-0.1%)	53.5%	57.0%
		Middle	34.4%	36.0%	34.2% (0.1%)	32.3% (-0.1%)	30.8%
		Best	16.9% (-0.1%)	16.1% (-1%)	14.7% (-0.1%)	14.3% (-0.1%)	12.2%

Appendix D – Health and Safety dimension

Workplace health and safety is a recognised as an important dimension in job quality, and one that is usually not correlated with other indicators and dimensions of job quality indices. Data on health and safety is available in the European Working Conditions Survey, and is used in the European Job Quality Index (Muñoz de Bustillo et al (2011). However, Understanding Society has no data on workplace health and safety.

This paper addresses this shortcoming by introducing three new health and safety indicators into Understanding Society using data from external surveys:

- **Workplace Accidents,** using self-reported incidences of workplace accidents in the Labour Force Survey (LFS), covering the same period as is covered in the QoW index in Understanding Society (2012-2022).
- **Workplace Illnesses,** also using LFS data on self-reported illnesses caused or made worse by employers, covering the period 2012-2022.
- **Workplace Fatalities**, which is drawn from public data reported to the Health and Safety Executive under the Reporting of Injuries, Diseases and Dangerous Occurrences Regulations (RIDDOR),[9] covering the 6-year period 2014/15 to 2019/20.

Taken together, this data is designed to cover the full spectrum of workplace health and safety issues – from workplace fatalities covered in RIDDOR to more mild-to severe workplace accidents and injuries in the LFS. All the data is of incidents which took place in the course of work, or was caused or made worse by a job.

This data is introduced into Understanding Society through the following process:

1. A series of matrices of health and safety incidents by Standard Industrial Classification (SIC) are produced.[10]
2. These are then converted into incidences of workplace fatalities, accidents, and injuries per 100,000 workers in that industry,[11] using weighted estimates of industry size from the LFS.
3. The data from these matrices are then read into Understanding Society based on a common variable: SICs. This effectively tells us the rate of accidents, illnesses and fatalities per 100,000 in the industry in which each worker in Understanding Society works in their main job.
4. Finally, these are then turned into three equally-weighted indicators for the Health and Safety dimension in the QoW index. Because the incidence rates differ by each indicator, the standardised scores are incorporated into the index by first turning the variable scores into standard units, and then converting these scores into a 0-2 scale.

Because of the differing nature of, and sources for, the data, these are incorporated into Understanding Society at slightly different levels of aggregation of the SICs and the matrices cover different time periods. This is discussed in the below subsections.

---

[9] See Health and Safety Executive, *Index of Data Tables – RDIND: Table 1: Work-related fatal injuries to workers (employees and the self-employed) in Great Britain, by detailed industry*. Last updated August 2023.

[10] These use the SIC 2007, which is the current most up-to-date method for classifying businesses in the UK based on the type of economic activity they are engaged in.

[11] This is simply to align with how these incidents are reported by the Health and Safety Executive, to ease comparison with other published data.

D.1 Work accidents and illnesses

In Q1 (January-March) of every year, the LFS asks respondents a set of questions about any workplace accidents and injuries which have occurred over the past 12 months. These are the basis for most data on self-reported mild-to-severe workplace health and safety issues reported by the Health and Safety Executive. The QoW index uses data from two binary questions:

- **Workplace accidents:** *"Thinking of the twelve months since* [full date], *have you had any accident resulting in injury at work or in the course of your work?"*
- **Workplace illnesses:** "[Apart from the accident you have told me about], *within the last twelve months have you suffered from any illness, disability or other physical or mental problem that was caused or made worse by your job or by work you have done in the past?"*

An additional set of questions in the survey confirm whether the job which caused the accident or their illness was their main job or their second job,[12] which allows us to identify the SIC of the job which caused or made worse the incident.

I create a series of matrices of confirmed incidences of workplace accidents and illnesses by the SIC of the main job. To reflect changes over time, six different matrices of accidents and illnesses are created using data pooled into six pairs of years (see Table 1). Each matrix is used to create data for the respective wave of Understanding Society. These align directly with the time period covered in the relevant wave of Understanding Society, with just one exception: in Wave 4 (2012-13), work illnesses data is drawn from Q1 2011 rather than Q1 2013 because the relevant question was exceptionally not asked in the LFS in Q1 2013. A total of 446,749 relevant responses are captured in these matrices, ranging from 62,418 in 2020-21 to 80,744 in 2014-15.

These matrices report the weighted number of incidences of workplace accidents and illnesses for the period covered broken down by 21 different SICs. The use of pooled data from a larger sample of surveys allows matrices to be created at a higher level of granularity than is reported in some other national statistics, yet still, there are a small number of instances where there are a low number of respondents with some of the less common SICs. This occurs in the following instances; where this is the case, these are treated in a way which is consistent with the way the HSE treats these categories in their own data:

- Mining and quarrying (Category B; SICs 05-09) is kept separate despite the low (~100-200 p.a.) number of respondents with this SIC. This is in line with HSE reporting, since this is a distinct category which is difficult to merge with other data.
- Electricity, gas, steam and air conditioning supply (Category D; SIC 35) is kept separate despite the low (~200-500 p.a.) number of respondents, again due to the lack of a comparable category, and in line with the HSE's approach.
- Water supply; sewerage, waste management and remediation activities (Category E; SICs 36-39) covers ~300-600 respondents p.a., but again is kept separate in HSE reporting.
- Arts, entertainment and recreation (R), other service activities (S), activities of households with employers/for own use (T) and activities of extraterritorial organisations and bodies (U) are merged together due to the low number of respondents, in line with HSE reporting of fatalities data.

Tables D.2 and D.3 report rates of workplace accidents and injuries by each of these SIC categories for the latest period covered (2020-21), ranking each occupational group by the number of

[12] These are WCHJB for workplace accidents and WCHJB3 for illnesses.

incidences per 100,000 workers in that industry. Even without statistical analysis, it is evident that the incidence of workplace accidents and illnesses is only weakly correlated, with many industries with high workplace accidents reporting low rates of work illness, and vice-versa.

D.2 Work fatalities

The LFS cannot provide data on workplace fatalities, since its workplace accidents and injuries data is self-reported. This poses a problem for any health and safety dimension in a job quality index because (a) Workplace fatalities capture an important aspect of the *severity* of workplace accidents and (b) there is no guarantee that incidences of workplace fatalities are correlated with workplace accidents.

To create an indicator of workplace fatalities, I use RIDDOR data on employer-reported incidences of workplace fatalities. These are published by the Health and Safety Executive for each year going back to 2014/15, and broken down by a smaller number of SIC groupings – 11 – than is available in the more detailed LFS data on workplace accidents and injuries. Broadly consistent with the data on workplace accidents and illnesses, incidents reported to RIDDOR must "arise from a work-related accident, including an act of physical violence to a worker", but "with the exception of suicides."[13]

Because a small number of incidences of workplace fatalities are reported every year, the rate of incidents is subject to considerable variation if data is broken down by year. This means that time series analysis of any change in fatalities over time is not possible. Instead, a matrix of incidences is created by pooling data for six years, covering the whole period under study. This has the disadvantage of not being able to capture changes over time in the same way as can be captured using the workplace accidents and illnesses indicators (changes over time will only be captured where these arise from changes in the industrial composition of the workforce, and not any improvements in workplace safety within the same industries). Table D.4 presents workplace fatalities per 100,000 ranked by each of these industrial groupings.

[13] See Health and Safety Executive, *Types of reportable incidents*, accessed 08/11/23.

Table D.1. Labour Force Survey matrices used for each wave of the QoW Index in Understanding Society.

Understanding Society wave	Labour Force Surveys used for matrices	Number of relevant respondents in LFS
Accidents – Wave 4 (2012-13)	Q1 2012 & Q1 2013	79,703
Illnesses – Wave 4 (2012-13)	Q1 2011 & Q1 2012	80,192
Wave 6 (2014-15)	Q1 2014 & Q1 2015	80,733
Wave 8 (2016-17)	Q1 2016 & Q1 2017	73,452
Wave 10 (2018-19)	Q1 2018 & Q1 2019	70,251
Wave 12 (2020-21)	Q1 2020 & Q1 2021	62,418

Table D.2. Rank of workplace accidents per 100,000 by large SIC category in LFS, pooled Q1 2020 & Q1 2021 data.

SIC 2007	Description	Industry size as % of workforce (LFS estimate)	Number of workplace accidents reported in LFS	Workplace accidents per 100,000 workers in industry
**1**	Agriculture, forestry and fishing	0.91%	16,999	3,746
**6**	Construction	6.51%	102,637	3,168
**8**	Transportation and storage	4.70%	59,106	2,523
**9**	Accommodation and food service activities	4.83%	51,505	2,141
**3**	Manufacturing	8.82%	83,667	1,904
**5**	Water supply; sewerage, waste management and remediation activities	0.72%	6,764	1,884
**7**	Wholesale and retail trade; repair of motor vehicles and motorcycles	11.74%	109,292	1,870
**17**	Human health and social work	13.67%	123,240	1,810
**15**	Public administration and defence; compulsory social security	7.08%	61,248	1,737
**16**	Education	10.96%	85,880	1,574
**14**	Administrative and support service activities	4.48%	34,732	1,556
**12**	Real estate agents	1.22%	8,558	1,404
**18to21**	Arts, entertainment and recreation; other service activities; activities of households as employers/for own use; and activities of extraterritorial organisations and bodies	5.86%	38,876	1,332
**4**	Electricity, gas, steam and air conditioning supply	0.64%	3,558	1,124
**2**	Mining and quarrying	0.36%	1,920	1,086
**13**	Professional, scientific and technical activities	8.36%	25,950	623
**10**	Information and communication	4.91%	8,373	343
**11**	Financial and insurance activities	4.23%	7,141	339

Table D.3. Rank of workplace illnesses per 100,000 by large SIC category in LFS, pooled Q1 2020 & Q1 2021 data.

SIC 2007	Description	Industry size as % of workforce (LFS estimate)	Number of workplace illnesses reported in LFS	Workplace illnesses per 100,000 workers in industry
17	Human health and social work	13.62%	123,240	6,374
15	Public administration and defence; compulsory social security	7.07%	61,248	4,988
1	Agriculture, forestry and fishing	0.92%	16,999	4,533
16	Education	11.00%	85,880	4,502
4	Electricity, gas, steam and air conditioning supply	0.64%	3,558	4,143
14	Administrative and support service activities	4.49%	34,732	3,785
18to21	Arts, entertainment and recreation; other service activities; activities of households as employers/for own use; and activities of extraterritorial organisations and bodies	5.85%	38,876	3,350
6	Construction	6.49%	102,637	3,302
11	Financial and insurance activities	4.25%	7,141	3,276
3	Manufacturing	8.83%	83,667	3,131
7	Wholesale and retail trade; repair of motor vehicles and motorcycles	11.71%	109,292	2,998
13	Professional, scientific and technical activities	8.39%	25,950	2,829
12	Real estate agents	1.22%	8,558	2,793
8	Transportation and storage	4.71%	59,106	2,787
5	Water supply; sewerage, waste management and remediation activities	0.73%	6,764	2,786
10	Information and communication	4.92%	8,373	2,552
9	Accommodation and food service activities	4.81%	51,505	2,330
2	Mining and quarrying	0.36%	1,920	1,971

Table D.4. Rank of workplace fatalities per 100,000 by large SIC in UK workforce, 2014/15 to 2019/20.

Category and 2-digit SIC grouping (2007)	Description	RIDDOR Fatal injuries, 2014/15 to 2019/20	Industry size as % of workforce (LFS estimates)	Fatalities per 100,000 workers in industry
**A (01-03)**	Agriculture, forestry and fishing	167	1.1%	9.33
**E (36-39)**	Water supply; sewerage, waste management and remediation activities	51	0.7%	4.50
**F (41-43)**	Construction	223	7.1%	2.00
**B (05-09)**	Mining and quarrying	12	0.4%	1.78
**H (49-53)**	Transportation and storage	86	4.9%	1.12
**C (10-33)**	Manufacturing	118	9.4%	0.80
**D (35)**	Electricity, gas, steam and air conditioning supply	7	0.6%	0.74
**G,I (45-47,55-56)**	Wholesale and retail trade; repair of motor vehicles and motorcycles; accommodation and food service activities	57	18.1%	0.20
**J-N (58-82)**	Information and communication; financial and insurance activities; real estate activities; professional, scientific and technical activities; administrative and support service activities	57	21.3%	0.17
**R-U (90-99)**	Arts, entertainment and recreation; other service activities; activities of households as employers; undifferentiated goods- and services-producing activities of households for own use; activities of extraterritorial organisations and bodies	14	5.8%	0.15
**O-Q (84-88)**	Public administration and defence; compulsory social security; education; human health and social work activities	35	30.4%	0.07

Figure D.1. Time series comparison of weighted mean **work accidents** per 100,000 in Understanding Society vs. the Labour Force Survey, 2012-13 to 2020-21.

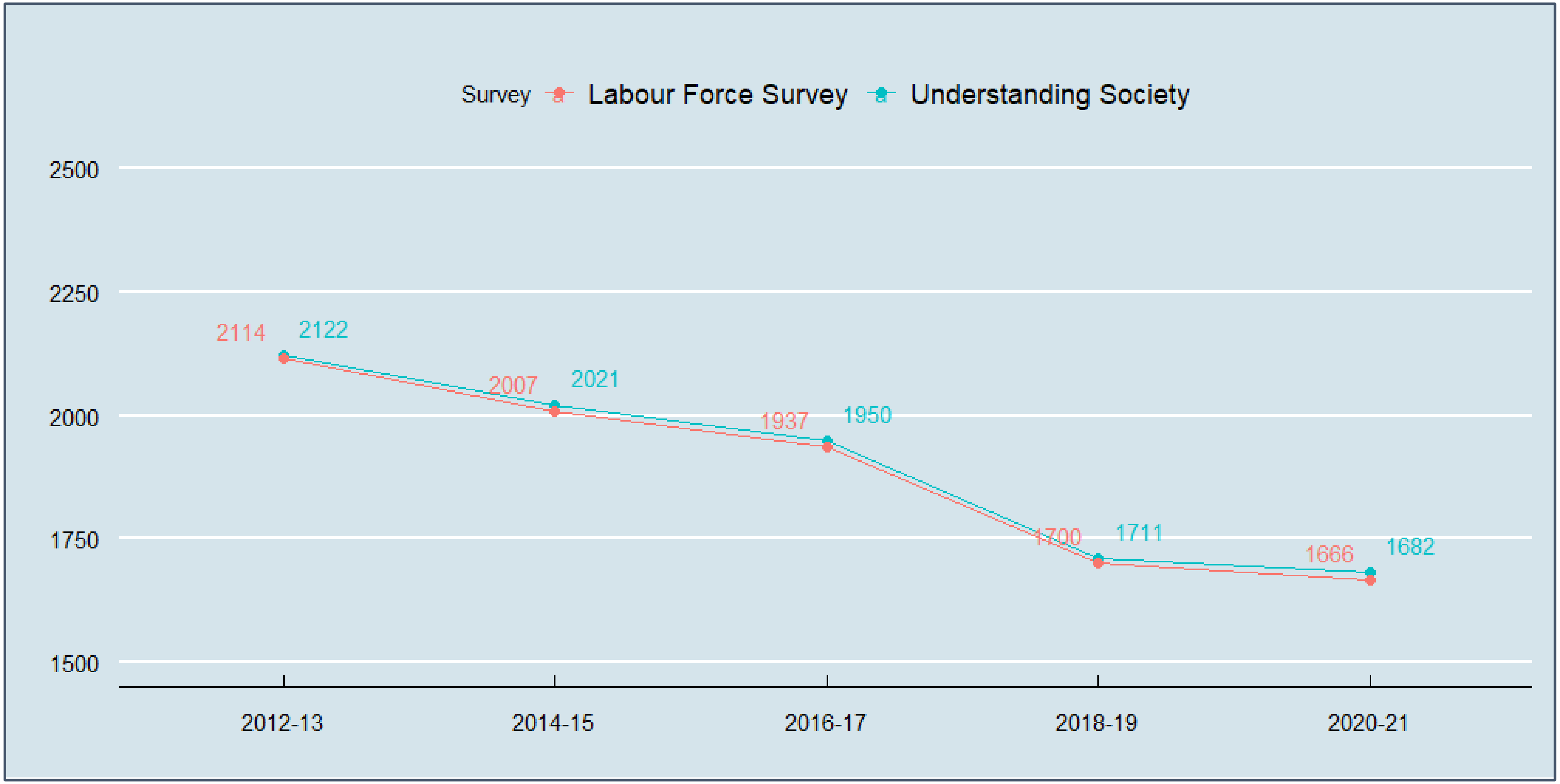

Figure D.2. Time series comparison of weighted mean **work illnesses** per 100,000 in Understanding Society vs. the Labour Force Survey, 2012-13 to 2020-21.

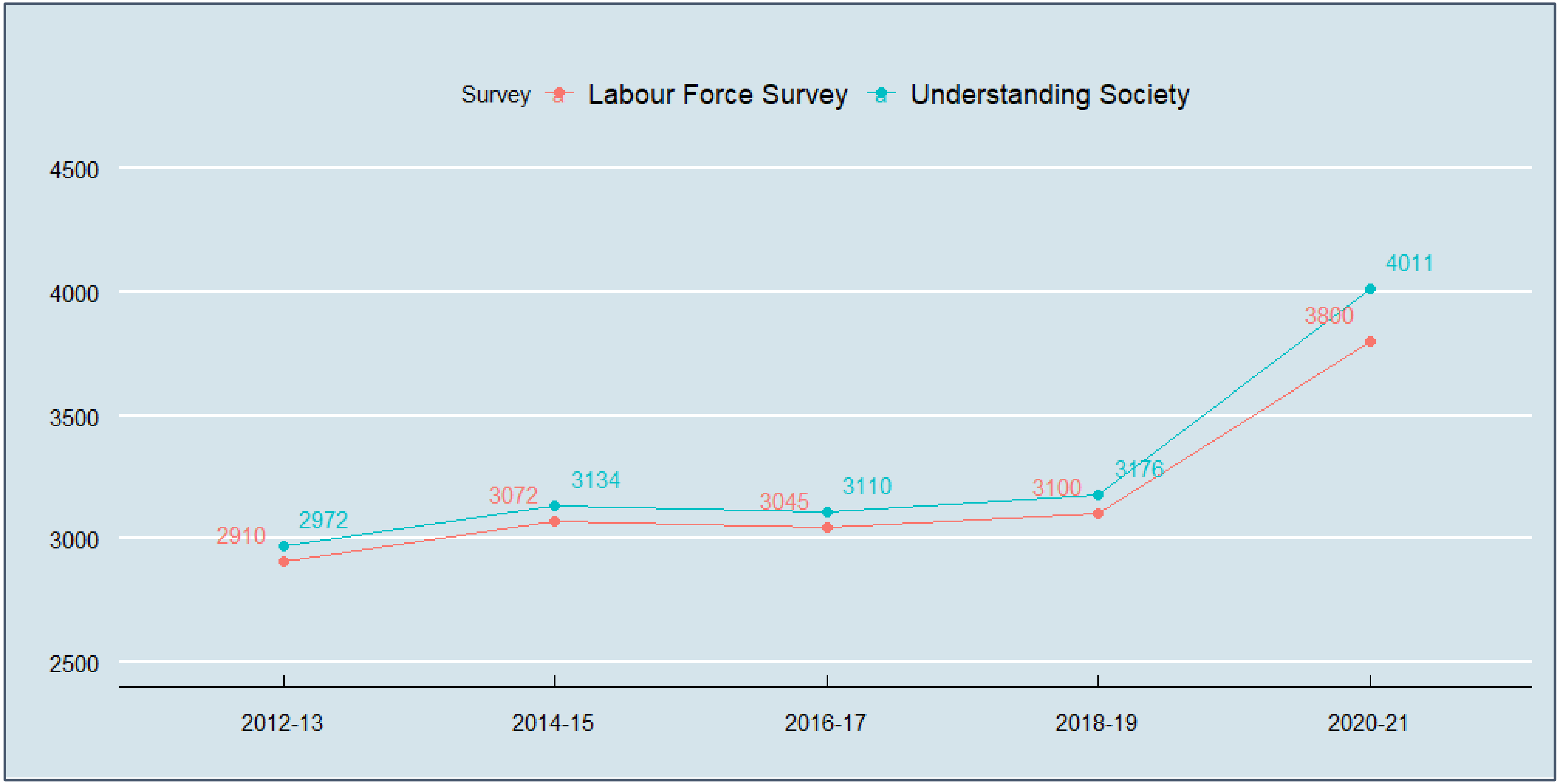

Figure D.3. Time series comparison of weighted mean **work fatalities** per 100,000 in Understanding Society vs. the Labour Force Survey, 2012-13 to 2020-21.

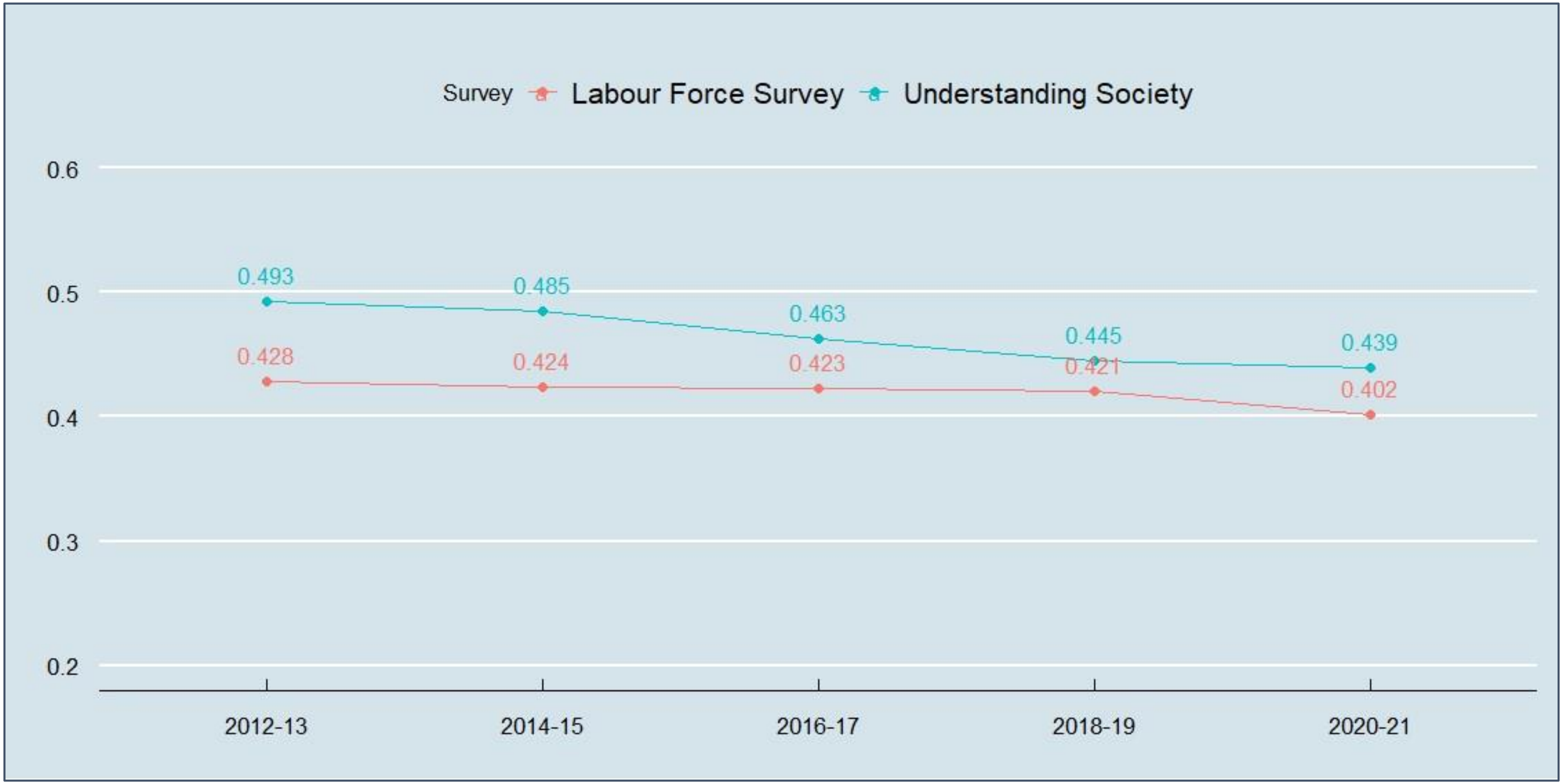

Table D.5. Comparison of the weighted SIC composition of Understanding Society vs. the Labour Force Survey, Wave 12 (2020-21) vs. pooled data from Q1 2020 and Q1 2021 of the LFS.

SIC 2007	Description	Industry size as % of workforce (LFS estimates)	Industry size as % of workforce (Understanding Society estimates)	Difference (LFS minus Understanding Society estimate)
**1**	Agriculture, forestry and fishing	0.91%	0.71%	-0.20%
**2**	Mining and quarrying	0.36%	0.14%	-0.22%
**3**	Manufacturing	8.82%	8.32%	-0.50%
**4**	Electricity, gas, steam and air conditioning supply	0.64%	0.56%	-0.08%
**5**	Water supply; sewerage, waste management and remediation activities	0.72%	0.72%	0.00%
**6**	Construction	6.51%	4.94%	-1.57%
**7**	Wholesale and retail trade; repair of motor vehicles and motorcycles	11.74%	12.57%	0.83%
**8**	Transportation and storage	4.70%	4.33%	-0.37%
**9**	Accommodation and food service activities	4.83%	4.20%	-0.63%
**10**	Information and communication	4.91%	3.72%	-1.19%
**11**	Financial and insurance activities	4.23%	3.47%	-0.76%
**12**	Real estate agents	1.22%	1.12%	-0.10%
**13**	Professional, scientific and technical activities	8.36%	7.33%	-1.03%
**14**	Administrative and support service activities	4.48%	4.02%	-0.46%
**15**	Public administration and defence; compulsory social security	7.08%	7.69%	0.61%
**16**	Education	10.96%	12.46%	1.50%
**17**	Human health and social work	13.67%	19.06%	5.39%
**18to21**	Arts, entertainment and recreation; other service activities; activities of households as employers/for own use; and activities of extraterritorial organisations and bodies	5.86%	4.65%	-1.21%

Table D.6. Comparison of the distribution of indicator scores for work accidents, work illnesses and work fatalities. All waves pooled together.

Indicator	Min.	1st Quartile	Median	Mean	3rd Quartile	Max.
**Work Accidents**	0	0.579	0.647	0.6435	0.719	1
**Work Illnesses**	0	0.396	0.733	0.5365	0.665	1
**Work Fatalities**	0	0.971	0.978	0.9315	1	1

D.3 Robustness and representativeness

To test the representativeness of Understanding Society data, Figures D.1-D.3 compare a time series of weighted mean workplace accidents, injuries and fatalities per 100,000 from Understanding Society vs. the Labour Force Survey. Even though the matrices used to create both indicators are the same, a difference in figures would suggest that one survey systematically under- or over-represents certain occupational groups. The data shows that Understanding Society very closely aligns with the LFS for workplace accidents. It tends to report higher incidences of workplace illnesses, although not by much, and the trends for both are broadly similar. The largest difference can be seen with workplace fatalities, although again the difference is small: the LFS reports a lower rate of workplace fatalities than Understanding Society, and the trends in both surveys are not quite the same.

Table D.5 shows that, indeed, there are differences in the representation of SICs between both surveys. Whilst in most cases the differences in these weighted proportions are minor, there are noteworthy differences in some industries. In particular, Understanding Society reports a markedly higher proportion of workers in human health and social work – an industry with significantly higher levels of workplace illnesses. This in itself would explain the differences in workplace illnesses we see when comparing Understanding Society and the LFS.

Finally, some minor technical comments on the nature of the data warrant mention:

- The denominator used for creating incidence matrices of workplace accidents vs. workplace illnesses differs slightly, which has implications for pandemic trends in workplace accidents. Workplace illnesses are measured as a proportion of those who have done work in the past 12 months, whereas workplace illnesses use a looser denominator including those off work, on furlough, etc. This is partly reflected in the slightly different estimates of the size of these SIC groupings found in the two matrices (see Tables C.2-C.3). This has no implications for the methodology used to generate these matrices, but it does have implications for trends in workplace accidents during the pandemic (see below).
- As documented by the HSE, and as is apparent from Figures C.2-C.3, the pandemic has changed trends in workplace accidents and workplace illnesses. This has led to a marked rise in workplace illnesses and a fall in workplace accidents. The fall in workplace accidents predates the pandemic, but LFS data also shows a different trend during and since the pandemic: a sharp rise in workplace accidents in 2020, followed by an even sharper fall in workplace accidents from 2021 which has been sharper than the years predating it. Some of the fall in accidents is accounted for by the aforementioned issue with the denominator used for accidents, which has captured a large part of the furloughed population. However, HSE analysis has found that even if a different incidence rate is constructed using incidences per 100 million hours worked, a post-pandemic rise then fall in the rate of workplace accidents is still apparent – albeit a less sharp one than you get when using per 100,000 workers (HSE, 2021). When reading the data into Understanding Society data, I retain the use of the 100,000 workers measure. Because Understanding Society combines data from multiple years (merging 2020 and 2021) the trends are much smoother than is apparent in LFS data, with the rise in accidents in 2020 being more than cancelled out by the fall in 2021.
- A consistent number (~7000) respondents in the Labour Force Survey report missing data for workplace accidents and workplace illnesses. This accounts for virtually all of the missingness However, the ONS Social Surveys team have confirmed that this is caused by a procedure of bringing forward respondents from previous quarters to improve response rates. This has the effect of these brought forward respondents being reported missing in Q1 of the year because

that question is only asked in Q1, but there is no reason to assume the characteristics of these respondents are any different to those who do not respond. This, plus supplementary analysis, confirms that these can be treated as close to Missing Completely at Random.[14] These missing data are therefore not imputed, and incidence rates are calculated using the data from respondents only.

- Consideration was given to more directly imputing the data into Understanding Society, for example using multivariate imputation using chained equations for a range of analysis and auxiliary variables, but this was rejected for largely normative reasons. The only feasible way of doing this would be to directly impute the accidents and illness data to impute an estimate of *whether or not an accident or illness occurred* for every Understanding Society respondent. Once done, this data in-itself would be of limited use for a job quality index: since imputation would not tell us whether the respondent *actually* had a workplace accident or illness, it would not offer the basis for a useful indicator on its own. Rather, the only use for this would be to create an incidence matrix of exactly the kind I present here anyway, albeit one based on less data than is available in the Labour Force Survey. When we seek to measure health and safety in job quality indices, we are not interested in whether an incidence occurred for a given worker: rather we are interested in what the incidences tell us about *the risk* of accidents and injuries that any worker in the same or a similar job has to live with. I suggest that with the data limitations we are faced with, this is best done by creating an incidence matrix of the kind introduced in this paper.

D.4 Indicator creation

Unlike with other indicators in the QoW index, workplace health and safety data does not lend itself well to a cut-off approach. Similar considerations also apply to the Long-Term Prospects indicator (see Appendix E). This is because:

- There is no "acceptable" level of workplace accidents, illnesses or fatalities to assign a "Best" score: any rate above zero should be reflected in an indicator score.
- Unlike with other variables in the index where a basis for clear and rigid cut-off – be it the nature of the data, or some societally-agreed minimum standard or threshold – no such cut-off can be identified for health and safety.
- Any cut-off is highly sensitive to small changes in incidence rates. In some waves, these small changes can lead to a much more pronounced improvement/decline in workplace accidents than is apparent from the data itself.
- A clear argument can be made that any improvement or decline in incidence rates per 100,000 have an equal effect on wellbeing at any level of the distribution. This does not apply to other numerical indicators such as income, where principles such as declining marginal utility, and considerations about minimum thresholds, apply.

Because the incidence rates of accidents, illnesses and fatalities vary within each indicator, as noted at the start of this appendix, the indicators are created by standardising the scores in each indicator by turning them into Standard Units, and then converting them into a 0-2 scale to generate the scores.[15] These scores from each indicator are then added together into an equally-weighted Health and Safety dimension.

[14] Email exchange with the ONS Social Surveys team, 27 October 2023. Also confirmed in supplementary analysis, not presented here, of the characteristics of respondents vs. non-respondents.

[15] This approach is strongly justified for work fatalities, since there is surely widespread agreement that the lower incidence of work fatalities per 100,000 needs a higher weighting than could ever be captured by generating the scores based on a combined

The distribution of scores within each scale are contained in Table C.6. As can be seen, whilst there is a full range of scores for all indicators, the distribution of scores within each indicator is different. This is an intended feature of the standardisation, and helps ensure for example that the relatively low rate of work fatalities across most of the distribution (despite high fatalities amongst a minority) is reflected in the indicator.

distribution of all three indicators. It is more contestable for work accidents vs. work illnesses: although work illness rates are higher than work accidents, an argument could be made that in terms of severity both should be weighted equally. The approach taken in this paper effectively assumes this by generating the scores based on their own separate distributions, but an alternative approach could be justified pending further research on the relative severity of accidents vs. illnesses.

Appendix E – Long-Term Prospects indicator

Despite its other advantages for a job quality index, Understanding Society lacks objective data on the long-term prospects of workers' jobs. The Short-Term Prospects indicators, whilst useful, provide a more subjective assessment of workers' perceptions about their likelihood of finding a new or better job, obtaining employer-training or starting their own business, and over a very short timeframe. However, Understanding Society does contain ample data on the Standard Occupational Classifications (SOCs) of respondents' current jobs, with few missing values for SOC 2000s within the survey.[16] In this paper, these are used to generate a more objective indicator on long-term employment prospects over the decade 2017-2027 by SOC. By introducing this data into Understanding Society, we are able to not only follow trends in job prospects over time, but also look at differences in prospects by various sub-groups and based on various indicators in the QoW Index.

The process for introducing this data is similar to the Health and Safety dimension, but with some notable differences. It is also done solely using Understanding Society, without the use of the Labour Force Survey:

1. Data on long-term projections for every occupation is taken from the Department for Education's (DfE's) Working Futures surveys (DfE, 2020), the technical work for which is done by the Institute for Employment Research at the University of Warwick and Cambridge Econometrics (Wilson et al., 2020). This reports both (a) future employment projections and (b) replacement rates for every 4-digit SOC 2010 for the period 2017-2027. This means, unlike health and safety, the basis for the data is SOCs rather than SICs. The future employment projections are based on macroeconomic forecasts about the future growth of different sectors and occupations in the UK economy, and also consider the impact of technological change such as automation on different sectors. Alongside this, replacement rates consider how much of the workforce in each occupation is likely to leave the workforce in future years, such as due to retirement, which also of course creates demand for occupations in that sector.
2. Like with health and safety, the data is read into Understanding Society by first creating a matrix, this time for long-term employment prospects. The matrix is created by extracting data from Working Futures for every 4-digit SOC 2010 from the DfE's public API, accessible on their 'LMI for All' website (Barnes et al., 2021). In line with the latest data from Working Futures, these projections are for the period 2017-2027.
3. Data on future employment rates and replacement rates are then combined into a single indicator (see below subsection for a fuller description), which gives a projection of the future growth of that occupation for the period 2017-2027 as a percentage of the level of employment in that occupation in 2017.
4. The data are then read into Understanding Society. Because Understanding Society uses SOC 2000s, with less good coverage of SOC 2010s, this is done by first filtering specifically to the sample of respondents in the survey who have *both* 2000 *and* 2010 SOCs. Weighted projections of future growth by 2000 SOC are then generated to generate a matrix by 2000

[16] Understanding Society contains good data on the SOC 2000s of respondents. Missingness is low but gets progressively higher over the course of the survey, ranging from 1% of respondents in Wave 4 to 6% in Wave 12. These are contained in the variable jbsoc00. Respondents who change or find jobs during the course of Understanding Society are additionally coded based on the more up-to-date 2010 SOCs (jbsoc10). Missingness for SOC 2010 ranges from 57% of respondents in Wave 4 down to 25% in Wave 12. Because of this procedure, many respondents have SOC 2000s and SOC 2010s, as will be elaborated on later. This fact is crucial for the process I use to introduce them into Understanding Society.

SOC. The data from this matrix is then used to generate projections of future employment prospects by 2000 SOC.

5. Finally, this is turned into an indicator on Long-Term Prospects following a similar process as for the health and safety indicators. Scores are standardised by first turning the variable into standard units, and then converting the scores into a 0-2 scale.

E.1 Long-term Prospects

The DfE's Working Futures survey contains two useful projections for the future employment prospects of occupations for 2017-2027. These are used as the basis for the Long-Term Prospects indicator:

- **Employment change.** These estimate the future number of people employed in each occupation (including employees and self-employed) over the decade;
- **Replacement demand.** These estimate, in addition to employment growth, the future demand for workers in this occupation over the same period. Even an occupation with low-to-negative employment growth may have a high replacement demand, for example due to the occupation comprising older workers who are replacing retirement, or people leaving the occupation due to other commitments (eg family).

Consistent with the process used in Working Futures, these are converted into a combined indicator by simply adding the change in employment and replacement demand over the period together, as a percentage of the size of the occupation in 2017. It should be borne in mind that, for most occupations, the replacement demand is considerably higher than the employment change, and thus plays a predominant role in the indicator:

$$\textbf{Long Term Prospects} = \text{\% Employment change} + \text{\% Replacement demand}$$

Although the data is available in the API broken down by 4-digit SOC 2010, in practice the *percentage* projections for occupations within Working Futures are calculated at a higher level of aggregation of 2-digit SOC (see Wilson et al., 2020, p. 57), with only the projected *number* of workers employed in each occupation varying by 4-digit SOC. Table E.1 contains a matrix of employment prospects by the 25 2-digit SOC 2000 categories.

Although these data offer an improvement on the employment prospects data available in Understanding Society, it is important to be clear what the indicator is designed to measure. It gives an indication as to the future demand for the kind of occupation which the worker is doing: whether their current role, with the skills and attributes associated with it, is one which is likely to be "in demand" over the coming decades. A significant limitation of many existing indices of job quality is that they lack indicators of long-term employment prospects, despite extensive public discussion on work in the context of economic development and technological change.

However, the indicator has some limitations. An occupation with a high future demand may not necessarily be a "good job" for various other reasons related to the objective characteristics of a job itself. High replacement demand in an occupation, for example, may owe itself precisely to

Table E.1. Rank of Long-Term Prospects by 2-digit SOC 2000 in Understanding Society. Pooled data of respondents with both SOC 2000s and SOC 2010s.

SOC 2000	SOC 2000	Long-Term Prospects (% employment growth + % replacement rate p.a., 2007-2017)
61	Caring personal service occupations	5.89%
51	Skilled agricultural trades	5.75%
23	Teaching and research professionals	5.43%
22	Health professionals	5.39%
12	Managers and proprietors in agriculture and services	5.39%
24	Business and public service professionals	5.36%
32	Health and social care associate professionals	5.28%
34	Culture, media and sports occupations	4.93%
35	Business and public service associate professionals	4.85%
11	Corporate managers	4.79%
72	Customer service occupations	4.73%
62	Leisure and other personal service occupations	4.50%
82	Transport and mobile machine drivers and operatives	4.31%
92	Elementary administration and service occupations	4.06%
21	Science and technology professionals	3.93%
53	Skilled construction and building trades	3.34%
91	Elementary trades, plant and storage related occupations	3.31%
31	Science and technology associate professionals	3.29%
41	Administrative occupations	3.24%
71	Sales occupations	2.93%
33	Protective service occupations	2.20%
54	Textiles, printing and other skilled trades	2.02%
52	Skilled metal and electrical trades	1.27%
81	Process, plant and machine operatives	0.36%
42	Secretarial and related occupations	-4.83%

some of these characteristics. An occupation with good prospects may have low pay and few-to-no promotion opportunities within the occupation in which the worker is working. On its own, the indicator is no substitute for a fuller assessment of the *quality* of the job being done. However, this is precisely the purpose of including it within a wider QoW index: the inclusion of additional indicators of job quality allows us to explore the relationship between prospects and job quality in much more detail than has been possible previously, allowing us to account for these issues by including them in a more holistic index.

Another feature to bear in mind is that, by its own definition, there should be at least some improvement in this indicator over time. This is because the indicator is designed to capture the future change in occupational structure, so any sufficiently representative survey should capture this by showing fewer people employed in the low-prospect occupations and more employed in the high-prospect occupations. This does not, on its own, mean this indicator should not be included in an index of job quality, because there may be significant differences *within* societies in which workers are sorted into occupations with low vs. high prospects. However, it is important to bear this in mind when exploring trends in this indicator.

E.2 Robustness and representativeness

There are some inherent challenges in comparing representativeness of this data in the same way as with the health and safety indicators. This is because for the period under study, the LFS does not contain a variable for 2000 SOCs like Understanding Society: the LFS switches to updated SOCs in Q1 of the year following an update, meaning it has 2010 SOCs from Q1 2011 to Q4 2020. Only a more imprecise comparison of the two surveys is possible, by comparing prospects using SOC 2000s in Understanding Society with SOC 2010s in the Labour Force Survey. In addition to differences which could arise from SOC 2000s, differences could also arise as a result of similar issues as for the health and safety indicators – such as an over- or under-representation of certain occupational groups in one survey vs. another.

Despite these considerations, Figure E.1 shows that both Understanding Society and the LFS report a very similar rate of long-term prospects, measured here as a time series of the weighted mean annualised growth in employment plus replacement rate for each SOC. Understanding Society tends to report a slightly higher rate of growth, although the trends in both surveys are the same, showing a slight rise in the long-term prospects of jobs over the course of the time series (for reasons discussed in the preceding section, a slight rise is to be expected given the nature of the indicator). Additional supplementary analysis by sub-group, not presented here, also shows similar results between both surveys, despite the difference in SOCs used.[17]

Finally, some alternative approaches to constructing this indicator were explored and discounted. These are discussed below:

- Consideration was given to introducing this data into Understanding Society through the LFS in a similar way to the health and safety dimension. However, as noted above, the LFS contains SOC 2010s, and not SOC 2000s, for almost all of the period studied (Q1 2011- Q4 2020). Unlike Understanding Society, the LFS also does not run different SOCs concurrently in the same survey, meaning it is impossible to compare two different groups of SOCs using the LFS. The alternative approach of introducing the data into

[17] For example, both surveys show a higher level of prospects for women, for self-employed workers, and for London vs. other regions of the UK. Differences in prospects by ethnicity and parental NS-SEC are mixed. Overall, there is much less inequality in this indicator by sub-group than there is in other indicators of the QoW index. This is reflected in the distribution indicator scores discussed in the next subsection.

Understanding Society based on SIC 2007s was implemented and investigated. Whilst this had broadly similar trends over time and differences between sub-groups as introducing the data using SOC 2000s, it showed markedly lower levels of variance in job prospects between different individuals and different industrial classifications. This is likely owing to the diversity of occupations within each industry, effectively averaging out differences within various industrial groups. This approach was therefore rejected.

- An exploration was also undertaken into the possibility of updating 2000 SOCs into 2010 SOCs. This was rejected as unfeasible. Whilst the ONS does publish a list of 2000 SOCs by 2010 SOCs, in practice it is not possible to update SOCs or to downgrade them without introducing considerable subjectivity and bias into the data. This is because some occupations which have one SOC in 2000 will split into two SOCs in 2010, and vice-versa.[18] Initial analysis, not presented here, found that this affects a far from trivial number of occupations, including a great deal of occupations in the health sector which represent a large number of workers. Analysis by the ONS into the relationship between *2020* and 2010 SOCs further reinforces this (ONS, 2021).
- Finally, an alternative, mixed approach was also considered for introducing the data into Understanding Society: using 2010 SOCs where these were available for respondents and using the 2000 SOC matrix where these were not available. This was rejected as introducing too much bias into the results, since those with 2010 SOCs will by definition be respondents who changed jobs during the course of the survey. Rather, bearing all the above consideration in mind, and given the limitations of the data, the most reasonable approach is deemed to be the one taken here: making an assessment of the prospects of each SOC 2000 based on the sample of respondents in Understanding Society with both 2000 and 2010 SOCs.

E.3 Indicator creation

Similar considerations as for the health and safety indicators apply to indicator creation for Long-Term Prospects. As with those indicators, it is for example difficult to identify an appropriate cut-off based on employment prospects. The scores are less sensitive to minor variation from a cut-off approach in the same way as the health and safety indictor, but such an approach would still give unwarranted weight to scores either side of any given cut-off: it is more true to say that a difference in prospects likely to have a consistent effect on job quality at any level of the distribution.

To generate the indicator, scores are therefore turned into standard units and then converted into a 0-2 scale. The indicator is added as a second indicator into the Prospects dimension, and is given equal weighting alongside the Managerial Duties indicator. Table E.2 contains the distribution of scores for Long-Term Prospects. It shows that whilst there is a full range of prospects scores, there is a skewness towards the higher end of the distribution. This is consistent with the prospects matrix presented in Table E.1.

[18] Note that this is the case for SOCs at every level of granularity: it applies to 2-, 3- and 4-digit SOCs, and not merely for just the lattermost. Even though there are 25 2-digit SOCs in the 2000 and 2010 SOCs, the allocation of workers within these 2-digit categories does change. Sometimes one 2-digit occupational code is split across two codes in the new survey, and conversely sometimes a single 2-digit code in the 2010 SOCs is associated with 2 different codes in the 2000 SOCs.

Table E.2. Comparison of the distribution of indicator scores for long term prospects. All waves.

Indicator	Min.	1st Quartile	Median	Mean	3rd Quartile	Max.
**Long-term Prospects**	0	1.505	1.784	1.657	1.887	2

Figure E.1. Time series comparison of weighted mean long term prospects (change in employment + replacement demand p.a for each SOC) in Understanding Society (SOC 2000s) vs. the Labour Force Survey (SOC 2010s), 2012-13 to 2020-21.

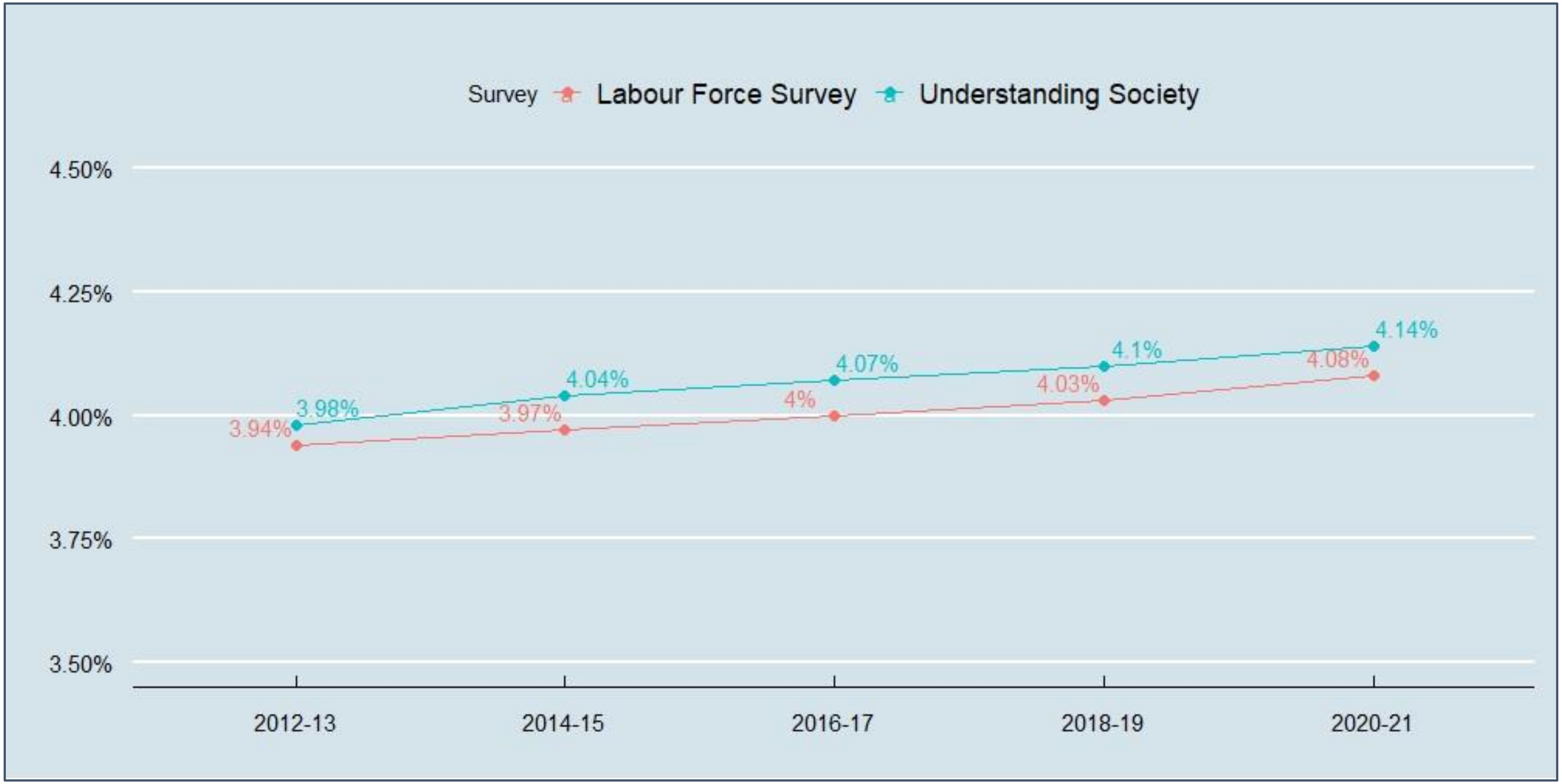

Appendix F – Further discussion of QoW indicators and dimensions

I conclude with an overview of each of the indicators of the QoW index. Using the Capability Approach, I discuss some of the normative decisions underlying the choice of indicator selection and the calculation process for indicator scores. I also discuss some limitations of the indicators, particularly based on data availability; some alternative calculation and indicator selection approaches; and the comparability of the data with other published national statistics, to test the representativeness of Understanding Society data.

The QoW index groups the indicators into 7 dimensions. I define these as groupings of work characteristics based on the way that they impact similar groups of Functionings. These indicators are usually therefore positively correlated with each other, but it is not a requirement for them to be in order to be included in the same dimension. For example, as briefly discussed in the paper, within the Work-life Balance dimension, there is only a weak correlation between Excessive Hours and Employee Flexibility. As discussed in the paper, may reflect the fact that workers can reconcile work-family and family-work conflict by reducing their hours *or* accessing flexible work opportunities: these are different means of achieving the same Functionings. This should be borne in mind in the succeeding subsections.

F.1 Earnings

Even though job quality research has tended to emphasise the role of non-pecuniary aspects of work, there is still widespread recognition across the social sciences that earnings play a critical role in job quality. This perhaps distinguishes it from other areas of wellbeing research, such as the multidimensional poverty agenda.(Cazes et al., 2016) I suggest that people achieve Functionings from earnings in two distinct and separate ways. Often only the first of these is discussed in literature:

- We achieve some Functionings based on where their wage is within the distribution, not least because this is a signifier of the status and worth society attaches to their job (Earnings Equity). For this, we tend to use *gross* hourly wages: how does our pro rata hourly wage or salary compare with others?
- Distinct from this, we also need our earnings to be sufficient to achieve, and ideally far exceed, the level of Functioning achievement to enjoy a societally-agreed minimum acceptable standard of living (Earnings Sufficiency). We need money to pay for many of these Functionings. Because of this, this is best seen in terms of *net* earnings: what is our actual take-home pay – after accounting for hours worked, pay deductions, etc – and what can we buy with this money?

Earnings Equity is the easier of the two concepts to operationalise. There is widespread agreement that there is declining marginal utility to higher wages. In terms of the Capability Approach, this can be conceptualised in terms of imagining expect diminishing returns to Functioning achievement the higher up the gross wage distribution you go: you may be deeply concerned if you are very low down in the wage distribution, but not concerned if you are in the top end of the distribution. To capture this, I use a categorical indicator which calculates whether gross hourly wages5 are (a) below the 20th (Worst), (b) at or below the 60th (Middle), or (c) above the 60th (Best) percentile of the distribution. This is informed by an approach taken in existing studies of wage inequality (Lindley and Machin, 2013; Machin, 2011), which tend to place an emphasis on trends in the bottom fifth of the wage distribution vs. other parts of the wage distribution. To assess potential changes over future years, the percentile thresholds are set in standard units at Wave 4. As can be observed, this means that exactly 20% of people score Worst, 40% score Middle and 40% score Best as at Wave 4; this is an intentional feature of the indicator. Changes from this in future years mean that, in aggregate, the relative position of workers has

improved. It does not however necessarily mean that this improvement has necessarily accrued to the workers who scored Worst in an earlier wave.

In this index, Earnings Sufficiency is operationalised using a categorical indicator: assessing whether net monthly earnings, irrespective of hours worked, are below the Joseph Rowntree Foundation's Minimum Income Standards (MIS) for (a) a single person with no dependents (Worst), (b) half the MIS of a dual earner couple, including childcare costs (Middle) or (c) above both thresholds (Best) (Bradshaw et al., 2008; Hirsch, 2015). These standards have been developed and updated through a deliberative process of public engagement, in which people were asked to agree minimum baskets of goods necessary to participate in society – akin to similar processes using the Capability Approach. The cut-offs effectively tell us whether someone has the Capability, through earnings alone, to enjoy a minimum societally-agreed standard to live alone, or have two children as part of a dual-earning couple.

It could be argued that the Earnings Sufficiency indicator should set a different threshold based on wages rather than the Minimum Income Standard – such as the Living Wage Foundation's living wage and London living wage rates. This study rejects the use of indicator cut-offs based on LW/LLW thresholds for normative reasons:

- The LW/LLW rates assume 100% take-up of any welfare benefits individuals are eligible for. This means that whilst the rates are an improvement on the Government's National Living Wage, the thresholds are still insufficient for someone to enjoy a minimum standard of wellbeing from work alone.
- The above discrepancy has the effect of making changes in the time series sensitive to changes in welfare provision, rather than any underlying change in job quality.
- The process by which the thresholds account for costs associated with other household members is unclear. It is therefore not clear whether the wage is designed to be sufficient for eg a person with children, and if so how many children; whether childcare costs are included; whether another household member contributes to these costs, etc.

The MIS thresholds do not have these three issues. No assumptions about welfare benefits receipt are made: they are designed to be income thresholds which need to be met in order to enjoy a decent standard of living. This means it is possible to establish whether someone is able to meet these standards from earnings alone, simply by comparing net earnings to the weekly thresholds. This should not be misinterpreted as making any normative statement about whether individuals should be expected to secure their wellbeing from work alone. Rather, this process ensures that the QoW index measures what it is expressly designed to measure: it means trends in Earnings Equity are less sensitive to extraneous factors which wouldn't reflect an underlying change in job quality. The MIS thresholds are also very explicit about the household costs they capture, making a distinction between costs for a range of sub-groups. This means it is possible to use these thresholds to establish whether an individual has the Capability to exercise these family-related Functionings, whether on their own or as part of a dual-earning couple.

The data from both these indicators is consistent with other published statistics. The UK has indeed seen a large fall in the share of low paid workers in recent decades, which is reflected in the trends in Earnings Equity. Because the Earnings Sufficiency indicator is more novel, the uncovered trends are harder to compare with other data sources, but they are consistent with trends in hours worked and self-employed earnings which would explain the trends observed. Overall, they are consistent with the discussions of Understanding Society income data in other studies, which find it compares well with other sources – such as the Annual Survey of Hours and Earnings – and has the added advantage of including crucial data on self-employed earnings.

F.2 Insurance

Work plays a key role in insuring people against risks both during work and, crucially, in their future lives through pensions. Although pensions policy is generally conceptualised in terms of equalising and smoothing consumption (Barr, 2020, pp. 157–192), it can also be framed in terms of the equitable distribution of Functionings and Capabilities within and between generations. In the UK, good-quality paid work has been vital to the funding of pensions in two respects: earnings taxes pay for state-provided defined benefit pensions; and employer and employee pension contributions pay for personal pensions (either defined benefit or, increasingly in the UK, defined contribution). There is concern that low QoW – particularly rising informality and wage stagnation – will undermine both these foundations (Barr and Diamond, 2010). Many existing job quality indices lack a pensions dimension, but these considerations make the exclusion of them in the UK context particularly untenable. Hence its high weighting in the QoW index.

To capture this, the index uses two groupings of questions from Understanding Society. First, a set of employee-only questions ask respondents whether their employer has an employee pension scheme, and if so, whether they are a member. Second, a set of questions asked to all paid workers on whether they contribute to a personal pension, and if so, how regularly. These are used to develop a categorical indicator combining employees and self-employed. Employees are assigned the Best score if they are members of their employee pension scheme. The self-employed are assigned a Middle score if they contribute regularly to a personal pension, and employees are assigned a Middle score if they don't have a workplace pension but nonetheless contribute regularly to a personal pension. The trends in the Insurance indicator serve as a validation of the representativeness of Understanding Society data: they align with ONS data which show a sharp rise in the proportion of employees covered by workplace pensions following the introduction of Automatic Enrolment (ONS, 2022). The self-employed, who only have recourse to personal pensions, have seen no such improvement.

It would be possible to devise an alternative Pension indicator which treats the self-employed more generously, for example by only assigning them a Best if they contribute to a personal pension. This would not affect the overall conclusions of this paper, since the data already shows sharp differences in the Insurance dimension for employees and the self-employed, which widen over the time series with the implementation of automatic enrolment – it would only serve to slightly reduce these differences. Nonetheless, this approach was rejected for normative reasons. Whilst it may be reasonable to assume that some self-employed workers have pensions which are better quality than employees, in practice employers do not contribute to most personal pensions – depriving the self-employed, and employees without workplace pensions, of crucial opportunities to supplement their pension savings. Nonetheless, a more generous approach would not change the observed *trends* discussed in the paper; only the relative position of sub-groups would change.

Whilst the Pensions indicator is an improvement on other job quality indices, most of which do not have such an indicator, there is still scope for further refinements. Understanding Society does not contain data on the size of the pension pots of respondents, so it is not possible to establish whether respondents who belong to employer schemes have saved enough to enjoy their retirement. Conversely, older respondents with access to a pension may choose not to contribute because they already have sufficient retirement earnings: indeed, people aged over 66 in the QoW index do in fact score significantly worse on the Insurance dimension. Finally, it is not always clear whether Understanding Society's derived net earnings variables deduct for earnings related to employee pension contributions, since this would depend on how the worker reports the income from their payslip (which would usually deduct for pension costs). These could be non-trivial sums for many workers enrolled onto employee pensions for the first time, reducing their

real wages below the MIS thresholds. The data also does not allow us to establish the size of the contributions, the nature of the pension (eg defined benefit such as final salary vs. defined contribution), or the expected income in retirement, but the indicator still marks an improvement on existing job quality indices, most of which do not use a pensions indicator. There is the potential for future research should explore ways of addressing these limitations by making use of a wider range of indicators in Understanding Society, and potentially introducing data from other datasets and pension schemes.

F.3 Security

Security, which is used here as an antonym of precarity, is widely agreed to be a key dimension of job quality. Sociological literature has identified "insecure and uncertain" work to be one of three inter-related aspects of precarious work, alongside "limited economic and social benefits" and "limited statutory entitlements" (Kalleberg, 2018, p. 15). Conceptualised using the Capability Approach, precarious work could be argued to affect Functionings inside the space of work such as meaningful work, since it signifies to the worker that their work is less worthwhile to employers and society. It also has a considerable effect on the ability of the worker to exercise Functionings outside the space of work: offering less secure earnings, and preventing the worker from planning for the future to exercise family- and life-related Functionings. As discussed in the paper, the concept has become increasingly relevant since the 1980s in the context of significant changes in labour markets in the Global North and the continued predominance of insecure work in the Global South (e.g. see Burchell et al., 2002; Gallie, 2004, 2003).

The Earnings Sufficiency indicator in the QoW index already captures aspects of precarity related to low hours and/or low wages: someone with a high wage but unable to work sufficient hours, or conversely someone working long hours at an insufficient wage, could fall fellow the MIS thresholds. The Security dimension contains two other indicators designed to capture other aspects.

First, Continuous Employment uses longitudinal data from both the current and prior wave of Understanding Society to generate a categorical indicator based on length of continuous service with the same employer. This is an especially important indicator in the UK context, since employees' statutory rights, such as unfair dismissal, depend on the length of continuous service. A distinction is drawn between employees with (a) < 1 wave (Worst score), (b) 1-2 waves (Middle) and (c) > 2 waves' (Best) continuous service. By definition, the self-employed and those who were out of work 1-2 waves ago do not have associated legal rights based on continuous service. The self-employed are therefore assigned the Worst score, and those who were out of work in the relevant period are assigned the corresponding (Middle or Worst) score. In line with Kalleberg's (2018) framework, this indicator therefore incorporates a wealth of data on non-standard work arrangements, precarity and insecurity whilst also being sensitive to the specific legal framework and level of worker power in the UK context. It should also be noted that the key question used to create this variable (jbsamr) is asked in such a way as to match the UK's legal framework for continuous employment: it specifically refers to having "worked continuously for the same employer", and an additional prompt in the questionnaire specifically advises interviewers to code workers who have been transferred to another employer under TUPE arrangements as continuously employed.

Second, Composite Security is a binary indicator which captures (a) whether the job is permanent or temporary (eg fixed-term contracts, seasonal work, etc) and (b) whether the worker perceives it likely/very likely to lose their job in the next 12 months. Employees are coded Worst if they answer yes to either question, whilst the self-employed – since they are not asked question (b) – are assigned scores based only on question (a). An alternative indicator of Continuous Employment could be developed focussing entirely on prior spells out of paid employment – such as whether the respondent was unemployed or inactive in the previous wave. This would be in

line with an indicator used in another application of the Alkire-Foster method to measuring job quality (e.g. see González et al., 2021). This approach is rejected in this paper, since it is possible to take advantage of the richness of Understanding Society data to create a more comprehensive indicator. The indicator already captures any individuals who were not in paid employment in the prior wave and/or the wave prior to that, since these people will by definition have less than 1 or 2 waves of continuous employment. However, in addition to this, it also captures (a) anyone self-employed, since by definition they lack the statutory rights associated with continuous employment, thus scoring Worst; and (b) any employees who, despite being continuously employed, have fewer than 1 or 2 waves' continuous service in their current job.

Some limitations of these indicators warrant mention. It is difficult to find comparable data for Continuous Employment, since Understanding Society has the advantage of (a) surveying all paid workers (employees and self-employed); (b) following those out of the labour force in-between waves; and (c) interviewing workers directly rather than through employers. ASHE, by contrast, is an employer survey of employees only. ASHE data suggests that mean job tenure for many workers is very long, standing at 9.8 years in the public sector and 6.7 years in the private sector (ONS, 2017). Whilst this would appear to be broadly consistent with the uncensored headcount ratios in the QoW index (once filtered to employees only), they are not easily comparable, and any discrepancy could also reflect the different nature of the two populations and survey methods as noted above. For Composite Security, the data suggest a markedly better picture than what information on employees' actual contracts would suggest it should be. Indeed, even a majority of self-employed workers report having permanent jobs in this indicator (albeit still markedly lower than employees). This is likely due to question ordering in Understanding Society data: workers are first asked whether their current job is permanent or temporary, and if they say it is temporary, they are then only afterwards prompted for the ways it is not permanent – such as fixed-term contracts, seasonal work, platform labour in the gig economy, etc. Whilst this issue does not affect the overall conclusions of this paper, it is suggested that future indices could arrive at a more objective measure of job insecurity by asking workers a set of binary questions about the existence of specific contractual arrangements *first*. This would likely give a more reasonable picture of the real level of insecure working arrangements in the UK labour market.

F.4 Autonomy and Voice

This dimension captures two more detailed aspects of the working environment: workers' autonomy, and their power to exercise collective voice to shape the way they work. Autonomy refers the level of initiative workers have over their tasks, and is recognised across different disciplines, and across a wide spectrum of different philosophical approaches to work and wellbeing, to be a central part of the employment relationship (eg see Gallie 2007). It is associated with work intensity, defined as "the rate of physical and/or mental input to work tasks performed during the working day" (Green, 2001, p. 54), and evidence suggests that since the late 20th Century task autonomy has declined in Britain (Gallie et al., 2004) just as work intensity has increased (Green et al., 2022).

The separate concept of voice has its origins in economic literature studying the options available to consumers faced with declining quality of goods and services (Hirschman, 1970), but subsequent work applied this framework for the study of job quality. Such literature traditionally associated voice with unions due to their unparalleled ability to exercise collective voice (Bennett and Kaufman, 2007; Boroff and Lewin, 1997; Freeman and Medoff, 1992), but the decline of unions has seen the growth of a much broader range of definitions and mechanisms for voice (Budd et al., 2010). A separate strand of literature in the Capability Approach has argued for a Capability for Voice (Bonvin, 2012; De Leonardis et al., 2012; De Munck and Ferreras, 2013); the way both schools of thought conceptualise voice has yet to be reconciled.

The QoW index incorporates these concepts into two indicators.

First, Autonomy combines five variables, which ask all workers to assess the level of autonomy over five aspects of work on a 4-level scale from “a lot” (1), to “some” (2), “a little” (3) and “none” (4). Summing these together leads to 16 possible scores, ranging from 5 (“a lot” of autonomy in all five variables) to 20 (“none” in all five). A categorical indicator is created, distinguishing between scores of (a) 5-9 (Best), 10-15 (Middle) and 16-20 (Worst). Because the number of potential Autonomy scores is not divisible by 3, the Autonomy indicator assigns the Middle category an extra score. One of the most significant limitations of Understanding Society is the lack of a question on work intensity, but autonomy is argued to be sufficiently theoretically and empirically associated with intensity for the reasons described above. The work arrangements summed up are as follows:

- Autonomy over job tasks (wkaut1)
- Autonomy over work pace (wkaut2)
- Autonomy over work manner (wkaut3)
- Autonomy over task order (wkaut4)
- Autonomy over work hours (wkaut5)

Second, adopting the conceptualisation of voice taken in more traditional literature, the index uses a binary Collective Voice indicator which distinguishes between employees who (a) have (Best score) or (b) do not have (Worst) “a trade union, or a similar body such as a staff association, recognised by your management for negotiating pay or conditions for the people doing your sort of job in your workplace.” The self-employed are automatically coded Worst on this indicator, since they are not asked this question and, by definition, they are denied access to an employer-recognised means of exercising collective voice. There is no data in Understanding Society on potential wider avenues for exercising worker voice, or indeed of worker perceptions of how their voice can be exercised in the workplace.

For the Collective Voice indicator, there is a recognised discrepancy in the level of union and collective bargaining coverage across different national surveys, and no clear consensus exists about which survey represents the true levels (BEIS, 2022). Due to discrepancies in question ordering and wording between the LFS and Understanding Society, Understanding Society tends to over-estimate collective representation (by prompting for “staff associations” as well as unions in the question) and under-estimate union membership (by only asking those who report collective agreements in the workplace about their union membership). As a result of this, the Collective Voice indicator in the QoW index should be interpreted conservatively: it should not be interpreted as suggesting the existence of a formal collective bargaining arrangement for all respondents. Rather, it is designed to capture the existence of some collective means through which workers can exercise their voice in the workplace.

F.5 Work-life Balance

Considerable multi-disciplinary research has studied work-family and family-work conflict (Annor and Burchell, 2018; Chung and van der Lippe, 2020; Epstein et al., 1999; Esping-Andersen, 1996; Gallie, 2007; Parasuraman and Simmers, 2001) – including within the Capability Approach (Hobson, 2011; Lewis and Giullari, 2005). This paper conceptualises this as an inability to exercise both work-related and family-related Functionings at the same time. Workers facing this conflict therefore face a choice between (a) reducing their work activity to exercise family-related Functionings, eg by sacrificing pay or other aspects of job quality (eg career prospects); or (b) holding off from exercising family-related Functionings. Good-quality work enables workers to

exercise both sets of Functionings simultaneously. A range of work resources are important in enabling or preventing this from happening – including flexible working arrangements, earnings, and the sheer quantum of time spent in work (since excessive working hours will lead to time poverty, by definition preventing workers from exercising other Functionings).

The Earnings Sufficiency indicator is partly designed to capture some aspects of work-life balance, since someone forced through family or caring responsibilities to work insufficient hours to achieve a decent standard of living would score poorly on this indicator. Two categorical indicators are designed to capture wider aspects of this issue.

First, Employee Flexibility utilises an indicator which asks employees a set of yes/no questions about the availability of numerous flexible work arrangements in their workplace. Due to the heavily left-skewed nature of this data, the indicator assigns a greater weight towards having more flexible arrangements at the lower end of the distribution, distinguishing between (a) zero flexible working arrangements (Worst score), (b) one or two flexible work arrangements (Middle) and (c) three or more flexible work arrangements (Best). I construct the indicator based on the availability of 8 worker-oriented flexible work arrangements. Note that for any of these arrangements to count towards the indicator score, they simply need to be *available* in the workplace; the worker does not have to actually use them:

- Part-time working (jbflex1)
- Working term-time only (jbflex2)
- Job sharing (jbflex3)
- Flexi-time (jbflex4)
- Working a compressed week (jbflex5)
- Working annualised hours (jbflex6)
- Working from home on a regular basis (jbflex7)
- Other flexible work arrangements (jbflex8)

As of Wave 12 two additional work arrangements of a zero-hours contract and on-call working are also available, but these are excluded from the indicator because they are not asked before Wave 8 and in any event they do not in fact reflect genuine worker-oriented flexible work arrangements.

Second, Excessive Hours uses data on weekly hours worked in all jobs to capture aspects of work-life balance associated with excessive working hours. It distinguishes between those who work (a) over the UK Working Time Directive of 48 hours a week (Worst score), (b) over 37 hours (Middle) (c) 37 hours or below (Best). The Middle cut-off is informed by the distribution of hours worked and the average weekly working hours of full-time workers, which currently stands at 36.7 hours (ONS, 2024). Because the self-employed are not asked about flexible work arrangements, they are only coded based on the Excessive Hours indicator. Note that legally self-employed workers are not subject to the Working Time Directive, but it justified to still score them Worst if they exceed it.

The Excessive Hours data closely corresponds with published data in the Labour Force Survey. For example a 2014 study by the then- Department for Business, Innovation and Skills into the impact of the UK's Working Time Regulations found a similar proportion of employees (13%-15%) worked over 48 hours in 2010-2013 (BIS, 2014, p. 32). LFS data also shows a similar polarised distribution of self-employed hours worked, with a higher proportion working excessive and low

hours and a lower proportion in the middle of the distribution (BIS, 2014, pp. 27, 29). The higher incidence of excessive hours partly reflects the fact that as noted above, the UK Working Time Directive requirement to opt-out of a 48-hour working week only applies to employees, and not to self-employed. The higher incidence of low hours reflects the fact that newly self-employed appear to have poor work histories, and thus are likely accessing what little self-employed work they can given their poor work opportunities.

F.6 Prospects

The prospects of jobs tend to be discussed from the perspective of employers or national economies rather than workers themselves – for example in discussions of skill-biased technical change, human capital and economic productivity. However, I suggest that job prospects can be conceptualised from the workers' perspective. Jobs with good prospects are more resilient to future changes, eg by using in-demand skills such as jobs in the green economy; offer good promotion opportunities; and are likely further up the hierarchy and so less vulnerable to sudden lay-offs and restructuring. They provide workers with Functionings inside and outside the space of work: they are more likely to be meaningful, whilst also being a more stable and certain means of obtaining other Functionings. Where skills are measured, it is important to distinguish the skill of the job from the qualifications of the individual.

This is captured in the QoW index using one binary indicator, a categorical indicator and a continuous indicator.

Firstly, Managerial Duties captures whether the worker is either solo-self-employed (if self-employed) or has managerial duties (if an employee). Non-managerial employees and solo self-employed are coded Worst, and the converse coded Best. This is in line with literature suggesting those self-employed who hire staff have significantly better pay and prospects (Giupponi and Xu, 2020): by definition, they can lay other workers off first if they face a reduction in their revenue. The data on Managerial Duties serves as a further validation of the representativeness of Understanding Society data, since it shows an increase in the proportion of solo-self employed within the self-employed population. This is in line with national labour market statistics, which show that the rise in self-employment in the UK over recent decades has been led entirely by solo self-employment.

The second, Short-Term Prospects, combines data from a set of question asked to workers about what they expect from their job, and out of paid work more generally, over the next 12 months. A total of five questions are asked in the survey, but the fifth (give up paid work) is not used to construct the indicator since it reflects a negative rather than a positive labour market event:

- Expects a better job with the same employer (jbxpcha)
- Expects work-related training (jbxpchb)
- Expects a new job with a new employer (jbxpchc)
- Expects to start own business (jbxpchd)

Similarly to Employee Flexibility, the distribution of scores for the combined prospects indicator are heavily left-skewed. This makes intuitive sense, since it is obviously unlikely that a worker would expect to achieve all of these things simultaneously over the course of 12 months. I therefore create a categorical indicator, splitting workers between (a) workers who expect none (Worst), (b) workers who expect one (Middle) and (c) workers who expect two or more (Best).

Third, as discussed in Appendix E, a more objective indicator of Long-Term Prospects over the next decade is also included in the index. As discussed, this is designed to capture aspects of job prospects which the Short-Term Prospects indicator does not measure: covering a longer

timeframe (2017-2027), and assessing the vulnerability of the worker's occupation to technological change, the replacement demand for jobs in their occupation, and the future economic projections about the sector they work in.

Taken together, these three indicators provide a broad spectrum of information about the job prospects of workers in the UK. Many job quality indices lack sufficient data on job prospects, but the QoW index redresses this through the development of dimension. Nevertheless, there is scope to refine this data in future research. The timeframe for the Short-Term Prospects indicator is far too short – just 12 months – and the question wording does not allow the researcher to delve into some specific details, such as their general expectations of being promoted in the job they work in. There is also a tendency for the vast majority of workers to score very high on Long-term Prospects. This reflects its genuine distribution in the data, with only a small proportion of occupations projected to see a decline in the next decade, with most other occupations expected to grow to a comparatively similar degree. However future updates and the introduction of more detailed labour market data into Understanding Society may be able to provide more granular detail, further building on this index.

F.7 Health and Safety

As discussed in Appendix D, workplace health and safety is a crucial measure of working conditions and features in a number of indices of job quality (e.g. Muñoz de Bustillo et al., 2011). Whilst early research focussed on more extreme risks such as environmental and chemical exposure, later literature has measured broader aspects of workplace health and safety such as risk of chronic health conditions caused by repetitive work tasks or sedentary working conditions. The measurement of the mental health risks associated with certain kinds of work is, however, still under-developed (Descatha et al., 2022). Due to lack of data, however, many job quality indices lack dimensions on health and safety. This is indeed an issue with Understanding Society, which as mentioned previously does not ask respondents any direct questions about their exposure to health and safety issues in the workplace.

This paper makes a significant contribution towards addressing this issue, with the introduction of three continuous indicators the QoW index: Work Fatalities, Work Accidents and Work Illness. In an improvement on some other indices, there is an inclusion of mental health: where a worker perceives this to have been caused or made worse by their employer, this would be reflected in incidence rates of work illness, so an industry more exposed to mental health risks would see this reflected in the Work Illness scores of workers in that industry. Further discussion of the indicators, comparison with the Labour Force Survey, and the process for assigning scores can be found in Appendix D.

References

Alvarez, P.C., James, N., Lynn, P., 2023. Panel attrition in the General Population Sample and the Immigrant and Ethnic Minority Boost of Understanding Society (No. 2023– 03), Understanding Society Working Paper. Institute for Social and Economic Research, University of Essex, Essex, UK.

Annor, F., Burchell, B., 2018. A cross-national comparative study of work demands/support, work-to-family conflict and job outcomes: Ghana versus the United Kingdom. Int. J. Cross Cult. Manag. 18, 53–72. https://doi.org/10.1177/1470595817746195

Azur, M.J., Stuart, E.A., Frangakis, C., Leaf, P.J., 2011. Multiple imputation by chained equations: what is it and how does it work? Int. J. Methods Psychiatr. Res. 20, 40–49. https://doi.org/10.1002/mpr.329

Barnes, S.A., Bimrose, J., Cárdenas-Rubio, J., Wilson, R., Owen, D., Hogarth, T., Bosworth, L., Day, R., Attwell, G., Rustemeier, P., 2021. Enhancing a labour market information database: LMI for All. Department for Education, London, UK.

Barr, N.A., 2020. The economics of the welfare state, Sixth Edition. ed. Oxford University Press, Oxford, UK.

Barr, N.A., Diamond, P.A., 2010. Pension reform: a short guide. Oxford University Press, New York, USA.

BEIS, 2022. Trade union membership, UK 1995-2021: statistical bulletin. Department for Business, Energy and Industrial Strategy, London, UK.

Bennett, J.T., Kaufman, B.E. (Eds.), 2007. What do unions do? a twenty-year perspective. Transaction Publishers, New Brunswick, NJ, USA.

Benzeval, M., Bollinger, C.R., Burton, J., Crossley, T.F., Lynn, P., 2020. The representativeness of Understanding Society (2020 - 08), Understanding Society Working Paper. Institute for Social and Economic Research, University of Essex, Essex, UK.

BIS, 2014. The Impact of the Working Time Regulations on the UK labour market: A review of evidence (No. BIS Analysis Paper Number 5). Department for Business, Innovation and Skills, London, UK.

Boelhouwer, J., 2002. Quality of Life and Living Conditions in the Netherlands. Soc. Indic. Res. 58, 115–140. https://doi.org/10.1007/0-306-47513-8_6

Bonvin, J.-M., 2012. Individual working lives and collective action. An introduction to capability for work and capability for voice. Transf. Eur. Rev. Labour Res. 18, 9–18. https://doi.org/10.1177/1024258911431046

Boroff, K.E., Lewin, D., 1997. Loyalty, Voice, and Intent to Exit a Union Firm: A Conceptual and Empirical Analysis. Ind. Labor Relat. Rev. 51, 50–63. https://doi.org/10.2307/2525034

Bradshaw, J., Middleton, S., Davis, A., Oldfield, N., Smith, N., Cusworth, L., Williams, J., 2008. A minimum income standard for Britain: What people think. Joseph Rowntree Foundation, York, UK.

Budd, J.W., Gollan, P.J., Wilkinson, A., 2010. New approaches to employee voice and participation in organizations. Hum. Relat. 63, 303–310. https://doi.org/10.1177/0018726709348938

Burchardt, T., Vizard, P., 2011. 'Operationalizing' the capability approach as a basis for equality and human rights monitoring in twenty-first century Britain. J. Hum. Dev. Capab. 12, 91–119. https://doi.org/10.1080/19452829.2011.541790

Burchell, B., Ladipo, D., Wilkinson, F. (Eds.), 2002. Job insecurity and work intensification, Routledge studies in employment relations. Routledge, London, UK.

Cascales Mira, M., 2021. New Model for Measuring Job Quality: Developing an European Intrinsic Job Quality Index (EIJQI). Soc. Indic. Res. 155, 625–645. https://doi.org/10.1007/s11205-021-02615-9

Cerioli, A., Zani, S., 1990. A Fuzzy Approach to the Measurement of Poverty, in: Dagum, C., Zenga, M. (Eds.), Income and Wealth Distribution, Inequality and Poverty: Studies in Contemporary Economics. Springer, Verlag, Berlin, pp. 272–84.
Cheli, B., Lemmi, A., 1995. Totally' Fuzzy and Relative Approach to the Multidimensional Analysis of Poverty. Econ. Notes Monte Dei Paschi Siena 24, 115–34.
Chung, H., van der Lippe, T., 2020. Flexible Working, Work–Life Balance, and Gender Equality: Introduction. Soc. Indic. Res. 151, 365–381. https://doi.org/10.1007/s11205-018-2025-x
Collins, L.M., Schafer, J.L., Kam, C.-M., 2001. A comparison of inclusive and restrictive strategies in modern missing data procedures. Psychol. Methods 6, 330–351. https://doi.org/10.1037/1082-989X.6.4.330
De Leonardis, O., Negrelli, S., Salais, R. (Eds.), 2012. Democracy and capabilities for voice: welfare, work and public deliberation in Europe, New Edition. ed. Presses Interuniversitaires Europeennes, Brussels, Belgium.
De Munck, J., Ferreras, I., 2013. Restructuring processes and capability for voice: case study of Volkswagen, Brussels. Int. J. Manpow. 34, 397–412. https://doi.org/10.1108/IJM-05-2013-0099
Decancq, K., Lugo, M.A., 2013. Weights in Multidimensional Indices of Wellbeing: An Overview. Econom. Rev. 32, 7–34. https://doi.org/10.1080/07474938.2012.690641
Descatha, A., Fadel, M., Sembajwe, G., Peters, S., Evanoff, B.A., 2022. Job-Exposure Matrix: A Useful Tool for Incorporating Workplace Exposure Data Into Population Health Research and Practice. Front. Epidemiol. 2. https://doi.org/10.3389/fepid.2022.857316
Deutsch, J., Silber, J., 2005. Measuring Multidimensional Poverty: An Empirical Comparison of Various Approaches. Rev. Income Wealth 51, 145–174. https://doi.org/10.1111/j.1475-4991.2005.00148.x
DfE, 2020. Working Futures 2017-2027: Long-run labour market and skills projections for the UK. Department for Education, London, UK.
Epstein, C.F., Seron, C., Oglensky, B., Saute, R., 1999. The part-time paradox: time norms, professional lives, family, and gender. Routledge, New York, USA.
Esping-Andersen, G., 1996. Equality or employment? The interaction of wages, welfare states and family change. Transf. Eur. Rev. Labour Res. 2, 615–634. https://doi.org/10.1177/102425899600200405
Fisher, P., Fumagalli, L., Buck, N., Avram, S., 2019. Understanding Society and its income data (No. 2019- 08), Understanding Society Working Paper. University of Essex, Essex, UK.
Freeman, R.B., Medoff, J.L., 1992. What do unions do?, 5th Edition. ed. Basic Books, New York, USA.
Gallie, D. (Ed.), 2007. Employment regimes and the quality of work. Oxford University Press, Oxford, UK.
Gallie, D. (Ed.), 2004. Welfare regimes and the experience of unemployment in Europe. Oxford University Press, Oxford, UK.
Gallie, D., 2003. The Quality of Working Life: Is Scandinavia Different? Eur. Sociol. Rev. 19, 61–79. https://doi.org/10.1093/esr/19.1.61
Gallie, D., Felstead, A., Green, F., 2004. Changing Patterns of Task Discretion in Britain. Work Employ. Soc. 18, 243–266. https://doi.org/10.1177/09500172004042769
Gelman, A., 2008. Scaling regression inputs by dividing by two standard deviations. Stat. Med. 27, 2865–2873. https://doi.org/10.1002/sim.3107
Giupponi, G., Xu, X., 2020. What does the rise of self-employment tell us about the UK labour market? Institute for Fiscal Studies, London, UK.
González, P., Sehnbruch, K., Apablaza, M., Méndez Pineda, R., Arriagada, V., 2021. A Multidimensional Approach to Measuring Quality of Employment (QoE) Deprivation in Six Central American Countries. Soc. Indic. Res. 158, 107–141. https://doi.org/10.1007/s11205-021-02648-0

Graham, J.W., 2009. Missing data analysis: making it work in the real world. Annu. Rev. Psychol. 60, 549–576. https://doi.org/10.1146/annurev.psych.58.110405.085530
Greco, G., 2018. Setting the Weights: The Women's Capabilities Index for Malawi. Soc. Indic. Res. 135, 457–478. https://doi.org/10.1007/s11205-016-1502-3
Green, F., 2009. Job Quality in Britain (No. Issue No. 1 (November 2009)), Praxis. UK Commission for Employment and Skills, London, UK.
Green, F., 2001. It's Been A Hard Day's Night: The Concentration and Intensification of Work in Late Twentieth-Century Britain. Br. J. Ind. Relat. 39, 53–80. https://doi.org/10.1111/1467-8543.00189
Green, F., Felstead, A., Gallie, D., Henseke, G., 2022. Working Still Harder. Ind. Labor Relat. Rev. 75, 458–487. https://doi.org/10.1177/0019793920977850
Hirsch, D., 2015. Inflation and the minimum income standard: past and future measures (CRSP Working Paper 645). Centre for Research in Social Policy, Loughborough University, Loughborough, UK.
Hirschman, A.O., 1970. Exit, voice, and loyalty: responses to decline in firms, organizations, and states. Harvard University Press, Cambridge, MA, USA.
Hobson, B., 2011. The Agency Gap in Work–Life Balance: Applying Sen's Capabilities Framework Within European Contexts. Soc. Polit. Int. Stud. Gend. State Soc. 18, 147–167. https://doi.org/10.1093/sp/jxr012
HSE, 2021. Technical report: the impact of the coronavirus pandemic on the interpretation of health and safety statistics 2020/21. Health and Safety Executive, Bootle, UK.
Kahneman, D. (Ed.), 1999. Well-being: the foundations of hedonic psychology, 1st papercover ed. ed. Russell Sage Foundation, New York, USA.
Kahneman, D., Krueger, A.B., Schkade, D., Schwarz, N., Stone, A., 2004. Toward National Well-Being Accounts. Am. Econ. Rev. 94, 429–434. https://doi.org/10.1257/0002828041301713
Kalleberg, A.L., 2018. Precarious lives: job insecurity and well-being in rich democracies. Polity Press, Cambridge, UK.
Krishnakumar, J., Nagar, A.L., 2008. On Exact Statistical Properties of Multidimensional Indices Based on Principal Components, Factor Analysis, MIMIC and Structural Equation Models. Soc. Indic. Res. 86, 481–496.
Layard, R., 2011. Happiness: lessons from a new science, New Edition. ed, Penguin psychology. Penguin Books, London, UK.
Léné, A., 2019. Job Satisfaction and Bad Jobs: Why Are Cleaners So Happy at Work? Work Employ. Soc. 33. https://doi.org/10.1177/0950017019828904
Leschke, J., Watt, A., 2014. Challenges in constructing a multi-dimensional European job quality index. Soc. Indic. Res. 118, 1–31. https://doi.org/10.1007/s11205-013-0405-9
Lewis, J., Giullari, S., 2005. The adult worker model family, gender equality and care: the search for new policy principles and the possibilities and problems of a capabilities approach. Econ. Soc. 34, 76–104. https://doi.org/10.1080/0308514042000329342
Lindley, J., Machin, S., 2013. Wage inequality in the Labour years. Oxf. Rev. Econ. Policy 29, 165–177. https://doi.org/10.1093/oxrep/grt009
Lynn, P., 2011. Maintaining Cross-Sectional Representativeness in a Longitudinal General Population Survey (No. 2011– 04), Understanding Society Working Paper. Institute for Social and Economic Research, University of Essex, Essex, UK.
Lynn, P., Borkowska, M., 2018. Some Indicators of Sample Representativeness and Attrition Bias for BHPS and Understanding Society. Institute for Social and Economic Research, University of Essex, Essex, UK.
Machin, S., 2011. Changes in UK Wage Inequality Over the Last Forty Years, in: Gregg, P., Wadsworth, J. (Eds.), The Labour Market in Winter: The State of Working Britain. Oxford University Press, Oxford, UK. https://doi.org/10.1093/acprof:osobl/9780199587377.003.0012

McGillivray, M., 2005. Measuring Non-Economic Well-Being Achievement. Rev. Income Wealth 51, 337–364. https://doi.org/10.1111/j.1475-4991.2005.00157.x
Muñoz de Bustillo, R., Fernández Macías, E., 2005. Job satisfaction as an indicator of the quality of work. J. Socio-Econ. 34, 656–673. https://doi.org/10.1016/j.socec.2005.07.027
Muñoz de Bustillo, R., Fernandez-Macias, E., Anton, J.-I., Esteve, F., 2011. Measuring more than money: the social economics of job quality. Edward Elgar, Cheltenham, UK.
Noorbakhsh, F., 1998. The human development index: some technical issues and alternative indices. J. Int. Dev. 10, 589–605. https://doi.org/10.1002/(SICI)1099-1328(199807/08)10:5<589::AID-JID484>3.0.CO;2-S
Nussbaum, M.C., 2011. Creating capabilities: the human development approach. Belknap Press of Harvard University Press, Cambridge, MA, USA.
ONS, 2024. Average actual weekly hours of work for full-time workers (seasonally adjusted). Office for National Statistics, Newport, Wales, UK.
ONS, 2022. Employee workplace pensions in the UK: 2021 provisional and 2020 final results. Office for National Statistics, Newport, Wales, UK.
ONS, 2021. The relationship between Standard Occupational Classification 2010 and Standard Occupational Classification 2020. Office for National Statistics, Newport, Wales, UK.
ONS, 2017. Tenure and skill level of public and private sector employees in the United Kingdom. Office for National Statistics, Newport, Wales, UK.
Parasuraman, S., Simmers, C.A., 2001. Type of Employment, Work-Family Conflict and Well-Being: A Comparative Study. J. Organ. Behav. 22, 551–568.
Schokkaert, E., 2007. Capabilities and Satisfaction with Life. J. Hum. Dev. 8, 415–430. https://doi.org/10.1080/14649880701462239
Schokkaert, E., Verhofstadt, E., Ootegem, L., 2009. Measuring job quality and job satisfaction (No. 09/620). Working Papers of the Faculty of Economics and Business Administration, Ghent University, Ghent, Belgium.
Sehnbruch, K., 2004. From the quantity to the quality of employment: an application of the capability approach to the Chilean labour market (No. Report No. 9). University of California, Berkeley and Center for Latin American Studies, Berkley, CA, USA.
Sen, A., 1999. Development as Freedom. Oxford University Press, Oxford, UK.
Somarriba, N., Pena, B., 2009. Synthetic Indicators of Quality of Life in Europe. Soc. Indic. Res. 94, 115–133. https://doi.org/10.1007/s11205-008-9356-y
Stuart, E.A., Azur, M., Frangakis, C., Leaf, P., 2009. Multiple Imputation With Large Data Sets: A Case Study of the Children's Mental Health Initiative. Am. J. Epidemiol. 169, 1133–1139. https://doi.org/10.1093/aje/kwp026
Van Buuren, S., Groothuis-Oudshoorn, K., 2011. Mice: Multivariate Imputation by Chained Equations in R. J. Stat. Softw. 45, 1–67. https://doi.org/10.18637/jss.v045.i03
Vyas, S., Kumaranayake, L., 2006. Constructing socio-economic status indices: how to use principal components analysis. Health Policy Plan. 21, 459–468. https://doi.org/10.1093/heapol/czl029
Wilson, R., May-Gillings, M., Patel, S., Bui, H., 2020. Working Futures 2017-2027: Long-run labour market and skills projections for the UK - Technical report on sources and methods. Department for Education, London, UK.
